# Neuroticism and posttraumatic stress disorder: A Mendelian randomization analysis

**DOI:** 10.1002/brb3.70041

**Published:** 2024-09-30

**Authors:** Zifan You, Shanshan Chen, Jinsong Tang

**Affiliations:** ^1^ Department of Psychiatry, Sir Run Run Shaw Hospital Zhejiang University School of Medicine Hangzhou Zhejiang China

**Keywords:** depressed affect, mendelian randomization, neuroticism, posttraumatic stress disorder, sensitivity to environmental stress and adversity, worry

## Abstract

**Objective:**

Epidemiological studies revealed an unestablished association between neuroticism and posttraumatic stress disorder (PTSD) and we conducted mendelian randomization (MR) analyses to examine whether neuroticism clusters of worry, depressed affect, and sensitivity to environmental stress and adversity (SESA) were involved in the development of PTSD.

**Method:**

We obtained data on three neuroticism clusters, PTSD, and nine other psychiatric disorders from genome‐wide association studies summary statistics and employed univariable, multivariable, and mediation MR analyses to explore causal associations among them.

**Results:**

Neuroticism clusters were linked with PTSD (depressed affect (odds ratio [OR]: 2.94 [95% confidence interval: 2.21–3.92]); SESA (2.69 [1.95–3.71]; worry (1.81 [1.37–2.99])). Neuroticism clusters were also associated with psychiatric disorders, with the depressed effect on panic disorder (PD) (2.60 [1.14–5.91]), SESA on anorexia nervosa (AN) (2.77 [1.95–3.94]) and schizophrenia (2.55 [1.99–3.25]), worry on major depressive disorder (MDD) (2.58 [2.19–3.05]). In multivariable MR, only the SESA‐PTSD association remained (2.60 [2.096, 3.107]) while worry‐PTSD and depressed affect‐PTSD associations attenuated to nonsignificance. Mediation MR analyses suggested that PD mediated 3.76% of the effect of depressed effect on PTSD and AN mediated 10.33% of the effect of SESA on PTSD.

**Conclusion:**

Delving deeper into neuroticism clusters, we comprehensively understand the role of neuroticism in PTSD.

## Graphical abstract

This study employs Mendelian randomization to demonstrate that neuroticism subclusters (worry, SESA, and depressed affect) influence the risk of developing posttraumatic stress disorder (PTSD). The analysis identifies anorexia nervosa (AN) and panic disorder (PD) as mediating factors, illustrating the complex pathways through which neuroticism contributes to PTSD.

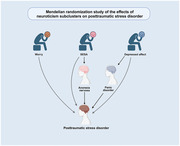

## 1 INTRODUCTION

Neuroticism, as an inherited personality trait and one of the main dimensions of the five‐factor personality model, is generally recognized to be linked to negative emotional experiences (Barlow et al., 2014; Redelmeier et al., 2021). The personality trait commonly manifests as anxiety, irritability, revulsion, discontentment, animosity, culpability, solitude, and fragility (Barlow et al., 2014). Prior research has documented robust connections between neuroticism and a range of psychiatric issues, such as schizophrenia (SCZ) (Van Os & Jones, 2001), major depressive disorder (MDD) (Bienvenu et al., 2004; Jylhä et al., 2009), bipolar disorder (BIP) (Quilty et al., 2009), autism spectrum disorder (ASD) (van Oosterhout et al., 2022), obsessive compulsory disorder (OCD) (Bienvenu et al., 2004), panic disorder (PD) (Forstner et al., 2021), posttraumatic stress disorder (PTSD) (Breslau & Schultz, 2013), attention‐deficit/hyperactivity disorder (ADHD) (Van Dijk et al., 2017), anorexia nervosa (AN) (Miller et al., 2006), and Tourette syndrome (TS) (O'Hare et al., 2015).

PTSD is a common mental health condition triggered by traumatic events, resulting in unyielding fear, intrusive memories, and intensified stress actions (Stein et al., 2014). Previous studies have underscored the critical role of traumatic experiences in the development of PTSD. Yet emerging evidence indicates that PTSD stems from a complex interaction involving both environmental factors like trauma and inherent personality characteristics like neuroticism (Cyniak‐Cieciura et al., 2022; Yin et al., 2019). Notably, research revealed the mediating effect of neuroticism on PTSD (Khamis, 2022). As neuroticism dimension scores increased, so did the likelihood of developing PTSD after experiencing trauma (Gale et al., 2016). However, even with these discoveries, the exact impact of neuroticism on PTSD development is still not fully determined. Several Mendelian randomization (MR) analyses have endeavored to explore potential causal links between neuroticism and PTSD, with no significant causal links discovered (Speed et al., 2019; Zhang et al., 2021). The majority of these studies have exclusively concentrated on the personality trait of neuroticism, resulting in a void of MR research concerning the underlying mechanism of various neuroticism clusters in PTSD. Generally, the Eysenck personality questionnaire with 12 items is employed to evaluate neuroticism, pinpointing a range of specific clusters such as worry, depressed affect, and sensitivity to environmental stress and adversity (SESA; Nagel et al., 2020; Nagel et al., 2018). Addressing this void, our study aimed to delve into the effects of such clusters as latent risk factors for PTSD from multiple perspectives, thereby deepening our comprehension of how these traits influence this disorder's development.

In our study, we employed MR analysis to assess the potential causal relationship between neuroticism clusters and psychiatric disorders. MR utilizes genetic variants as natural experiments, which are randomly distributed among subjects, providing a distinct opportunity to evaluate the relationship between exposures (neuroticism) and outcomes (psychiatric disorders; Lamina, 2022; Lawlor et al., 2008). Our selection focused on genetic instrumental variables (IVs) known to be closely linked with neuroticism in prior large‐scale genome‐wide association studies (GWAS) research. As the variants are identified prior to the development of outcomes, MR analysis bears the benefit of minimizing the likelihood of reverse causality (Gupta et al., 2017; Lawlor et al., 2008). Meanwhile, MR also addresses the confounding effect due to the random assignment of variants at birth and the minimal alterations of genotypes throughout the lifespan (Gupta et al., 2017; Lawlor et al., 2008). Consequently, MR can be utilized as an effective technique to deduce causality by combining the summary information of genetic variants.

Therefore, we applied a systematic two‐sample MR analysis to explore whether the three neuroticism clusters including depressed affect, worry, and SESA have a causal effect on PTSD and nine other common psychiatric disorders mentioned above including SCZ, MDD, BIP, PD, OCD, AN, ADHD, TS, and ASD and if so, whether such psychiatric disorders serve as the mediator in the progression from neuroticism to PTSD. Our study seeks to develop a more nuanced model of PTSD that accounts for intricate interplays, thus enhancing our understanding of such disease's various contributing factors.

## 2 MATERIALS AND METHODS

### 2.1 Study design

**FIGURE 1:**
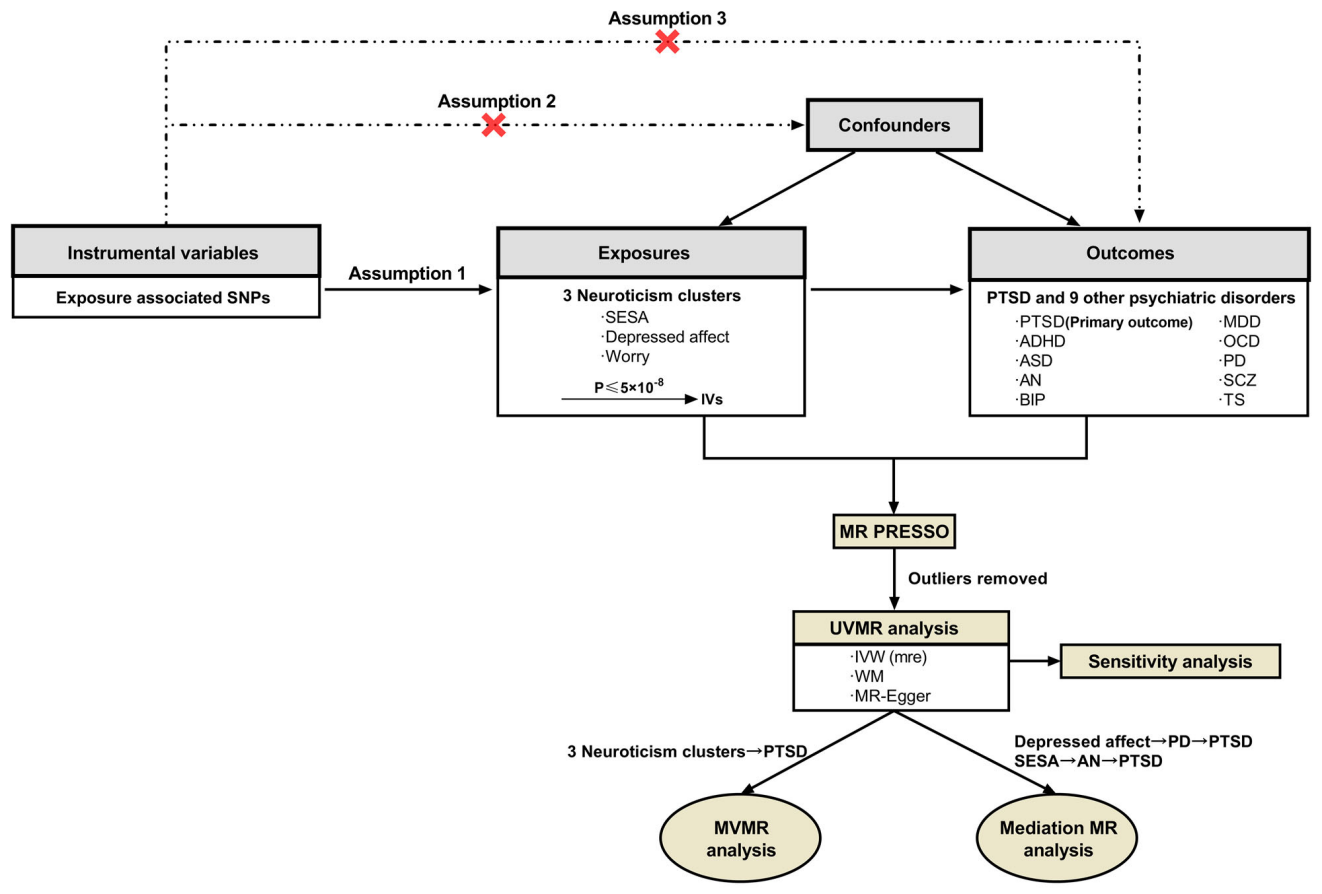
Workflow of the Mendelian randomization analysis. SESA, sensitivity to environmental stress and adversity; GWAS, genome‐wide association studies; ADHD, attention‐deficit/hyperactivity disorder; AN, anorexia nervosa; ASD, autism spectrum disorder; BIP, bipolar disorder; MDD, major depressive disorder; OCD, obsessive compulsory disorder; PTSD, posttraumatic stress disorder; TS, Tourette syndrome; SCZ, schizophrenia; PD, panic disorder; SNP, single‐nucleotide polymorphisms; PGC, psychiatric genomics consortium; IVW (mre), the inverse variance weighted (multiplicative random effects); WM, weighted median; MR PRESSO, MR pleiotropy residual sum and outlier; MVMR, multivariable mendelian randomization; UVMR, univariable mendelian randomization.

We performed a two‐sample MR analysis to explore the cause‐and‐effect link between neuroticism and psychiatric disorders, adhering to the fundamental principles of MR. These principles stipulate that (1) genetic IVs should have a robust correlation with the exposure; (2) these IVs should remain unaffected by any confounding factors affecting the result; (3) the outcome should be solely influenced by the exposure through the IVs (Figure 1; Lamina, 2022).

### 2.2 Data sources

For the 10 psychiatric disorders, we requested the European only summary statistics from the latest and largest available GWAS of the Psychiatric Genomics Consortium (assessed on July 13, 2023) and listed the detailed information in Table 1. The respective sample sizes were as follows: ADHD (38,691 cases and 186,843 controls; Demontis et al., 2023), AN (16,992 cases and 55,525 controls; Watson et al., 2019), ASD (18,382 cases and 27,969 controls; Grove et al., 2019), BIP (41,917 cases and 371,549 controls; Mullins et al., 2021), MDD (59,851 cases and 113,154 controls; Wray et al., 2018), OCD (2688 cases and 7037 controls; International Obsessive Compulsive Disorder Foundation Genetics Collaborative (IOCDF‐GC) & OCD Collaborative Genetics Association Studies (OCGAS), 2018), PTSD (30,000 cases and 170,000 controls; Nievergelt et al., 2019), SCZ (52,017 cases and 75,889 controls; Trubetskoy et al., 2022), PD (2248 cases and 7992 controls; Forstner et al., 2021), and TS (4819 cases and 9488 controls; Yu et al., 2019). To evaluate the genetic associations of neuroticism clusters including depressed affect (*n* = 357,957 individuals), SESA (*n* = 351,827 individuals), and worry (*n* = 348,291 individuals) on psychiatric disorders particularly PTSD, we employed the large available GWAS which was based on participants of the UK Biobank study (Nagel et al., 2020; Nagel et al., 2018). A comprehensive depiction of the exposure and outcome information can be found in the Supporting Information Material.

**TABLE 1:** Characteristics of selected genome‐wide association studies of psychiatric disorders.

Trials	Cases (*N*)	Controls (*N*)	Sample size (*N*)	No. Of SNPs	Population	Consortium	Year of publication	PMID
ADHD (Demontis et al., 2023)	38,691	186,843	225,534	6,774,224	European	PGC	2023	36702997
AN (Watson et al., 2019)	16,992	55,525	72,517	8,219,102	European	PGC	2019	31308545
ASD (Grove et al., 2019)	18,382	27,969	46,351	9,112,386	European	PGC	2019	30804558
BIP (Mullins et al., 2021)	41,917	371,549	413,466	7,608,183	European	PGC	2021	34002096
MDD (Wray et al., 2018)	59,851	113,154	173,005	13,554,550	European	PGC	2018	29700475
PTSD (Nievergelt et al., 2019)	30,000	170,000	200,000	9,766,174	European	PGC	2019	31594949
TS (Yu et al., 2019)	4819	9488	14,307	8,265,318	European	PGC	2019	30818990
SCZ (Trubetskoy et al., 2022)	52,017	75,889	59,906	7,659,767	European	PGC	2022	35396580
OCD (International Obsessive Compulsive Disorder Foundation Genetics Collaborative (IOCDF‐GC) and OCD Collaborative Genetics Association Studies (OCGAS), 2018)	2688	7037	9725	8,409,516	European	PGC	2018	28761083
PD (Forstner et al., 2021)	2248	7992	10,240	10,151,624	European	PGC	2021	31712720

Abbreviations: ADHD, attention‐deficit/hyperactivity disorder; AN, anorexia nervosa; ASD, autism spectrum disorder; BIP, bipolar disorder; MDD, major depressive disorder; OCD, obsessive compulsory disorder; PD, panic disorder; PGC, psychiatric genomics consortium; PTSD, posttraumatic stress disorder; SNP, single‐nucleotide polymorphisms; SCZ, schizophrenia; TS, Tourette syndrome.

### 2.3 Instrumental variable selection

To conduct the MR analysis, we rigorously selected the IVs with the assumptions of relevance, independence, and exclusion. First, for IVs on behalf of neuroticism clusters, we extracted SNPs with a genome‐wide significance threshold of *p* < 5 × 10^−8^. In mediation analysis, we relaxed the threshold to *P*<5 × 10^−6^ for psychiatric disorders without appropriate SNP screened except for SCZ and pooled nonoverlapping SNPs associated with exposure mediator to ensure the selection of independent IVs for mediation analysis. Second, to guarantee the independence of variants, the selected SNPs underwent strong linkage disequilibrium utilizing a European ancestry reference panel with *r* ^2 ^< 0.001 and physical window = 10,000 kb. Third, we ensured data harmonization and palindromic SNPs elimination with intermediate allele frequencies. Subsequently, the *F* statistic was calculated to evaluate the strength of the instruments with *F* > 10 avoiding weak IV bias (Sanderson & Windmeijer, 2016), utilizing the formula (*F* = beta^2^/se^2^) for exposures (Palmer et al., 2012), of which beta is the effect size of single SNP. R^2^ is the proportion of exposure explained by each SNP calculated with the following equation: *F* = *R* ^2^×(1*−R* ^2^)/(*N−*2), with N representing the sample size of the exposure data (Shim et al., 2015).

### 2.4 Statistical analysis

At the outset of our research, we employed two‐sample MR analyses to evaluate the casual links of neuroticism clusters on PTSD. Afterward, the same procedure was carried out between neuroticism clusters and other psychiatric disorders with the goal of discovering the possible pathways transferring from other psychiatric disorders to PTSD. Due to the highly accurate estimation of the inverse variance weighted (IVW), we employed it as the primary approach, supplemented by two other MR methods, MR‐Egger and weighed‐median (WM) to eliminate the influence of invalid IVs and pleiotropy of IVW and ensure the consistency of the causal direction (the detailed description of assumptions of the three MR analysis method were listed in Supporting Information Material; Burgess et al., 2013). Meanwhile, we detected the overlap among SNPs of different neuroticism clusters. Because of the nominally significant casual effects of three various neuroticism clusters on the same psychiatric disorder PTSD, we utilized multivariable mendelian randomization (MVMR) to regulate the pleiotropy arising from the overlap among the neuroticism clusters (Burgess & Thompson, 2015). Consistent with the univariable Mendelian randomization (UVMR) selection principles shown previously, we merged and reconstructed IVs with SNPs of depressed affect, SESA and worry clusters and employed the IVW MR technique for MVMR expansion. Based on UVMR, a two‐step MR analysis was performed to assess the mediation effect of one psychiatric disease on the association between one neuroticism cluster and PTSD with the proportion mediated calculated by coefficient product (Burgess et al., 2015).

### 2.5 Sensitivity analysis

To validate the reliability of the results, a series of sensitivity analyses were performed. First, we conducted MR pleiotropy residual sum and outlier (MR‐PRESSO) analysis to detect potential horizontal pleiotropy and excluded outliers to eliminate underlying pleiotropy (Verbanck et al., 2018). Furthermore, in the MR‐Egger intercept test, the intercept term deviating from zero indicates the existence of directional horizontal pleiotropy (Bowden et al., 2015). Second, Cochran's Q statistic of IVW and MR‐Egger method was performed to suggest the heterogeneity. Since we favored the multiplicative random effect rather than the fixed effect IVW, heterogeneity was permissible. Meanwhile, the funnel plot is indicative of heterogeneity through symmetry. In addition, the “leave‐one‐out” method confirmed that single SNPs did not affect the causal association. We defined the significance level at *p* < 0.05 among the above sensitivity analyses. In UVMR, we performed the Bonferroni correction for multiple comparisons through MR results and defined the significant *p*‐value threshold at *p*_IVW _< 0.05/30 = 0.0017 (3 neuroticism clusters‐10 psychiatric disorders correlations). Meanwhile, *p* < 0.05, surpassing the Bonferroni corrected threshold, indicates a nominally significant causal relationship. In MVMR and mediation MR analyses, a *p*‐value of less than 0.05 was deemed to be statistically significant. The statistical analyses were conducted with R4.1.1 and R packages “TwoSampleMR” and “MR‐PRESSO”.

## 3 RESULTS

### 3.1 Univariable and multivariable Mendelian randomization analysis

**FIGURE 2:**
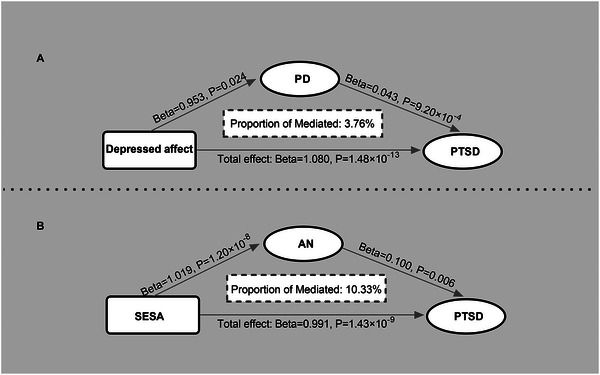
Summary of significant estimates of UVMR, MVMR, and mediation MR. (a) Significant estimates of UVMR. (b) Significant estimates of MVMR. (c) Significant estimates of mediation MR. The solid lines represent the nominally significant results in univariable, multivariable, and mediation MR. The dashed lines represent the nominally significant results in UVMR but null significant results in MVMR. The black lines represent the positive effects. AN, anorexia nervosa; MDD, major depressive disorder; MR, mendelian randomization; MVMR, multivariable mendelian randomization; PD, panic disorder; PTSD, posttraumatic stress disorder; SESA, sensitivity to environmental stress and adversity; SCZ, schizophrenia; UVMR, univariable mendelian randomization.

In the UVMR analyses for both the statistics of neuroticism clusters and psychiatric disorders, the F‐statistics exceed 10, ranging from 19.951 to 173.910. Details of the IVs in MR analyses were available in Table S1 and Table S2, probably indicating the nonexistence of substantial weak IVs bias. Among all the genetic association estimates between neuroticism clusters and psychiatric disorders, there were six pairs of traits with significant genetic correlations under Bonferroni‐adjusted level of significance (*p*<0.0017) and another pair of traits was nominally significant (*p*<0.05). Results are shown in Table 2, Table S3, and Figure 2. The IVW method results revealed that positive genetic association reflected causal effects of 43 SNPs determined SESA cluster on AN (odds ratio (OR) = 2.77, 95% confidential interval (CI) = (1.95, 3.94), *p* = 1.20 × 10^−8^), PTSD (OR = 2.69, 95%CI = (1.95, 3.71), *p* = 1.43 × 10^−9^), and SCZ (OR = 2.55, 95%CI = (1.99, 3.25), *p* = 6.72 × 10^−14^). Furthermore, 60 SNPs determined depressed affect cluster might contribute to PTSD (OR = 2.94, 95%CI = (2.21, 3.92), *p* = 1.48 × 10^−13^) and PD (OR = 2.59, 95%CI = (1.14, 5.91), *p* = 0.024). For the worry cluster with 59 SNPs, it has been estimated to have a casual correlation with MDD (OR = 2.58, 95%CI = (2.19, 3.05), *p* = 4.44 × 10^−29^) and PTSD (OR = 1.81, 95%CI = (1.37, 2.40), *p* = 3.61 × 10^−5^). On the basis of in‐depth analysis of causality between neuroticism clusters and multiple psychiatric disorders, we further investigated the potential association among various psychiatric disorders with clear causal links to the same neuroticism cluster. The results showed that an increase in PD (OR = 1.04, 95%CI = (1.02, 1.07), *p* = 9.20 × 10^−4^) and AN (OR = 1.11, 95%CI = (1.03, 1.19), *p* = 5.57 × 10^−3^) led to higher odds of PTSD (Table S4). In the MVMR analysis of considering the genetically predicted effects of multiple neuroticism clusters (depressed affect, worry, and SESA) on one psychiatric outcome (PTSD) simultaneously, only SESA had a significant causal effect on PTSD (OR = 2.60, 95%CI = (2.10, 3.11), *p* = 2.11 × 10^−4^) after the adjustment of worry and depressed affect clusters. While worry and depressed effects had null effects on PTSD in MVMR (Table S5).

**TABLE 2:** UVMR estimates for the causal associations of neuroticism clusters on psychiatric disorders.

Neuroticism cluster trait	Outcome	nSNP	*P*	OR (95%CI)	MR‐PRESSO Global test. *P*
Depressed affect	ADHD	53	4.55 × 10^−8^	2.145 (1.631,2.819)	<1 × 10^−4^
	AN	47	0.264	1.259 (0.840,1.888)	0.002
	ASD	48	0.361	1.190 (0.820,1.727)	6.0 × 10^−4^
	BIP	50	0.001	1.659 (1.223,2.250)	0.006
	MDD	47	3.58 × 10^−21^	2.844 (2.290,3.533)	0.001
	OCD	47	0.511	1.307 (0.588,2.904)	0.144
	PD	59	0.024	2.593 (1.137,5.913)	0.062
	PTSD	59	1.48 × 10^−13^	2.943 (2.210,3.919)	0.205
	TS	47	0.631	1.195 (0.578,2.473)	<1 × 10^−4^
	SCZ	41	0.002	1.614 (1.199,2.173)	<1 × 10^−4^
SESA	ADHD	40	0.027	1.386 (1.038,1.852)	<1 × 10^‐4^
	AN	39	1.20 × 10^−8^	2.772 (1.952,3.936)	0.051
	ASD	38	0.200	1.268 (0.882,1.822)	0.058
	BIP	38	0.059	1.464 (0.986,2.174)	<1 × 10^−4^
	MDD	38	9.49 × 10^−13^	2.267 (1.811,2.838)	0.016
	OCD	38	0.252	1.789 (0.661,4.842)	0.002
	PD	38	0.166	1.867 (0.771,4.519)	0.132
	PTSD	42	1.43 × 10^−9^	2.693 (1.954,3.711)	0.297
	TS	38	0.114	1.728 (0.876,3.407)	0.044
	SCZ	30	6.72 × 10^−14^	2.545 (1.993,3.249)	0.184
Worry	ADHD	52	0.707	1.041 (0.844,1.284)	0.704
	AN	50	0.017	0.656 (0.464,0.927)	<1 × 10^−4^
	ASD	52	0.388	1.165 (0.823,1.649)	6.00 × 10^−4^
	BIP	49	0.005	1.694 (1.176,2.439)	<1 × 10^−4^
	MDD	51	4.44 × 10^−29^	2.584 (2.188,3.052)	0.313
	OCD	51	0.006	3.026 (1.369,6.690)	0.014
	PD	51	2.76 × 10^−4^	4.626 (2.026,10.561)	0.002
	PTSD	58	3.61 × 10^−5^	1.810 (1.366,2.398)	0.169
	TS	50	0.008	1.811 (1.360,2.412)	0.012
	SCZ	41	0.001	2.310 (1.250,4.268)	<1e‐04

Abbreviations: ADHD, attention‐deficit/hyperactivity disorder; AN, anorexia nervosa; ASD, autism spectrum disorder; BIP, bipolar disorder; CI, confidence interval; MDD, major depressive disorder; MR‐PRESSO, MR pleiotropy residual sum and outlier; OCD, obsessive compulsory disorder; OR, odds ratio; PD, panic disorder; PTSD, posttraumatic stress disorder; SCZ, schizophrenia; SESA, sensitivity to environmental stress and adversity; SNP, single‐nucleotide polymorphisms; TS, Tourette syndrome; UVMR, univariable mendelian randomization.

### 3.2 Mediation Mendelian randomization analysis

**FIGURE 3:**
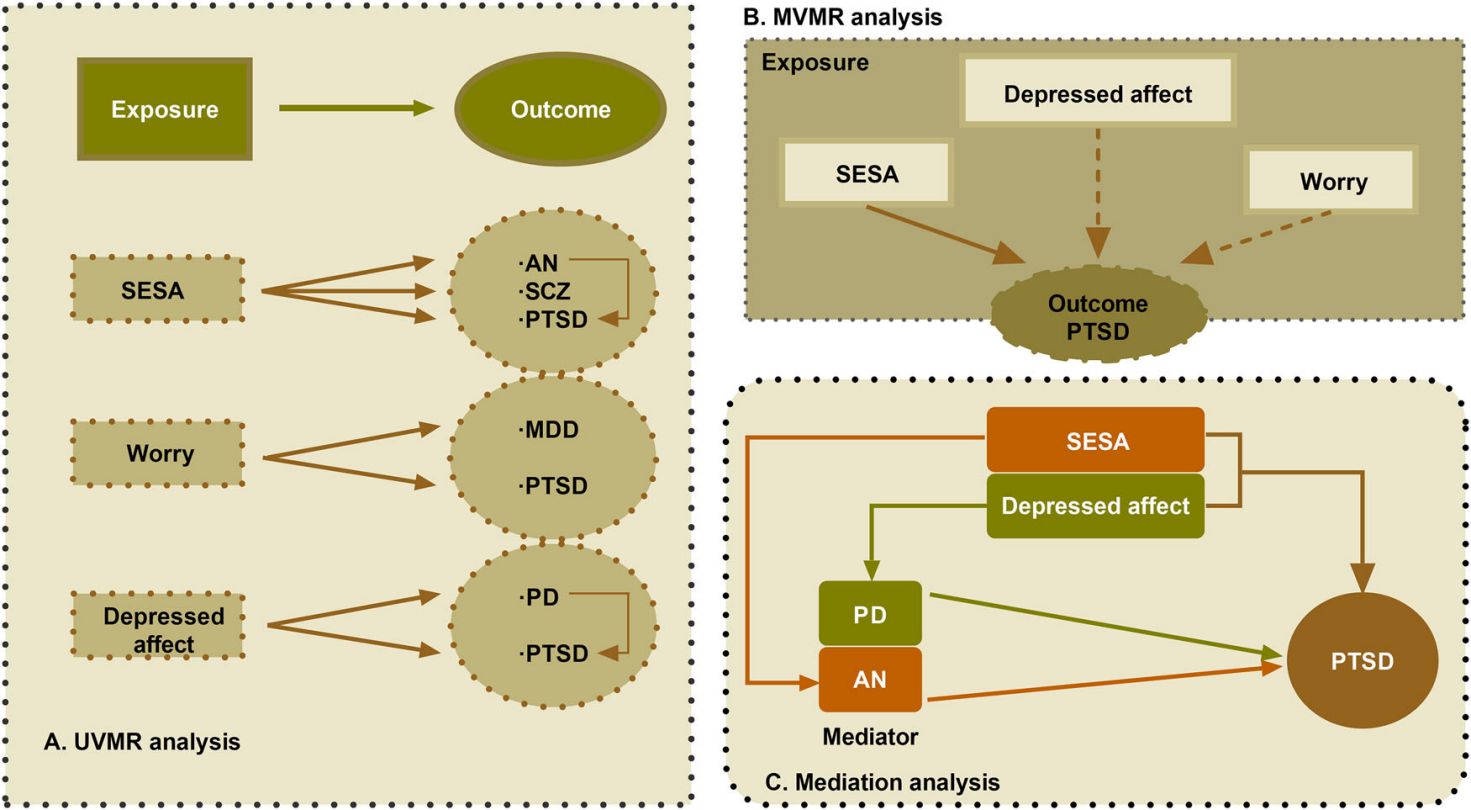
Two‐sample MR analyses of the significant causal effect of neuroticism clusters on psychiatric disorders. AN, anorexia nervosa; CI, confidence interval; MDD, major depressive disorder; OR, odds ratio; PD, panic disorder; PTSD, posttraumatic stress disorder; SCZ, schizophrenia; SESA, sensitivity to environmental stress and adversity; SNP, single nucleotide polymorphism.

**FIGURE 4:**
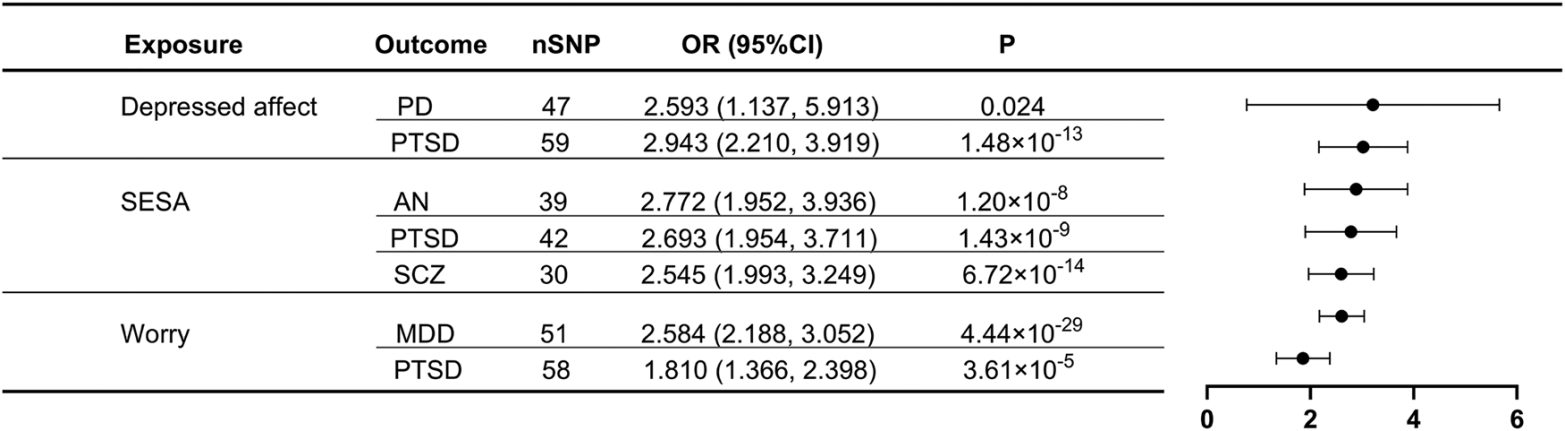
The results of mediation MR analysis. (a) The mediation MR estimates of depressed affect cluster on PTSD via PD. (b) The mediation MR estimates of SESA cluster on PTSD via AN. AN, anorexia nervosa; PD, panic disorder; PTSD, posttraumatic stress disorder; SESA, sensitivity to environmental stress and adversity.

After a series of processes for exploring associations between exposures of three neuroticism clusters and outcomes of 10 psychiatric disorders (Figure 1), we indicated significant causal effects among the depressed affect cluster, PD, and PTSD and among the SESA cluster, AN, and PTSD. Therefore, we performed mediation MR analyses to estimate the mediated role of PD between the depressed affect cluster and PTSD and AN between the SESA cluster and PTSD. The results revealed a significant mediating effect of PD in the depressed affect‐PTSD association with a 3.76% mediated ratio (Figure 3, Table S6). Furthermore, AN explained 10.33% of the total effect of the SESA cluster on PTSD (Figure 3, Table S6). The directions of the total effect were mainly consistent with the UVMR results. We respectively depicted the graphical representation of UVMR, MVMR, and mediation MR analyses with nominally significant results (*p*<0.05) in Figure 4.

### 3.3 Sensitivity analysis

Here we conducted sensitivity analyses to verify the assumed causal relationships derived from UVMR analyses (Tables S7–S10). Initially, the estimates obtained from the WM and MR‐Egger tests aligned with those obtained through the IVW method. What's more, the funnel plot demonstrated that the variation of effect size around the point estimate exhibited symmetry once outlier SNPs were eliminated (Figure S4–S6). The “leave‐one‐out” analysis assessed that single SNPs did not affect the causal association in our results (Figure S1–S3). According to the MR‐PRESSO, we recalculated the results of MR analyses after removing possible outliers (Table S9 and S10). Due to the evidence of pleiotropy, we dismissed the significant casual associations that had been estimated in UVMR analyses. For example, the causal effects of the depressed affect cluster on ADHD, BIP, and SCZ were discarded (*p*<0.05) for the existence of horizontal pleiotropy (Table 2). Meanwhile, no directional pleiotropy was identified by the MR‐Egger intercept. While Cochrane's *Q* test was indicative of heterogeneity for SESA‐AN association (*P*_MR‐Egger_ = 0.04, *P*_IVW_ = 0.02) and worry‐AN association (*P*_MR‐Egger_ = 2.88×10^−4^, *P*_IVW_ = 3.12×10^−4^) and the random effect IVW made such situation acceptable.

## 4 DISCUSSION

For a more profound insight into the impact of neuroticism on the development of PTSD, we reviewed several pivotal longitudinal studies. One study reveals that individuals who initially exhibited low PTSD symptoms after a disaster but later developed delayed PTSD displayed higher levels of neuroticism (Heir et al., 2021). This trait may lead these individuals to recall traumatic events more clearly, thus elevating their likelihood of experiencing delayed PTSD. Additionally, another three studies further support this finding by highlighting neuroticism's crucial role in sustaining PTSD symptoms, especially observing that people with elevated neuroticism levels suffered prolonged and severe PTSD symptoms after trauma events like the earthquake (Breslau & Schultz, 2013; Li et al., 2020; Ogle et al., 2017). Nonetheless, despite the valuable insights offered by these longitudinal studies, some studies investigating the link between neuroticism and PTSD employ cross‐sectional designs, impeding a thorough grasp of causality between them (Cyniak‐Cieciura et al., 2022; Gale et al., 2016; Khamis, 2022; Yin et al., 2019). Therefore, our study employs the MR to further investigate the potential causal relationship between neuroticism clusters and PTSD as well as other psychiatric disorders. We observed noteworthy positive links between SESA and AN, SCZ as well as PTSD, between worry and MDD, PTSD as well as between depressed affect and PD, PTSD. The stability of our results in different MR techniques has been remarkable, effectively eradicating any hindrance caused by horizontal pleiotropy. Subsequent MVMR analysis unveiled the consistent role of SESA in PTSD among three distinct neuroticism clusters. In addition, mediation analysis was performed to explore the potential pathways from different neuroticism clusters to PTSD, discovering for the first time that the effect of SESA on PTSD is partially driven by AN whereas the influence of depressed effect on PTSD is partly driven by PD. These findings substantiate the consistent alignment with previous research, thereby underscoring the pivotal role played by diverse neuroticism clusters in the emergence of PTSD.

The depressed affect cluster denotes various emotional reactions associated with depression frequently observed in people exhibiting high neuroticism. Such emotional responses encompass prolonged sadness, a sense of powerlessness, and a prevailing pessimistic outlook on life (Schipper et al., 2010). Previous studies revealed that a robust association between depression and PTSD might be a result of heightened susceptibility to psychological distress after traumatic events (Auxéméry, 2012). Depressive emotions, by potentially hindering the ability to cope with traumatic incidents, may unintentionally amplify the concentration of traumatic memories (Ashbaugh et al., 2018), thus intensifying the emergence of PTSD symptoms. Furthermore, the effect of depression may be felt in the physical body, affecting complex biological processes such as immune and inflammatory reactions, consequently elevating the chances of PTSD (Hoerster et al., 2019; Katrinli et al., 2022). Moreover, recent studies have highlighted the growing awareness of the neurobiological basis of depression and its link to PTSD. The role of neuroendocrine pathways and neurotransmitter imbalances, such as dysregulation in the hypothalamic‐pituitary‐adrenal (HPA) axis and aberrant serotonin signaling, in individuals with depressed affect, has been elucidated by these studies (Levy & Tasker, 2012; Sherin & Nemeroff, 2011). The presence of these neurobiological processes not only increases the likelihood of PTSD but also contributes to the persistence of symptoms and impedes the healing process (Sherin & Nemeroff, 2011). Additionally, reproductive behavior phenotypes have genetic overlaps with risk for PD and PTSD, which suggests that there is a shared genetic component that contributes to both PD and PTSD (Ohi et al., 2022), partially elucidating the underlying mechanism transferring from depressed affect cluster to PTSD through PD.

Neuroticism is a multifaced personality trait characterized by the SESA cluster, which is marked by a distaste for social gatherings, a reluctance to engage with others, and the experience of complex social situations, with a fascinating connection with PTSD (Nagel et al., 2020). This more nuanced connection encourages a deeper analysis of the fundamental mechanisms. People with a high level of SESA are more likely to stay away from social gatherings due to their heightened sensitivity to external pressures and interpersonal disputes (Georgiades et al., 2023). By avoiding social interactions, individuals may suffer from PTSD due to a lack of access to essential social support and emotional interconnections (Gros et al., 2016). Studies have demonstrated that social support is essential in mitigating the consequences of trauma and fostering resilience (Calhoun et al., 2022). Furthermore, the avoidance of social interaction could conceivably curtail the exchange of distressing events, thus limiting the chances for emotional release, which in turn can augment the aggravation of PTSD symptoms (Farnsworth & Sewell, 2011). In addition, SESA may increase the mental and emotional effects of PTSD by amplifying the understanding of social complexities. Individuals with a heightened sense of social security tend to be more inclined to contemplate social situations and occurrences. The tendency to ruminate excessively can result in heightened levels of stress and anxiety, which combined with being exposed to traumatic events, may be a fact in the emergence of PTSD (Keane et al., 2006).

Worry cluster, another major component of neuroticism, is characterized by a long‐term inclination to experience excessive and intrusive worry (M. Nagel et al., 2018). Individuals with worry, by their nature, generally concentrated on monotonous and calamitous thoughts of potential future dangers and unfavorable consequences of diverse circumstances (McEvoy et al., 2013). Such a neuroticism cluster associated with PTSD has a major influence on how people react to traumatic experiences. The constant concern can lead to PTSD symptoms becoming more severe and persistent, as it hinders the ability to process and resolve the trauma, potentially resulting in the reaction of distressing and intrusive memories. A thorough examination of the complex links between the worry cluster and PTSD uncovers numerous essential pathways including cognitive, neurological, and physiological processes. The worry cluster can have a major impact on the emergence of PTSD by amplifying cognitive prejudices (Chemtob et al., 1988). People who tend to be overly anxious are likely to be biased in their thinking, such as overestimating the risks involved (Newman et al., 2013). The presence of these biases may result in a heightened awareness of trauma‐related stimuli, thus strengthening the link between traumatic events and negative feelings, which is a hallmark of PTSD (Newman et al., 2013). Besides, the worry cluster has been linked to neuroimaging changes in the area of the prefrontal cortex, which is accountable for executive functions, emotional control, and threats, with the lack of regulation making people feel intense emotions and hinder their capacity to develop effective coping mechanisms (Kenwood et al., 2022; McEvoy et al., 2013). What's more, an extended period of anxiety has been linked to physiological tension reactions and disturbances in the HPA axis, which have an impact on the body's stress control mechanisms, the same as depressed affect (Levy & Tasker, 2012).

To sum up, the link between neuroticism and PTSD is intricate, and a variety of factors may be responsible for this connection. More studies are necessary to gain a deeper comprehension of the connections, as the information can be beneficial in guiding therapeutic strategies and interventions for people who are prone to PTSD. By studying how neuroticism of different subtypes affects the progression of PTSD, clinicians can more accurately identify individuals at high risk and tailor personalized interventions accordingly to alleviate their symptoms and improve their quality of life.

Despite our research strongly supporting a causal link between neuroticism clusters and PTSD, several potential limitations should be noted. First, the GWAS datasets used in this study were all of European ancestry. Therefore, examining these associations among diverse ethnic groups and cultural heritages would shed light on the universal applicability of the observed relationships or their substantial variation across different groups. Furthermore, despite the application of sensitivity analyses such as MR‐Egger and MR‐PRESSO, it is still possible that confounding bias may exist. Also, since there are fewer GWAS studies associated with neuroticism clusters, a larger sample is imperative to verify the reliability of chance results. Additionally, employing large cohort studies plays a crucial role in confirming the solidity of our MR results. These investigations can provide longitudinal data to solidify causal deductions and tackle potential changes in variables over time. Eventually, we're supposed to make an effort to further analyze and explain the mechanism of specific causal pathway associations. Exploring these paths allows subsequent studies to expand upon our preliminary results, possibly resulting in more focused and efficacious mental health treatments centered on neuroticism and its interaction with other psychological elements.

## 5 CONCLUSION

Our research provides evidence for a causal relationship between neuroticism clusters and psychiatric conditions. We demonstrate that the presence of the three neuroticism clusters was positively associated with a heightened likelihood of developing PTSD, with AN and PD potentially serving as mediators in such relationships. Hence, it is evident that neuroticism remains a significant healthcare burden and is worth emphasizing its role implicated in the progression and pathological manifestations of psychiatric disorders.

## AUTHOR CONTRIBUTIONS

**Zifan You**: Conceptualization; methodology; data curation; formal analysis; writing—original draft; writing—review and editing. **Shanshan Chen**: Writing—review and editing; validation. **Jinsong Tang**: Conceptualization; validation; funding acquisition; writing—review and editing.

## CONFLICT OF INTEREST STATEMENT

The authors declare no conflicts of interest.

### PEER REVIEW

The peer review history for this article is available at https://publons.com/publon/10.1002/brb3.70041.

## PERMISSION TO REPRODUCE FROM OTHER SOURCES

All data are publicly available, so no permission is required.

## Supporting information



Supporting Information

## Data Availability

The summary GWAS data for psychiatric disorders and neuroticism were obtained from the PGC—Psychiatric Genomics Consortium (unc.edu) and GWAS Summary Statistics | CTG (cncr.nl), respectively.

## References

[brb370041-bib-0001] Ashbaugh, A. R. , Marinos, J. , & Bujaki, B. (2018). The impact of depression and PTSD symptom severity on trauma memory. Memory (Hove, England), 26(1), 106–116. 10.1080/09658211.2017.1334801 28566056

[brb370041-bib-0002] Auxéméry, Y. (2012). [Posttraumatic stress disorder (PTSD) as a consequence of the interaction between an individual genetic susceptibility, a traumatogenic event and a social context]. L'Encephale, 38(5), 373–380. 10.1016/j.encep.2011.12.003 23062450

[brb370041-bib-0003] Barlow, D. H. , Ellard, K. K. , Sauer‐Zavala, S. , Bullis, J. R. , & Carl, J. R. (2014). The origins of neuroticism. Perspectives on Psychological Science: A Journal of the Association for Psychological Science, 9(5), 481–496. 10.1177/1745691614544528 26186755

[brb370041-bib-0004] Bienvenu, O. J. , Samuels, J. F. , Costa, P. T. , Reti, I. M. , Eaton, W. W. , & Nestadt, G. (2004). Anxiety and depressive disorders and the five‐factor model of personality: A higher‐ and lower‐order personality trait investigation in a community sample. Depression and Anxiety, 20(2), 92–97. 10.1002/da.20026 15390211

[brb370041-bib-0005] Bowden, J. , Davey Smith, G. , & Burgess, S. (2015). Mendelian randomization with invalid instruments: Effect estimation and bias detection through Egger regression. International Journal of Epidemiology, 44(2), 512–525. 10.1093/ije/dyv080 26050253 PMC4469799

[brb370041-bib-0006] Breslau, N. , & Schultz, L. (2013). Neuroticism and post‐traumatic stress disorder: A prospective investigation. Psychological Medicine, 43(8), 1697–1702. 10.1017/S0033291712002632 23199934

[brb370041-bib-0007] Burgess, S. , Butterworth, A. , & Thompson, S. G. (2013). Mendelian randomization analysis with multiple genetic variants using summarized data. Genetic Epidemiology, 37(7), 658–665. 10.1002/gepi.21758 24114802 PMC4377079

[brb370041-bib-0008] Burgess, S. , Daniel, R. M. , Butterworth, A. S. , & Thompson, S. G. , & EPIC‐InterAct Consortium . (2015). Network Mendelian randomization: Using genetic variants as instrumental variables to investigate mediation in causal pathways. International Journal of Epidemiology, 44(2), 484–495. 10.1093/ije/dyu176 25150977 PMC4469795

[brb370041-bib-0009] Burgess, S. , & Thompson, S. G. (2015). Multivariable Mendelian randomization: The use of pleiotropic genetic variants to estimate causal effects. American Journal of Epidemiology, 181(4), 251–260. 10.1093/aje/kwu283 25632051 PMC4325677

[brb370041-bib-0010] Calhoun, C. D. , Stone, K. J. , Cobb, A. R. , Patterson, M. W. , Danielson, C. K. , & Bendezú, J. J. (2022). The role of social support in coping with psychological trauma: An integrated biopsychosocial model for posttraumatic stress recovery. The Psychiatric Quarterly, 93(4), 949–970. 10.1007/s11126-022-10003-w 36199000 PMC9534006

[brb370041-bib-0011] Chemtob, C. , Roitblat, H. L. , Hamada, R. S. , Carlson, J. G. , & Twentyman, C. T. (1988). A cognitive action theory of post‐traumatic stress disorder. Journal of Anxiety Disorders, 2(3), 253–275. 10.1016/0887-6185(88)90006-0

[brb370041-bib-0012] Cyniak‐Cieciura, M. , Popiel, A. , Kendall‐Tackett, K. , & Zawadzki, B. (2022). Neuroticism and PTSD symptoms: Gender moderates the mediating effect of peritraumatic emotions and dissociation. Psychological Trauma: Theory, Research, Practice and Policy, 14(3), 462–470. 10.1037/tra0001065 34410814

[brb370041-bib-0013] Demontis, D. , Walters, G. B. , Athanasiadis, G. , Walters, R. , Therrien, K. , Nielsen, T. T. , Farajzadeh, L. , Voloudakis, G. , Bendl, J. , Zeng, B. , Zhang, W. , Grove, J. , Als, T. D. , Duan, J. , Satterstrom, F. K. , Bybjerg‐Grauholm, J. , Bækved‐Hansen, M. , Gudmundsson, O. O. , Magnusson, S. H. , … Børglum, A. D. (2023). Genome‐wide analyses of ADHD identify 27 risk loci, refine the genetic architecture and implicate several cognitive domains. Nature Genetics, 55(2), 198–208. 10.1038/s41588-022-01285-8 36702997 PMC10914347

[brb370041-bib-0014] Farnsworth, J. K. , & Sewell, K. W. (2011). Fear of emotion as a moderator between PTSD and firefighter social interactions. Journal of Traumatic Stress, 24(4), 444–450. 10.1002/jts.20657 21780188

[brb370041-bib-0015] Forstner, A. J. , Awasthi, S. , Wolf, C. , Maron, E. , Erhardt, A. , Czamara, D. , Eriksson, E. , Lavebratt, C. , Allgulander, C. , Friedrich, N. , Becker, J. , Hecker, J. , Rambau, S. , Conrad, R. , Geiser, F. , McMahon, F. J. , Moebus, S. , Hess, T. , Buerfent, B. C. , … Schumacher, J. (2021). Genome‐wide association study of panic disorder reveals genetic overlap with neuroticism and depression. Molecular Psychiatry, 26(8), 4179–4190. 10.1038/s41380-019-0590-2 31712720

[brb370041-bib-0016] Gale, C. R. , Hagenaars, S. P. , Davies, G. , Hill, W. D. , Liewald, D. C. M. , Cullen, B. , Penninx, B. W. , International Consortium for Blood Pressure GWAS, CHARGE Consortium Aging and Longevity Group . Boomsma, D. I. , Pell, J. , McIntosh, A. M. , Smith, D. J. , & Harris, S. E. . (2016). Pleiotropy between neuroticism and physical and mental health: Findings from 108 038 men and women in UK Biobank. Translational Psychiatry, 6(4), e791–e791. 10.1038/tp.2016.56 27115122 PMC4872414

[brb370041-bib-0017] Georgiades, A. , Almuqrin, A. , Rubinic, P. , Mouhitzadeh, K. , Tognin, S. , & Mechelli, A. (2023). Psychosocial stress, interpersonal sensitivity, and social withdrawal in clinical high risk for psychosis: A systematic review. Schizophrenia (Heidelberg, Germany), 9(1), 38. 10.1038/s41537-023-00362-z 37330526 PMC10276848

[brb370041-bib-0018] Gros, D. F. , Flanagan, J. C. , Korte, K. J. , Mills, A. C. , Brady, K. T. , & Back, S. E. (2016). Relations among social support, PTSD symptoms, and substance use in veterans. Psychology of Addictive Behaviors: Journal of the Society of Psychologists in Addictive Behaviors, 30(7), 764–770. 10.1037/adb0000205 27786511 PMC5507582

[brb370041-bib-0019] Grove, J. , Ripke, S. , Als, T. D. , Mattheisen, M. , Walters, R. K. , Won, H. , Pallesen, J. , Agerbo, E. , Andreassen, O. A. , Anney, R. , Awashti, S. , Belliveau, R. , Bettella, F. , Buxbaum, J. D. , Bybjerg‐Grauholm, J. , Bækvad‐Hansen, M. , Cerrato, F. , Chambert, K. , Christensen, J. H. , … Børglum, A. D. (2019). Identification of common genetic risk variants for autism spectrum disorder. Nature Genetics, 51(3), 431–444. 10.1038/s41588-019-0344-8 30804558 PMC6454898

[brb370041-bib-0020] Gupta, V. , Walia, G. K. , & Sachdeva, M. P. (2017). “Mendelian randomization”: An approach for exploring causal relations in epidemiology. Public Health, 145, 113–119. 10.1016/j.puhe.2016.12.033 28359378

[brb370041-bib-0021] Heir, T. , Hussain, A. , Kristensen, P. , & Weisæth, L. (2021). Delayed post‐traumatic stress and memory inflation of life‐threatening events following a natural disaster: Prospective study. BJPsych Open, 7(4), e132. 10.1192/bjo.2021.955 34253278 PMC8281038

[brb370041-bib-0022] Hoerster, K. D. , Campbell, S. , Dolan, M. , Stappenbeck, C. A. , Yard, S. , Simpson, T. , & Nelson, K. M. (2019). PTSD is associated with poor health behavior and greater body mass index through depression, increasing cardiovascular disease and diabetes risk among U.S. veterans. Preventive Medicine Reports, 15, 100930. 10.1016/j.pmedr.2019.100930 31338278 PMC6627033

[brb370041-bib-0023] International Obsessive Compulsive Disorder Foundation Genetics Collaborative (IOCDF‐GC) and OCD Collaborative Genetics Association Studies (OCGAS) . (2018). Revealing the complex genetic architecture of obsessive‐compulsive disorder using meta‐analysis. Molecular Psychiatry, 23(5), 1181–1188. 10.1038/mp.2017.154 28761083 PMC6660151

[brb370041-bib-0024] Jylhä, P. , Melartin, T. , & Isometsä, E. (2009). Relationships of neuroticism and extraversion with axis I and II comorbidity among patients with DSM‐IV major depressive disorder. Journal of Affective Disorders, 114(1–3), 110–121. 10.1016/j.jad.2008.06.011 18687471

[brb370041-bib-0025] Katrinli, S. , Oliveira, N. C. S. , Felger, J. C. , Michopoulos, V. , & Smith, A. K. (2022). The role of the immune system in posttraumatic stress disorder. Translational Psychiatry, 12(1), 313. 10.1038/s41398-022-02094-7 35927237 PMC9352784

[brb370041-bib-0026] Keane, T. M. , Marshall, A. D. , & Taft, C. T. (2006). Posttraumatic stress disorder: Etiology, epidemiology, and treatment outcome. Annual Review of Clinical Psychology, 2, 161–197. 10.1146/annurev.clinpsy.2.022305.095305 17716068

[brb370041-bib-0027] Kenwood, M. M. , Kalin, N. H. , & Barbas, H. (2022). The prefrontal cortex, pathological anxiety, and anxiety disorders. Neuropsychopharmacology: Official Publication of the American College of Neuropsychopharmacology, 47(1), 260–275. 10.1038/s41386-021-01109-z 34400783 PMC8617307

[brb370041-bib-0028] Khamis, V. (2022). Neuroticism as mediator and moderator between war atrocities and psychopathology in Syrian refugee children and adolescents. Frontiers in Psychology, 13, 811920. 10.3389/fpsyg.2022.811920 35153961 PMC8829386

[brb370041-bib-0029] Lamina, C. (2022). Mendelian randomization: Principles and its usage in Lp(a) research. Atherosclerosis, 349, 36–41. 10.1016/j.atherosclerosis.2022.04.013 35606074

[brb370041-bib-0030] Lawlor, D. A. , Harbord, R. M. , Sterne, J. A. C. , Timpson, N. , & Davey Smith, G. (2008). Mendelian randomization: Using genes as instruments for making causal inferences in epidemiology. Statistics in Medicine, 27(8), 1133–1163. 10.1002/sim.3034 17886233

[brb370041-bib-0031] Levy, B. H. , & Tasker, J. G. (2012). Synaptic regulation of the hypothalamic‐pituitary‐adrenal axis and its modulation by glucocorticoids and stress. Frontiers in Cellular Neuroscience, 6, 24. 10.3389/fncel.2012.00024 22593735 PMC3349941

[brb370041-bib-0032] Li, Y. , Lv, Q. , Li, B. , Luo, D. , Sun, X. , & Xu, J. (2020). The role of trauma experiences, personality traits, and genotype in maintaining posttraumatic stress disorder symptoms among child survivors of the Wenchuan earthquake. BMC Psychiatry [Electronic Resource], 20(1), 439. 10.1186/s12888-020-02844-1 32894097 PMC7487586

[brb370041-bib-0033] McEvoy, P. M. , Watson, H. , Watkins, E. R. , & Nathan, P. (2013). The relationship between worry, rumination, and comorbidity: Evidence for repetitive negative thinking as a transdiagnostic construct. Journal of Affective Disorders, 151(1), 313–320. 10.1016/j.jad.2013.06.014 23866301

[brb370041-bib-0034] Miller, J. L. , Schmidt, L. A. , Vaillancourt, T. , McDougall, P. , & Laliberte, M. (2006). Neuroticism and introversion: A risky combination for disordered eating among a non‐clinical sample of undergraduate women. Eating Behaviors, 7(1), 69–78. 10.1016/j.eatbeh.2005.07.003 16360625

[brb370041-bib-0035] Mullins, N. , Forstner, A. J. , O'Connell, K. S. , Coombes, B. , Coleman, J. R. I. , Qiao, Z. , Als, T. D. , Bigdeli, T. B. , Børte, S. , Bryois, J. , Charney, A. W ,, Drange, O. K. , Gandal, M. J. , Hagenaars, S. P. , Ikeda, M. , Kamitaki, N. , Kim, M. , Krebs, K. , Panagiotaropoulou, G. , … Andreassen, O. A. (2021). Genome‐wide association study of more than 40,000 bipolar disorder cases provides new insights into the underlying biology. Nature Genetics, 53(6), 817–829. 10.1038/s41588-021-00857-4 34002096 PMC8192451

[brb370041-bib-0036] Nagel, M. , Jansen, P. R. , Stringer, S. , Watanabe, K. , de Leeuw, C. A. , Bryois, J. , Savage, J. E. , Hammerschlag, A. R. , Skene, N. G. , Muñoz‐Manchado, A. B. , 23andMe Research Team . White, T. , Tiemeier, H. , Linnarsson, S. , Hjerling‐Leffler, J. , Polderman, T. J. C. , Sullivan, P. F. , … Posthuma, D. . (2018). Meta‐analysis of genome‐wide association studies for neuroticism in 449,484 individuals identifies novel genetic loci and pathways. Nature Genetics, 50(7), 920–927. 10.1038/s41588-018-0151-7 29942085

[brb370041-bib-0037] Nagel, M. , Speed, D. , van der Sluis, S. , & Østergaard, S. D. (2020). Genome‐wide association study of the sensitivity to environmental stress and adversity neuroticism cluster. Acta Psychiatrica Scandinavica, 141(5), 476–478. 10.1111/acps.13155 31972866

[brb370041-bib-0038] Newman, M. G. , Llera, S. J. , Erickson, T. M. , Przeworski, A. , & Castonguay, L. G. (2013). Worry and generalized anxiety disorder: A review and theoretical synthesis of evidence on nature, etiology, mechanisms, and treatment. Annual Review of Clinical Psychology, 9, 275–297. 10.1146/annurev-clinpsy-050212-185544 PMC4964851 23537486

[brb370041-bib-0039] Nievergelt, C. M. , Maihofer, A. X. , Klengel, T. , Atkinson, E. G. , Chen, C.‐Y. , Choi, K. W. , Coleman, J. R. I. , Dalvie, S. , Duncan, L. E. , Gelernter, J. , Levey, D. F. , Logue, M. W. , Polimanti, R. , Provost, A. C. , Ratanatharathorn, A. , Stein, M. B. , Torres, K. , Aiello, A. E. , Almli, L. M. , … Koenen, K. C. (2019). International meta‐analysis of PTSD genome‐wide association studies identifies sex‐ and ancestry‐specific genetic risk loci. Nature Communications, 10(1), 4558. 10.1038/s41467-019-12576-w PMC6783435 31594949

[brb370041-bib-0040] Ogle, C. M. , Siegler, I. C. , Beckham, J. C. , & Rubin, D. C. (2017). Neuroticism increases PTSD symptom severity by amplifying the emotionality, rehearsal, and centrality of trauma memories. Journal of Personality, 85(5), 702–715. 10.1111/jopy.12278 27517170 PMC6196079

[brb370041-bib-0041] O'Hare, D. , Eapen, V. , Helmes, E. , McBain, K. , Reece, J. , & Grove, R. (2015). Factors impacting the quality of peer relationships of youth with Tourette's syndrome. BMC Psychology, 3, 34. 10.1186/s40359-015-0090-3 26424471 PMC4589979

[brb370041-bib-0042] Ohi, K. , Kuramitsu, A. , Fujikane, D. , Takai, K. , Sugiyama, S. , & Shioiri, T. (2022). Shared genetic basis between reproductive behaviors and anxiety‐related disorders. Molecular Psychiatry, 27(10), 4103–4112. 10.1038/s41380-022-01667-8 35750798

[brb370041-bib-0043] Palmer, T. M. , Lawlor, D. A. , Harbord, R. M. , Sheehan, N. A. , Tobias, J. H. , Timpson, N. J. , Smith, G. D. , & Sterne, J. A. (2012). Using multiple genetic variants as instrumental variables for modifiable risk factors. Statistical Methods in Medical Research, 21(3), 223–242. 10.1177/0962280210394459 21216802 PMC3917707

[brb370041-bib-0044] Quilty, L. C. , Sellbom, M. , Tackett, J. L. , & Bagby, R. M. (2009). Personality trait predictors of bipolar disorder symptoms. Psychiatry Research, 169(2), 159–163. 10.1016/j.psychres.2008.07.004 19699536

[brb370041-bib-0045] Redelmeier, D. A. , Najeeb, U. , & Etchells, E. E. (2021). Understanding patient personality in medical care: Five‐factor model. Journal of General Internal Medicine, 36(7), 2111–2114. 10.1007/s11606-021-06598-8 33506393 PMC7840072

[brb370041-bib-0046] Sanderson, E. , & Windmeijer, F. (2016). A weak instrument F‐test in linear IV models with multiple endogenous variables. Journal of Econometrics, 190(2), 212–221. 10.1016/j.jeconom.2015.06.004 29129953 PMC5669336

[brb370041-bib-0047] Schipper, L. J. , Sollman, M. J. , & Berry, D. T. R. (2010). NEO personality inventory (NEO‐PI‐R). In The Corsini Encyclopedia of Psychology. (pp. 1–3). John Wiley & Sons, Ltd. 10.1002/9780470479216.corpsy0590

[brb370041-bib-0048] Sherin, J. E. , & Nemeroff, C. B. (2011). Post‐traumatic stress disorder: The neurobiological impact of psychological trauma. Dialogues in Clinical Neuroscience, 13(3), 263–278. 10.31887/DCNS.2011.13.2/jsherin 22034143 PMC3182008

[brb370041-bib-0049] Shim, H. , Chasman, D. I. , Smith, J. D. , Mora, S. , Ridker, P. M. , Nickerson, D. A. , Krauss, R. M. , & Stephens, M. (2015). A multivariate genome‐wide association analysis of 10 LDL subfractions, and their response to statin treatment, in 1868 Caucasians. PLoS ONE, 10(4), e0120758. 10.1371/journal.pone.0120758 25898129 PMC4405269

[brb370041-bib-0050] Speed, D. , Hemani, G. , Speed, M. S. , Major Depressive Disorder Working Group of the Psychiatric Genomics Consortium . Børglum, A. D. , & Østergaard, S. D. . (2019). Investigating the causal relationship between neuroticism and depression via Mendelian randomization. Acta Psychiatrica Scandinavica, 139(4), 395–397. 10.1111/acps.13009 30697695 PMC6426667

[brb370041-bib-0051] Stein, D. J. , McLaughlin, K. A. , Koenen, K. C. , Atwoli, L. , Friedman, M. J. , Hill, E. D. , Maercker, A. , Petukhova, M. , Shahly, V. , van Ommeren, M. , Alonso, J. , Borges, G. , de Girolamo, G. , de Jonge, P. , Demyttenaere, K. , Florescu, S. , Karam, E. G. , Kawakami, N. , Matschinger, H. , … Kessler, R. C. (2014). DSM‐5 and ICD‐11 definitions of posttraumatic stress disorder: Investigating “narrow” and “broad” approaches. Depression and Anxiety, 31(6), 494–505. 10.1002/da.22279 24894802 PMC4211431

[brb370041-bib-0052] Trubetskoy, V. , Pardiñas, A. F. , Qi, T. , Panagiotaropoulou, G. , Awasthi, S. , Bigdeli, T. B. , Bryois, J. , Chen, C. Y. , Dennison, C. A. , Hall, L. S. , Lam, M. , Watanabe, K. , Frei, O. , Ge, T. , Harwood, J. C. , Koopmans, F. , Magnusson, S. , Richards, A. L. , & Sidorenko, J. , … Schizophrenia Working Group of the Psychiatric Genomics Consortium . (2022). Mapping genomic loci implicates genes and synaptic biology in schizophrenia. Nature, 604(7906), 502–508. 10.1038/s41586-022-04434-5 35396580 PMC9392466

[brb370041-bib-0053] Van Dijk, F. E. , Mostert, J. , Glennon, J. , Onnink, M. , Dammers, J. , Vasquez, A. A. , Kan, C. , Verkes, R. J. , Hoogman, M. , Franke, B. , & Buitelaar, J. K. (2017). Five factor model personality traits relate to adult attention‐deficit/hyperactivity disorder but not to their distinct neurocognitive profiles. Psychiatry Research, 258, 255–261. 10.1016/j.psychres.2017.08.037 28844557

[brb370041-bib-0054] van Oosterhout, J. , van der Linden, K. , Simons, C. J. P. , van Amelsvoort, T. , & Marcelis, M. (2022). Exploring the autism spectrum: Moderating effects of neuroticism on stress reactivity and on the association between social context and negative affect. Development and Psychopathology, 34(4), 1366–1375. 10.1017/S0954579420002278 33745477

[brb370041-bib-0055] Van Os, J. , & Jones, P. B. (2001). Neuroticism as a risk factor for schizophrenia. Psychological Medicine, 31(6), 1129–1134. 10.1017/s0033291701004044 11513380

[brb370041-bib-0056] Verbanck, M. , Chen, C.‐Y. , Neale, B. , & Do, R. (2018). Publisher correction: Detection of widespread horizontal pleiotropy in causal relationships inferred from Mendelian randomization between complex traits and diseases. Nature Genetics, 50(8), 1196. 10.1038/s41588-018-0164-2 29967445

[brb370041-bib-0057] Watson, H. J. , Yilmaz, Z. , Thornton, L. M. , Hübel, C. , Coleman, J. R. I. , Gaspar, H. A. , Bryois, J. , Hinney, A. , Leppä, V. M. , Mattheisen, M. , Medland, S. E. , Ripke, S. , Yao, S. , Giusti‐Rodríguez, P. , Anorexia Nervosa Genetics Initiative . Hanscombe, K. B. , Purves, K. L. , Eating Disorders Working Group of the Psychiatric Genomics Consortium . Adan, R. A. H. , … Bulik, C. M. . (2019). Genome‐wide association study identifies eight risk loci and implicates metabo‐psychiatric origins for anorexia nervosa. Nature Genetics, 51(8), 1207–1214. 10.1038/s41588-019-0439-2 31308545 PMC6779477

[brb370041-bib-0058] Wray, N. R. , Ripke, S. , Mattheisen, M. , Trzaskowski, M. , Byrne, E. M. , Abdellaoui, A. , Adams, M. J. , Agerbo, E. , Air, T. M. , Andlauer, T. M. F. , Bacanu, S. A. , Bækvad‐Hansen, M. , Beekman, A. F. T. , Bigdeli, T. B. , Binder, E. B. , Blackwood, D. R. H. , Bryois, J. , Buttenschøn, H. N. , Bybjerg‐Grauholm, J. , … Major Depressive Disorder Working Group of the Psychiatric Genomics Consortium . (2018). Genome‐wide association analyses identify 44 risk variants and refine the genetic architecture of major depression. Nature Genetics, 50(5), 668–681. 10.1038/s41588-018-0090-3 29700475 PMC5934326

[brb370041-bib-0059] Yin, Q. , Wu, L. , Yu, X. , & Liu, W. (2019). Neuroticism predicts a long‐term PTSD after earthquake trauma: The moderating effects of personality. Frontiers in Psychiatry, 10, 657. 10.3389/fpsyt.2019.00657 31616324 PMC6763688

[brb370041-bib-0060] Yu, D. , Sul, J. H. , Tsetsos, F. , Nawaz, M. S. , Huang, A. Y. , Zelaya, I. , Illmann, C. , Osiecki, L. , Darrow, S. M. , Hirschtritt, M. E. , Greenberg, E. , Muller‐Vahl, K. R. , Stuhrmann, M. , Dion, Y. , Rouleau, G. , Aschauer, H. , Stamenkovic, M. , Schlögelhofer, M. , & Sandor, P. , … Tourette Association of America International Consortium for Genetics, the Gilles de la Tourette GWAS Replication Initiative, the Tourette International Collaborative Genetics Study, and the Psychiatric Genomics Consortium Tourette Syndrome Working Group . (2019). Interrogating the genetic determinants of Tourette's syndrome and other tic disorders through genome‐wide association studies. The American Journal of Psychiatry, 176(3), 217–227. 10.1176/appi.ajp.2018.18070857 30818990 PMC6677250

[brb370041-bib-0061] Zhang, F. , Baranova, A. , Zhou, C. , Cao, H. , Chen, J. , Zhang, X. , & Xu, M. (2021). Causal influences of neuroticism on mental health and cardiovascular disease. Human Genetics, 140(9), 1267–1281. 10.1007/s00439-021-02288-x 33973063

