# Supplementary material for: Neuroticism and posttraumatic stress disorder: A Mendelian randomization analysis
Source: Brain Behav. 2024 Sep 30;14(10):e70041. doi: 10.1002/brb3.70041 (PMC11440025; doi:10.1002/brb3.70041)
Supplement: Supplementary file 1 — Supporting Information [file BRB3-14-e70041-s001.docx]

**Supplementary material to**

**“Neuroticism and PTSD: A Mendelian Randomization analysis”**

**Content**

1. **Description of neuroticism clusters and psychiatric disorders**
2. **Assumptions of the 3 MR analysis method (IVW, MR-Egger, WM)**
3. **Supplementary Tables 1-10**

Table S1. Instrumental variable information used for Mendelian randomization on the association of neuroticism clusters on psychiatric disorders.

Table S2. Instrumental variable information used for Mendelian randomization on the association among psychiatric disorders based on the association of neuroticism clusters on psychiatric disorders.

Table S3. The results of Mendelian randomization on the association of neuroticism clusters

on psychiatric disorders.

Table S4. The results of Mendelian randomization on the association among psychiatric disorders.

Table S5. The results of multivariable Mendelian randomization.

Table S6. The results of mediation Mendelian randomization.

Table S7. Tests of heterogeneity for neuroticism clusters as exposures.

Table S8. Tests of heterogeneity for psychiatric disorders as exposures.

Table S9. Tests of pleiotropic effects for psychiatric disorders as exposures.

Table S10. Tests of pleiotropic effects for neuroticism clusters as exposures.

1. **Supplementary Figures 1-6**

Figure S1. Leave-one-out plot of significant results from SESA on psychiatric disorders.

Figure S2. Leave-one-out plot of significant results from depressed affect on psychiatric disorders.

Figure S3. Leave-one-out plot of significant results from worry on psychiatric disorders.

Figure S4. Funnel plot of significant results from SESA on psychiatric disorders.

Figure S5. Funnel plot of significant results from depressed affect on psychiatric disorders.

Figure S6. Funnel plot of significant results from worry on psychiatric disorders.

**5.R code**

**1.Description of neuroticism clusters and psychiatric disorders**

**1.1. Description of neuroticism clusters**

For neuroticism clusters including depressed affect, worry and SESA, we employed the large available GWAS which was based on participants of the UK Biobank study.(Nagel et al., 2020, 2018). Neuroticism is a highly heritable personality trait, characterized by emotions such as guilt, loneliness, embarrassment, and being easily hurt. Individuals with high levels of neuroticism are at increased risk of developing mental disorders, depression in particular. Neuroticism is commonly measured using the 12-item version of the Eysenck Personality Questionnaire (EPQ)(Eysenck et al., 1985; Speed et al., 2019). Of the 12 EPQ items of the neuroticism scale, “depressed affect” cluster was obtained by summing the score on the four items, “Do you often feel lonely?”, “Do you ever feel ‘just miserable’ for no reason?”, “Does your mood often go up and down?”, and “Do you often feel fed up?”. The GWAS for “worry cluster” was obtained by summing the score on these four items, “Are you a worrier?”, “Do you suffer from nerves?”, “Would you call yourself a nervous person?”, and “Would you call yourself tense or highly stung?”.Based on clustering of the pairwise genetic correlations, three of the remaining four questions were hypothesized, namely ‘Are your feelings easily hurt?’, ‘Do you worry too long after an embarrassing experience?’, and ‘Are you often troubled by feelings of guilt?’, define a third genetic cluster “sensitivity to environmental stress and adversity’ (SESA)”. Such a cluster would be of particular interest due to the likely role of environmental stress and adversity in the etiology of depression.

**1.2. Description of psychiatric disorders**

For psychiatric disorders, we requested the European only summary statistics from the latest and largest available GWAS of the Psychiatric Genomics Consortium (PGC) (assessed on 13^th^ July 2023). The respective sample sizes were as follows: attention-deficit/hyperactivity disorder (ADHD) (38,691 cases and 186,843 controls) (Demontis et al., 2023), anorexia nervosa (AN) (16,992 cases and 55,525 controls) (Watson et al., 2019), autism spectrum disorder (ASD) (18,382 cases and 27,969 controls) (Grove et al., 2019), bipolar disorder (BIP) (41,917 cases and 371,549 controls) (Mullins et al., 2021), major depressive disorder (MDD) (59,851 cases and 113,154 controls) (Wray et al., 2018), obsessive compulsory disorder (OCD) (2,688 cases and 7,037 controls) (International Obsessive Compulsive Disorder Foundation Genetics Collaborative (IOCDF-GC) and OCD Collaborative Genetics Association Studies (OCGAS), 2018), posttraumatic stress disorder (PTSD) (30,000 cases and 170,000 controls)(Nievergelt et al., 2019), schizophrenia (SCZ) (52,017 cases and 75,889 controls) (Trubetskoy et al., 2022), Tourette syndrome (TS) (4,819 cases and 9,488 controls) (Yu et al., 2019) and panic disorder (PD) (2,248 cases and 7,992 controls) (Forstner et al., 2021) (Table [1](https://translational-medicine.biomedcentral.com/articles/10.1186/s12967-023-04368-0#Tab1)).

Attention deficit/hyperactivity disorder (ADHD) is a neurodevelopmental disorder with a prevalence of around 5.3% in childhood and 2.8% in adulthood. The aetiology of ADHD involves a combination of genetic and environmental factors, with an estimated heritability of ∼70–80%. ADHD is characterized by a persistent pattern of inattentive, hyperactive and impulsive behaviour; however, its clinical presentation is heterogeneous, with a wide spectrum of severity and symptoms that often overlap with other conditions. Individuals with ADHD often have a poor cognitive performance in executive functions, such as response inhibition, vigilance, working memory or planning, personality profiles with low effortful control and high neuroticism, or emotion dysregulation problems such as irritability or temper outbursts. Up to 70–80% of ADHD patients suffer from comorbid disorders across their lifespan. which include other psychiatric conditions, such as major depressive, oppositional defiant, bipolar or substance use disorders, but also somatic diseases such as obesity, sleep disorders or migraine.

Autism spectrum disorder (ASD) is a heterogeneous group of neurodevelopmental conditions, characterized by difficulties in social communication and interaction, as well as abnormally limited, repetitive behaviors and interests. In recent years, the prevalence of ASD has increased significantly. The prevalence of ASD in children aged 3–17 years in the United States in 2016 was 2.76%. By 2020, the prevalence of ASD had increased to 3.49%. A nationwide multicenter population-based study (142,086) conducted in China during 2014–2016, and an estimated prevalence of 0.70% has been reported for children aged 6–12 years. The increasing prevalence of ASD is placing enormous pressure on health and social services, families and education. Currently, the etiology of ASD is not clear, but it is believed that genetic factors, environmental factors (such as mercury, radiation, diesel waste, etc.), changes in neural connections, perinatal factors (such as age of parents, maternal medication, infection, etc.) and postpartum factors (such as meningitis, low birth weight, etc.) are associated with the pathogenesis of ASD.

[Eating disorders](https://www.sciencedirect.com/topics/psychology/eating-disorders) (EDs) are important psychiatric disorders that involve abnormal eating or weight-control [behaviors](https://www.sciencedirect.com/topics/neuroscience/behavior-neuroscience). This condition is more common in females, in that approximately 92% of all individuals diagnosed with [AN](https://www.sciencedirect.com/topics/neuroscience/anorexia-nervosa) are women. Anorexia nervosa (AN), which is characterized by severely restrictive food consumption and extremely low body weight, is one of the two [major diagnostic categories](https://www.sciencedirect.com/topics/medicine-and-dentistry/major-diagnostic-category) of EDs. AN is associated with a weighted annual mortality rate of 5 per 1000 person-years, and an estimated lifetime prevalence of 0.3–0.9%. Furthermore, AN is characterized by an elevated incidence of other psychiatric disorders, and may progress to [chronic disease](https://www.sciencedirect.com/topics/psychology/chronic-disorder), resulting in high morbidity and mortality. Currently, there are no proven effective [treatments](https://www.sciencedirect.com/topics/medicine-and-dentistry/therapeutic-procedure) for AN that can normalize core symptoms.

Bipolar disorder is characterized by recurrent episodes of depression and mania or hypomania. Bipolar depressive episodes are similar to major depressive episodes. Manic and hypomanic episodes are characterized by a distinct change in mood and behavior during discrete time periods. The age of onset is usually between 15 and 25 years, and depression is the most frequent initial presentation. Approximately 75% of symptomatic time consists of depressive episodes or symptoms. Diagnosis and optimal treatment are often delayed by a mean of approximately 9 years following an initial depressive episode. Long-term treatment consists of mood stabilizers, such as lithium, valproate, and lamotrigine. More than 50% of patients with bipolar disorder are not adherent to treatment. Life expectancy is reduced by approximately 12 to 14 years in people with bipolar disorder, with a 1.6-fold to 2-fold increase in cardiovascular mortality occurring a mean of 17 years earlier compared with the general population. Prevalence rates of metabolic syndrome (37%), obesity (21%), cigarette smoking (45%), and type 2 diabetes (14%) are higher among people with bipolar disorder, contributing to the risk of early mortality. The annual suicide rate is approximately 0.9% among individuals with bipolar disorder, compared with 0.014% in the general population. Approximately 15% to 20% of people with bipolar disorder die by suicide.

[Major depressive disorder](https://www.sciencedirect.com/topics/medicine-and-dentistry/major-depressive-episode) (MDD) is a common psychiatric mood disorder, affecting over 280 million people worldwide (estimated 3.8% of the world population). Depression is associated with impaired social functioning and unemployment and is associated with a wide range of chronic physical illnesses, such as diabetes and cardiovascular disease. In addition, depression can lead to suicide, which is currently the fourth leading cause of death in the age of 15 to 29. MDD is expected to be the leading cause of overall [global burden of disease](https://www.sciencedirect.com/topics/medicine-and-dentistry/global-disease-burden) by the year 2030 (WHO). First-line [treatment](https://www.sciencedirect.com/topics/medicine-and-dentistry/therapeutic-procedure) options for MDD include antidepressants and forms of psychotherapy such as [cognitive behavioral therapy](https://www.sciencedirect.com/topics/medicine-and-dentistry/cognitive-behavioral-therapy) (CBT). However, approximately one third of patients with MDD do not respond adequately after receiving two or more evidence-based antidepressant treatment. Generally, the failure to respond to two or more medication trials of adequate dose and duration in MDD is referred to as [treatment resistant depression](https://www.sciencedirect.com/topics/medicine-and-dentistry/treatment-resistant-depression) (TRD).

Schizophrenia is a chronic psychiatric disorder with inconsistent behavioral and cognitive abnormalities. Based on conservative criteria, the prevalence of the disease is 0.7% but if more comprehensive diagnostic criteria are applied, it may be higher. The disease is not only associated with profound effects on the individual but also has consequences for society. Patients with schizophrenia show an impaired dopaminergic function, which is manifested in the acute psychotic state by an increase in dopamine synthesis, dopamine release, and resting-state synaptic dopamine concentrations. Imaging studies of the brain have shown that there is a subtle, almost universal decrease in gray matter, enlargement of the ventricles, and focal changes in white matter tracts in sufferers. The etiology of the disease is multifactorial and involves genetic and environmental factors. In addition, immunological theories related to the aetiopathogenesis of schizophrenia have been proposed in recent years.

Posttraumatic stress disorder (PTSD) is a syndrome that results from exposure to real or threatened death, serious injury, or sexual assault. Following the traumatic event, PTSD is common and is one of the serious health concerns that is associated with comorbidity, functional impairment, and increased mortality with suicidal ideations and attempts. The Diagnostic and Statistical Manual of Mental Disorders (DSM-5) has included PTSD in the new category of Trauma- and Stress-related Disorders.The symptoms of PTSD include persistently re-experiencing the traumatic event, intrusive thoughts, nightmares, flashbacks, dissociation (detachment from oneself or reality), and intense negative emotional (sadness, guilt) and physiological reaction on being exposed to the traumatic reminder. Furthermore, problems with sleep and concentration, irritability, increased reactivity, increased startle response, hypervigilance, avoidance of traumatic triggers also occur. There is a significant impairment in social, occupational, and other areas of functioning. However, the symptoms of PTSD overlap with acute stress disorder. For a patient to be diagnosed as PTSD, the duration of the symptoms must be more than one month.

Tourette syndrome (TS) is a neurodevelopmental disorder consisting of multiple motor and one or more vocal/phonic tics. TS is increasingly recognized as a common neuropsychiatric disorder usually diagnosed in early childhood and comorbid neuropsychiatric disorders occur in approximately 90% of patients, with attention deficit hyperactivity disorder (ADHD) and obsessive-compulsive disorder (OCD) being the most common ones. Moreover, a high prevalence of depression and personality disorders has been reported. Although the mainstream of tic management is represented by pharmacotherapy, different kinds of psychotherapy, along with neurosurgical interventions (especially deep brain stimulation, DBS) play a major role in the treatment of TS. The current diagnostic systems have dictated that TS is a unitary condition. However, recent studies have demonstrated that there may be more than one TS phenotype. In conclusion, it appears that TS probably should no longer be considered merely a motor disorder and, most importantly, that TS is no longer a unitary condition, as it was previously thought.

Panic disorder and panic attacks are two of the most common problems seen in the world of psychiatry. Panic disorder is a separate entity from panic attacks, although it is characterized by recurrent, unexpected panic attacks. Panic attacks are defined by the Diagnostic and Statistical Manual of Mental Health Disorders (DSM) as “an abrupt surge of intense fear or discomfort” reaching a peak within minutes. Four or more of a specific set of physical symptoms accompany a panic attack. These symptoms include; palpitations, pounding heart or accelerated heart rate, sweating, trembling or shaking, sensations of shortness of breath or smothering, feelings of choking, chest pain or discomfort, nausea or abdominal distress, feeling dizzy, unsteady, light-headedness, or faint, chills or heat sensations, paresthesias (numbness or tingling sensations), derealization (feelings of unreality) or depersonalization (being detached from oneself), fear of losing control or "going crazy," and fear of dying. Panic attacks occur as often as several times per day or as infrequently as only a few attacks per year. A hallmark feature of panic disorder is that attacks occur without warning. There is often no specific trigger for a panic attack. Patients suffering from these attacks self-perceive a lack of control. Panic attacks, however, are not limited to panic disorder. They can occur alongside other anxiety, mood, psychotic, and substance use disorder.

Obsessive-compulsive disorder (OCD) is a highly prevalent and chronic condition that is associated with substantial global disability. OCD is the key example of the 'obsessive-compulsive and related disorders', a group of conditions which are now classified together in the Diagnostic and Statistical Manual of Mental Disorders, Fifth Edition, and the International Classification of Diseases, 11th Revision, and which are often underdiagnosed and undertreated. In addition, OCD is an important example of a neuropsychiatric disorder in which rigorous research on phenomenology, psychobiology, pharmacotherapy and psychotherapy has contributed to better recognition, assessment and outcomes. Although OCD is a relatively homogenous disorder with similar symptom dimensions globally, individualized assessment of symptoms, the degree of insight, and the extent of comorbidity is needed. Several neurobiological mechanisms underlying OCD have been identified, including specific brain circuits that underpin OCD. In addition, laboratory models have demonstrated how cellular and molecular dysfunction underpins repetitive stereotyped behaviours, and the genetic architecture of OCD is increasingly understood. Effective treatments for OCD include serotonin reuptake inhibitors and cognitive-behavioural therapy, and neurosurgery for those with intractable symptoms. Integration of global mental health and translational neuroscience approaches could further advance knowledge on OCD and improve clinical outcomes.

**2.Assumptions of the 3 MR analysis method (IVW, MR-Egger, WM)**

The inverse variance weighted (IVW) method was used as the primary method due to its ability to provide accurate estimations. However, the IVW method might be affected by invalid IVs and pleiotropy, the MR-Egger method allows all variants to have pleiotropic effects and can provide a consistent estimate of the causal effect under a weaker instrument strength independent of direct effects assumption[31](https://onlinelibrary.wiley.com/doi/10.1111/cns.14430#cns14430-bib-0031); the weighted median (WM) approach can yield a consistent estimate if at least 50% of weight is derived from valid IVs.

**3.Supplementary Tables 1-10**

**Table S1. Instrumental variable information used for Mendelian randomization on the association of neuroticism clusters on psychiatric disorders.**

| **Exposure** | **SNP** | **Chr** | **EA** | **OA** | **EAF** | **Beta** | **SE** | **P** | **F** | **R^2^** |
| --- | --- | --- | --- | --- | --- | --- | --- | --- | --- | --- |
| worry | rs488359 | 1 | G | A | 0.307 | 0.014 | 0.003 | 2.61E-08 | 31.003 | 8.90E-05 |
| worry | rs61731122 | 1 | A | G | 0.029 | -0.042 | 0.007 | 2.04E-09 | 35.935 | 1.03E-04 |
| worry | rs34644694 | 1 | C | A | 0.278 | -0.016 | 0.003 | 3.40E-09 | 34.911 | 1.00E-04 |
| worry | rs2488401 | 1 | T | C | 0.201 | -0.018 | 0.003 | 3.14E-09 | 35.096 | 1.01E-04 |
| worry | rs1296171 | 1 | G | C | 0.478 | -0.014 | 0.002 | 1.28E-08 | 32.367 | 9.29E-05 |
| worry | rs78260322 | 2 | G | A | 0.107 | 0.022 | 0.004 | 2.21E-08 | 31.302 | 8.99E-05 |
| worry | rs2672852 | 2 | C | T | 0.488 | -0.014 | 0.002 | 8.83E-09 | 33.079 | 9.50E-05 |
| worry | rs998884 | 2 | G | A | 0.403 | 0.015 | 0.002 | 2.88E-10 | 39.773 | 1.14E-04 |
| worry | rs7567451 | 2 | G | T | 0.269 | -0.016 | 0.003 | 3.47E-09 | 34.919 | 1.00E-04 |
| worry | rs79827531 | 2 | A | G | 0.198 | 0.018 | 0.003 | 3.17E-09 | 35.092 | 1.01E-04 |
| worry | rs17196295 | 2 | G | A | 0.298 | 0.016 | 0.003 | 3.05E-09 | 35.132 | 1.01E-04 |
| worry | rs4684833 | 3 | C | T | 0.221 | 0.016 | 0.003 | 4.97E-08 | 29.742 | 8.54E-05 |
| worry | rs45536634 | 3 | A | G | 0.113 | 0.024 | 0.004 | 1.10E-10 | 41.610 | 1.19E-04 |
| worry | rs353547 | 3 | T | C | 0.377 | -0.017 | 0.002 | 4.81E-12 | 47.743 | 1.37E-04 |
| worry | rs4405857 | 3 | G | C | 0.399 | -0.013 | 0.002 | 4.72E-08 | 29.834 | 8.56E-05 |
| worry | rs6807666 | 3 | A | C | 0.352 | -0.023 | 0.002 | 1.27E-19 | 82.161 | 2.36E-04 |
| worry | rs480330 | 3 | T | C | 0.347 | -0.017 | 0.003 | 1.41E-11 | 45.639 | 1.31E-04 |
| worry | rs1724725 | 3 | A | G | 0.066 | -0.027 | 0.005 | 1.02E-08 | 32.803 | 9.42E-05 |
| worry | rs13324323 | 3 | T | C | 0.214 | -0.017 | 0.003 | 2.36E-09 | 35.663 | 1.02E-04 |
| worry | rs10005233 | 4 | C | T | 0.486 | -0.015 | 0.002 | 2.63E-10 | 39.945 | 1.15E-04 |
| worry | rs10034259 | 4 | C | A | 0.199 | -0.019 | 0.003 | 4.32E-10 | 38.958 | 1.12E-04 |
| worry | rs57360718 | 4 | C | T | 0.064 | 0.028 | 0.005 | 6.69E-09 | 33.627 | 9.65E-05 |
| worry | rs13328187 | 5 | C | T | 0.259 | 0.017 | 0.003 | 3.60E-10 | 39.338 | 1.13E-04 |
| worry | rs502652 | 5 | T | C | 0.373 | -0.014 | 0.002 | 3.64E-08 | 30.330 | 8.71E-05 |
| worry | rs391236 | 5 | G | A | 0.315 | -0.015 | 0.003 | 8.99E-09 | 33.044 | 9.49E-05 |
| worry | rs2269426 | 6 | A | G | 0.363 | 0.019 | 0.002 | 4.31E-14 | 57.009 | 1.64E-04 |
| worry | rs1593304 | 7 | A | G | 0.203 | -0.017 | 0.003 | 2.05E-08 | 31.469 | 9.03E-05 |
| worry | rs2407746 | 8 | G | C | 0.303 | 0.017 | 0.003 | 1.22E-10 | 41.429 | 1.19E-04 |
| worry | rs34354815 | 8 | C | G | 0.132 | 0.020 | 0.004 | 1.11E-08 | 32.653 | 9.37E-05 |
| worry | rs13262595 | 8 | A | G | 0.439 | 0.017 | 0.002 | 3.23E-13 | 53.069 | 1.52E-04 |
| worry | rs62551581 | 9 | C | A | 0.219 | -0.016 | 0.003 | 2.23E-08 | 31.281 | 8.98E-05 |
| worry | rs7033345 | 9 | C | T | 0.272 | -0.015 | 0.003 | 7.71E-09 | 33.345 | 9.57E-05 |
| worry | rs17532098 | 9 | T | C | 0.105 | 0.023 | 0.004 | 2.64E-09 | 35.417 | 1.02E-04 |
| worry | rs75614054 | 9 | T | C | 0.088 | 0.024 | 0.004 | 1.07E-08 | 32.707 | 9.39E-05 |
| worry | rs6478623 | 9 | G | T | 0.272 | 0.015 | 0.003 | 1.12E-08 | 32.635 | 9.37E-05 |
| worry | rs1890184 | 10 | C | A | 0.391 | -0.018 | 0.002 | 2.48E-13 | 53.562 | 1.54E-04 |
| worry | rs1826787 | 10 | T | C | 0.111 | -0.023 | 0.004 | 2.34E-09 | 35.664 | 1.02E-04 |
| worry | rs56133711 | 11 | A | G | 0.259 | -0.015 | 0.003 | 2.52E-08 | 31.060 | 8.92E-05 |
| worry | rs3026401 | 11 | C | T | 0.209 | 0.019 | 0.003 | 4.31E-11 | 43.471 | 1.25E-04 |
| worry | rs10501320 | 11 | C | G | 0.265 | 0.018 | 0.003 | 3.16E-11 | 44.066 | 1.27E-04 |
| worry | rs4245150 | 11 | G | T | 0.381 | -0.015 | 0.002 | 1.17E-09 | 36.990 | 1.06E-04 |
| worry | rs3742020 | 12 | C | T | 0.315 | 0.016 | 0.003 | 2.42E-10 | 40.078 | 1.15E-04 |
| worry | rs1330745 | 13 | C | A | 0.216 | -0.018 | 0.003 | 3.20E-10 | 39.534 | 1.13E-04 |
| worry | rs7152906 | 14 | T | C | 0.480 | -0.018 | 0.002 | 1.18E-13 | 55.043 | 1.58E-04 |
| worry | rs55997507 | 16 | G | C | 0.389 | -0.016 | 0.002 | 3.12E-10 | 39.594 | 1.14E-04 |
| worry | rs3751855 | 16 | C | T | 0.383 | -0.014 | 0.002 | 1.28E-08 | 32.367 | 9.29E-05 |
| worry | rs9936170 | 16 | G | A | 0.188 | 0.018 | 0.003 | 4.61E-09 | 34.351 | 9.86E-05 |
| worry | rs11204421 | 17 | C | T | 0.428 | 0.013 | 0.002 | 4.04E-08 | 30.115 | 8.65E-05 |
| worry | rs60642411 | 17 | A | G | 0.138 | 0.019 | 0.003 | 4.83E-08 | 29.792 | 8.55E-05 |
| worry | rs7207400 | 17 | C | T | 0.271 | 0.021 | 0.003 | 2.38E-15 | 62.703 | 1.80E-04 |
| worry | rs75195552 | 17 | A | G | 0.223 | 0.017 | 0.003 | 6.52E-09 | 33.689 | 9.67E-05 |
| worry | rs56084168 | 17 | T | C | 0.147 | -0.021 | 0.003 | 2.21E-10 | 40.293 | 1.16E-04 |
| worry | rs4799723 | 18 | T | G | 0.494 | -0.013 | 0.002 | 2.32E-08 | 31.215 | 8.96E-05 |
| worry | rs62081501 | 18 | A | G | 0.082 | 0.030 | 0.004 | 7.30E-12 | 46.948 | 1.35E-04 |
| worry | rs10871777 | 18 | G | A | 0.236 | -0.020 | 0.003 | 4.89E-13 | 52.252 | 1.50E-04 |
| worry | rs77432625 | 19 | G | C | 0.040 | 0.037 | 0.006 | 1.52E-09 | 36.503 | 1.05E-04 |
| worry | rs112591851 | 19 | T | C | 0.031 | -0.038 | 0.007 | 4.47E-08 | 29.933 | 8.59E-05 |
| worry | rs1998122 | 20 | A | G | 0.383 | -0.014 | 0.002 | 1.03E-08 | 32.793 | 9.41E-05 |
| worry | rs11090045 | 22 | A | G | 0.303 | 0.018 | 0.003 | 1.34E-11 | 45.766 | 1.31E-04 |
| SESA | rs11263943 | 1 | A | G | 0.420 | 0.016 | 0.002 | 8.23E-12 | 46.711 | 1.33E-04 |
| SESA | rs34657012 | 2 | A | C | 0.283 | 0.015 | 0.003 | 3.30E-09 | 35.015 | 9.95E-05 |
| SESA | rs17049509 | 2 | T | C | 0.294 | -0.015 | 0.003 | 1.36E-08 | 32.247 | 9.16E-05 |
| SESA | rs2627019 | 2 | T | C | 0.435 | 0.013 | 0.002 | 4.07E-08 | 30.098 | 8.55E-05 |
| SESA | rs7567451 | 2 | G | T | 0.269 | -0.014 | 0.003 | 4.54E-08 | 29.933 | 8.51E-05 |
| SESA | rs1978573 | 2 | T | C | 0.298 | -0.015 | 0.003 | 1.31E-08 | 32.309 | 9.18E-05 |
| SESA | rs9811585 | 3 | G | T | 0.381 | 0.014 | 0.002 | 3.69E-09 | 34.792 | 9.89E-05 |
| SESA | rs13070517 | 3 | T | G | 0.395 | -0.014 | 0.002 | 1.74E-08 | 31.754 | 9.02E-05 |
| SESA | rs3772882 | 3 | A | C | 0.373 | -0.015 | 0.002 | 1.18E-09 | 37.014 | 1.05E-04 |
| SESA | rs1282545 | 3 | C | T | 0.427 | 0.014 | 0.002 | 6.12E-09 | 33.796 | 9.61E-05 |
| SESA | rs6439649 | 3 | G | T | 0.399 | -0.014 | 0.002 | 1.67E-09 | 36.355 | 1.03E-04 |
| SESA | rs11167957 | 5 | C | T | 0.383 | -0.013 | 0.002 | 4.13E-08 | 30.082 | 8.55E-05 |
| SESA | rs10476484 | 5 | G | A | 0.268 | 0.015 | 0.003 | 1.98E-08 | 31.496 | 8.95E-05 |
| SESA | rs9462364 | 6 | A | G | 0.495 | -0.013 | 0.002 | 8.59E-09 | 33.129 | 9.42E-05 |
| SESA | rs9366697 | 6 | C | T | 0.356 | -0.014 | 0.002 | 2.07E-08 | 31.410 | 8.93E-05 |
| SESA | rs6941112 | 6 | A | G | 0.292 | 0.018 | 0.003 | 6.09E-12 | 47.313 | 1.34E-04 |
| SESA | rs314289 | 6 | C | T | 0.439 | -0.013 | 0.002 | 3.63E-08 | 30.362 | 8.63E-05 |
| SESA | rs62442206 | 7 | C | A | 0.133 | -0.021 | 0.003 | 8.87E-10 | 37.566 | 1.07E-04 |
| SESA | rs34351088 | 7 | C | T | 0.487 | 0.019 | 0.002 | 6.22E-17 | 69.875 | 1.99E-04 |
| SESA | rs2407746 | 8 | G | C | 0.303 | 0.016 | 0.003 | 3.47E-10 | 39.400 | 1.12E-04 |
| SESA | rs13284170 | 9 | A | T | 0.283 | 0.016 | 0.003 | 2.25E-10 | 40.225 | 1.14E-04 |
| SESA | rs10119773 | 9 | A | G | 0.463 | -0.016 | 0.002 | 4.10E-11 | 43.592 | 1.24E-04 |
| SESA | rs2282040 | 9 | G | A | 0.094 | 0.029 | 0.004 | 7.71E-13 | 51.359 | 1.46E-04 |
| SESA | rs391957 | 9 | T | C | 0.407 | -0.015 | 0.002 | 2.44E-10 | 40.074 | 1.14E-04 |
| SESA | rs11022762 | 11 | T | C | 0.383 | 0.017 | 0.002 | 3.83E-12 | 48.196 | 1.37E-04 |
| SESA | rs527528 | 11 | T | C | 0.330 | 0.019 | 0.002 | 6.57E-14 | 56.235 | 1.60E-04 |
| SESA | rs10765820 | 11 | A | G | 0.485 | 0.014 | 0.002 | 1.76E-09 | 36.257 | 1.03E-04 |
| SESA | rs35738585 | 11 | G | T | 0.434 | -0.019 | 0.002 | 4.94E-16 | 65.792 | 1.87E-04 |
| SESA | rs11613183 | 12 | C | T | 0.071 | 0.025 | 0.005 | 2.02E-08 | 31.475 | 8.95E-05 |
| SESA | rs11115117 | 12 | C | T | 0.128 | 0.019 | 0.003 | 2.13E-08 | 31.378 | 8.92E-05 |
| SESA | rs35393419 | 12 | T | C | 0.253 | 0.019 | 0.003 | 2.47E-12 | 49.094 | 1.40E-04 |
| SESA | rs73205551 | 12 | C | A | 0.161 | 0.019 | 0.003 | 1.57E-09 | 36.447 | 1.04E-04 |
| SESA | rs3124426 | 13 | C | T | 0.219 | 0.016 | 0.003 | 1.97E-08 | 31.518 | 8.96E-05 |
| SESA | rs11627348 | 14 | A | C | 0.149 | 0.019 | 0.003 | 1.05E-08 | 32.757 | 9.31E-05 |
| SESA | rs761898 | 14 | A | G | 0.377 | -0.014 | 0.002 | 2.48E-08 | 31.082 | 8.83E-05 |
| SESA | rs2305411 | 15 | C | T | 0.267 | -0.015 | 0.003 | 4.04E-09 | 34.584 | 9.83E-05 |
| SESA | rs3785237 | 16 | G | C | 0.490 | -0.016 | 0.002 | 1.05E-11 | 46.205 | 1.31E-04 |
| SESA | rs8045843 | 16 | C | G | 0.223 | 0.016 | 0.003 | 9.24E-09 | 32.998 | 9.38E-05 |
| SESA | rs4791331 | 17 | T | C | 0.463 | -0.014 | 0.002 | 3.32E-09 | 34.986 | 9.94E-05 |
| SESA | rs80143279 | 17 | C | T | 0.224 | 0.027 | 0.003 | 8.06E-22 | 92.144 | 2.62E-04 |
| SESA | rs56084168 | 17 | T | C | 0.147 | -0.020 | 0.003 | 2.03E-09 | 35.934 | 1.02E-04 |
| SESA | rs11665070 | 18 | G | A | 0.332 | 0.021 | 0.002 | 4.52E-18 | 75.088 | 2.13E-04 |
| SESA | rs11090045 | 22 | A | G | 0.303 | 0.015 | 0.003 | 5.57E-09 | 33.986 | 9.66E-05 |
| depressed affect | rs3795310 | 1 | T | C | 0.452 | 0.014 | 0.002 | 3.24E-09 | 35.026 | 9.78E-05 |
| depressed affect | rs11209175 | 1 | C | T | 0.365 | 0.015 | 0.002 | 1.65E-09 | 36.349 | 1.02E-04 |
| depressed affect | rs12137936 | 1 | G | C | 0.314 | 0.014 | 0.002 | 2.94E-08 | 30.735 | 8.59E-05 |
| depressed affect | rs12030991 | 1 | G | C | 0.239 | -0.015 | 0.003 | 3.05E-08 | 30.673 | 8.57E-05 |
| depressed affect | rs75650221 | 1 | T | C | 0.038 | -0.036 | 0.006 | 3.08E-09 | 35.137 | 9.82E-05 |
| depressed affect | rs17432675 | 1 | C | T | 0.327 | -0.014 | 0.003 | 4.04E-08 | 30.114 | 8.41E-05 |
| depressed affect | rs34668726 | 2 | G | C | 0.167 | 0.021 | 0.003 | 1.15E-11 | 46.076 | 1.29E-04 |
| depressed affect | rs2717043 | 2 | C | T | 0.377 | -0.015 | 0.002 | 1.64E-10 | 40.874 | 1.14E-04 |
| depressed affect | rs62172117 | 2 | A | G | 0.357 | -0.014 | 0.002 | 6.19E-09 | 33.791 | 9.44E-05 |
| depressed affect | rs2042555 | 2 | A | G | 0.415 | 0.014 | 0.002 | 9.45E-10 | 37.421 | 1.05E-04 |
| depressed affect | rs76923064 | 2 | C | T | 0.013 | 0.056 | 0.010 | 3.24E-08 | 30.569 | 8.54E-05 |
| depressed affect | rs11693031 | 2 | G | A | 0.321 | -0.016 | 0.003 | 4.59E-10 | 38.840 | 1.08E-04 |
| depressed affect | rs9858071 | 3 | C | T | 0.288 | 0.016 | 0.003 | 3.25E-10 | 39.528 | 1.10E-04 |
| depressed affect | rs1542212 | 3 | G | T | 0.392 | 0.013 | 0.002 | 2.31E-08 | 31.202 | 8.72E-05 |
| depressed affect | rs4625 | 3 | G | A | 0.307 | -0.016 | 0.003 | 4.21E-10 | 39.044 | 1.09E-04 |
| depressed affect | rs9852417 | 3 | C | A | 0.410 | 0.013 | 0.002 | 2.84E-08 | 30.786 | 8.60E-05 |
| depressed affect | rs836927 | 3 | A | C | 0.427 | 0.015 | 0.002 | 4.11E-10 | 39.076 | 1.09E-04 |
| depressed affect | rs59382200 | 3 | G | A | 0.401 | -0.016 | 0.002 | 2.44E-11 | 44.576 | 1.25E-04 |
| depressed affect | rs6795372 | 3 | G | T | 0.371 | 0.015 | 0.002 | 1.40E-09 | 36.716 | 1.03E-04 |
| depressed affect | rs10020288 | 4 | A | G | 0.360 | -0.013 | 0.002 | 2.56E-08 | 31.038 | 8.67E-05 |
| depressed affect | rs13122395 | 4 | A | G | 0.326 | 0.014 | 0.002 | 3.63E-08 | 30.360 | 8.48E-05 |
| depressed affect | rs77087420 | 4 | G | A | 0.055 | -0.033 | 0.005 | 1.06E-10 | 41.708 | 1.17E-04 |
| depressed affect | rs6818081 | 4 | T | C | 0.148 | 0.018 | 0.003 | 2.83E-08 | 30.826 | 8.61E-05 |
| depressed affect | rs55965054 | 5 | C | T | 0.441 | 0.014 | 0.002 | 2.67E-09 | 35.427 | 9.90E-05 |
| depressed affect | rs1422192 | 5 | A | G | 0.158 | 0.019 | 0.003 | 1.44E-09 | 36.616 | 1.02E-04 |
| depressed affect | rs7714426 | 5 | C | G | 0.151 | 0.018 | 0.003 | 1.41E-08 | 32.184 | 8.99E-05 |
| depressed affect | rs28893517 | 6 | G | A | 0.068 | 0.028 | 0.005 | 5.16E-10 | 38.617 | 1.08E-04 |
| depressed affect | rs6900114 | 6 | A | G | 0.265 | -0.015 | 0.003 | 2.30E-08 | 31.200 | 8.72E-05 |
| depressed affect | rs11514731 | 7 | G | C | 0.181 | -0.017 | 0.003 | 3.50E-08 | 30.406 | 8.49E-05 |
| depressed affect | rs10950393 | 7 | C | T | 0.417 | 0.016 | 0.002 | 2.37E-11 | 44.654 | 1.25E-04 |
| depressed affect | rs2396133 | 7 | G | A | 0.476 | 0.013 | 0.002 | 1.18E-08 | 32.527 | 9.09E-05 |
| depressed affect | rs7827176 | 8 | T | A | 0.422 | -0.013 | 0.002 | 1.87E-08 | 31.602 | 8.83E-05 |
| depressed affect | rs10156548 | 9 | G | C | 0.358 | 0.018 | 0.002 | 2.55E-13 | 53.548 | 1.50E-04 |
| depressed affect | rs2895249 | 9 | A | G | 0.391 | -0.013 | 0.002 | 2.34E-08 | 31.202 | 8.72E-05 |
| depressed affect | rs2149351 | 9 | T | G | 0.242 | 0.016 | 0.003 | 2.27E-09 | 35.708 | 9.97E-05 |
| depressed affect | rs60393230 | 9 | A | G | 0.404 | -0.014 | 0.002 | 3.57E-09 | 34.863 | 9.74E-05 |
| depressed affect | rs11599236 | 10 | C | T | 0.410 | -0.016 | 0.002 | 3.31E-11 | 43.981 | 1.23E-04 |
| depressed affect | rs7912226 | 10 | A | T | 0.397 | -0.015 | 0.002 | 8.12E-10 | 37.703 | 1.05E-04 |
| depressed affect | rs297346 | 11 | A | G | 0.362 | 0.016 | 0.002 | 1.81E-11 | 45.185 | 1.26E-04 |
| depressed affect | rs11039149 | 11 | G | A | 0.278 | 0.019 | 0.003 | 6.01E-14 | 56.352 | 1.57E-04 |
| depressed affect | rs721496 | 11 | A | G | 0.252 | 0.016 | 0.003 | 4.60E-09 | 34.361 | 9.60E-05 |
| depressed affect | rs35738585 | 11 | G | T | 0.434 | -0.017 | 0.002 | 1.73E-13 | 54.294 | 1.52E-04 |
| depressed affect | rs11605020 | 11 | G | A | 0.486 | -0.014 | 0.002 | 3.59E-09 | 34.805 | 9.72E-05 |
| depressed affect | rs11608355 | 12 | C | T | 0.315 | 0.015 | 0.002 | 5.10E-09 | 34.129 | 9.53E-05 |
| depressed affect | rs3843954 | 13 | C | G | 0.292 | 0.014 | 0.003 | 3.82E-08 | 30.228 | 8.44E-05 |
| depressed affect | rs77607745 | 13 | C | A | 0.190 | 0.016 | 0.003 | 4.95E-08 | 29.752 | 8.31E-05 |
| depressed affect | rs1782179 | 14 | T | C | 0.273 | -0.015 | 0.003 | 1.50E-08 | 32.083 | 8.96E-05 |
| depressed affect | rs4902704 | 14 | G | C | 0.387 | -0.014 | 0.002 | 1.18E-08 | 32.536 | 9.09E-05 |
| depressed affect | rs10144845 | 14 | C | T | 0.317 | -0.016 | 0.002 | 1.05E-10 | 41.731 | 1.17E-04 |
| depressed affect | rs35755513 | 15 | T | C | 0.074 | 0.024 | 0.004 | 4.12E-08 | 30.100 | 8.41E-05 |
| depressed affect | rs7175083 | 15 | T | C | 0.478 | 0.015 | 0.002 | 2.21E-10 | 40.296 | 1.13E-04 |
| depressed affect | rs9930139 | 16 | A | C | 0.412 | -0.014 | 0.002 | 1.19E-08 | 32.522 | 9.08E-05 |
| depressed affect | rs12938775 | 17 | G | A | 0.498 | 0.013 | 0.002 | 2.49E-08 | 31.062 | 8.68E-05 |
| depressed affect | rs62057061 | 17 | G | C | 0.225 | 0.027 | 0.003 | 2.37E-22 | 94.578 | 2.64E-04 |
| depressed affect | rs7502590 | 17 | G | A | 0.148 | -0.018 | 0.003 | 3.12E-08 | 30.633 | 8.56E-05 |
| depressed affect | rs12967855 | 18 | A | G | 0.329 | 0.017 | 0.002 | 2.11E-12 | 49.362 | 1.38E-04 |
| depressed affect | rs4632195 | 18 | C | T | 0.487 | -0.016 | 0.002 | 6.63E-12 | 47.132 | 1.32E-04 |
| depressed affect | rs599550 | 18 | G | A | 0.151 | -0.027 | 0.003 | 3.69E-17 | 70.955 | 1.98E-04 |
| depressed affect | rs10405382 | 19 | G | C | 0.169 | -0.020 | 0.003 | 7.24E-11 | 42.452 | 1.19E-04 |
| depressed affect | rs4578918 | 20 | T | C | 0.261 | 0.018 | 0.003 | 1.03E-11 | 46.285 | 1.29E-04 |

Abbreviation

EA, effect allele; OA, other allele; EAF, effect allele frequency; SE, standard error; Chr, chromosome; SESA, sensitivity to environmental stress; SNP, single nucleotide polymorphism.

**Table S2. Instrumental variable information used for Mendelian randomization on the association among psychiatric disorders based on the association of neuroticism clusters on psychiatric disorders.**

| **Exposure** | **SNP** | **Chr** | **EA** | **OA** | **EAF** | **Beta** | **SE** | **P** | **F** | **R^2^** |
| --- | --- | --- | --- | --- | --- | --- | --- | --- | --- | --- |
| PD | rs77338525 | 10 | T | C | 0.027 | 0.581 | 0.116 | 5.83E-07 | 25.086 | 2.44E-03 |
| PD | rs12289248 | 11 | T | C | 0.331 | 0.190 | 0.041 | 3.42E-06 | 21.475 | 2.09E-03 |
| PD | rs1912779 | 12 | T | G | 0.095 | 0.330 | 0.068 | 1.32E-06 | 23.551 | 2.29E-03 |
| PD | rs79918695 | 1 | T | C | 0.920 | -0.338 | 0.068 | 6.83E-07 | 24.707 | 2.41E-03 |
| PD | rs112586150 | 15 | A | G | 0.036 | 0.458 | 0.098 | 3.10E-06 | 21.841 | 2.13E-03 |
| PD | rs79919349 | 20 | A | G | 0.018 | 0.816 | 0.162 | 4.71E-07 | 25.372 | 2.47E-03 |
| PD | rs55888696 | 3 | A | C | 0.017 | 0.786 | 0.157 | 5.35E-07 | 25.064 | 2.44E-03 |
| PD | rs150769867 | 4 | A | G | 0.987 | -0.797 | 0.173 | 3.94E-06 | 21.224 | 2.07E-03 |
| PD | rs144783209 | 4 | T | G | 0.028 | 0.532 | 0.111 | 1.47E-06 | 22.971 | 2.24E-03 |
| PD | rs72737735 | 5 | T | C | 0.053 | 0.463 | 0.099 | 3.23E-06 | 21.872 | 2.13E-03 |
| PD | rs142111923 | 5 | A | G | 0.011 | 0.923 | 0.184 | 5.39E-07 | 25.163 | 2.45E-03 |
| PD | rs77941126 | 6 | A | T | 0.893 | -0.304 | 0.064 | 2.16E-06 | 22.563 | 2.20E-03 |
| PD | rs41280169 | 9 | T | C | 0.050 | 0.505 | 0.101 | 6.00E-07 | 25.000 | 2.44E-03 |
| SCZ | rs11587347 | 1 | G | C | 0.094 | 0.104 | 0.015 | 1.53E-12 | 48.071 | 8.02E-04 |
| SCZ | rs56335113 | 1 | G | A | 0.697 | -0.065 | 0.009 | 6.02E-12 | 52.160 | 8.70E-04 |
| SCZ | rs12129573 | 1 | A | C | 0.366 | 0.078 | 0.009 | 2.28E-18 | 75.111 | 1.25E-03 |
| SCZ | rs145071536 | 1 | C | T | 0.192 | 0.085 | 0.012 | 1.62E-12 | 50.174 | 8.37E-04 |
| SCZ | rs4653164 | 1 | T | C | 0.668 | 0.051 | 0.009 | 3.08E-08 | 32.111 | 5.36E-04 |
| SCZ | rs1892346 | 1 | A | T | 0.564 | 0.048 | 0.009 | 3.56E-08 | 28.444 | 4.75E-04 |
| SCZ | rs1198588 | 1 | T | A | 0.788 | 0.103 | 0.011 | 1.73E-21 | 87.678 | 1.46E-03 |
| SCZ | rs12138231 | 1 | A | T | 0.817 | 0.067 | 0.012 | 7.99E-09 | 31.174 | 5.20E-04 |
| SCZ | rs7515363 | 1 | T | C | 0.619 | -0.054 | 0.009 | 1.84E-09 | 36.000 | 6.01E-04 |
| SCZ | rs6673880 | 1 | G | A | 0.495 | 0.062 | 0.009 | 7.19E-12 | 47.457 | 7.92E-04 |
| SCZ | rs3795310 | 1 | T | C | 0.463 | -0.051 | 0.009 | 5.75E-09 | 32.111 | 5.36E-04 |
| SCZ | rs11210892 | 1 | A | G | 0.670 | -0.064 | 0.009 | 2.68E-12 | 50.568 | 8.43E-04 |
| SCZ | rs11165867 | 1 | T | C | 0.160 | 0.074 | 0.012 | 1.30E-10 | 38.028 | 6.34E-04 |
| SCZ | rs16851048 | 1 | C | T | 0.195 | 0.074 | 0.011 | 4.15E-12 | 45.256 | 7.55E-04 |
| SCZ | rs1451488 | 2 | G | A | 0.555 | 0.071 | 0.009 | 4.47E-16 | 62.235 | 1.04E-03 |
| SCZ | rs2167378 | 2 | T | C | 0.439 | -0.065 | 0.009 | 7.30E-14 | 52.160 | 8.70E-04 |
| SCZ | rs3791710 | 2 | C | T | 0.203 | -0.060 | 0.011 | 3.02E-08 | 29.752 | 4.96E-04 |
| SCZ | rs6715366 | 2 | A | G | 0.267 | 0.054 | 0.010 | 2.49E-08 | 29.160 | 4.87E-04 |
| SCZ | rs6721531 | 2 | T | A | 0.341 | -0.052 | 0.009 | 1.47E-08 | 33.383 | 5.57E-04 |
| SCZ | rs13016542 | 2 | C | T | 0.136 | -0.088 | 0.013 | 8.28E-12 | 45.822 | 7.64E-04 |
| SCZ | rs3739118 | 2 | A | G | 0.291 | -0.057 | 0.010 | 2.36E-09 | 32.490 | 5.42E-04 |
| SCZ | rs12712510 | 2 | C | T | 0.527 | -0.057 | 0.009 | 5.14E-11 | 40.111 | 6.69E-04 |
| SCZ | rs7575796 | 2 | G | A | 0.094 | -0.096 | 0.017 | 2.07E-08 | 31.889 | 5.32E-04 |
| SCZ | rs1881046 | 2 | T | G | 0.340 | -0.051 | 0.009 | 3.39E-08 | 32.111 | 5.36E-04 |
| SCZ | rs62183855 | 2 | C | A | 0.192 | -0.066 | 0.011 | 2.66E-09 | 36.000 | 6.01E-04 |
| SCZ | rs11693094 | 2 | T | C | 0.461 | -0.054 | 0.009 | 4.29E-10 | 36.000 | 6.01E-04 |
| SCZ | rs12151767 | 2 | A | G | 0.487 | -0.061 | 0.009 | 1.31E-12 | 45.938 | 7.66E-04 |
| SCZ | rs778371 | 2 | G | A | 0.286 | 0.081 | 0.010 | 1.49E-17 | 65.610 | 1.09E-03 |
| SCZ | rs13011472 | 2 | G | C | 0.484 | 0.070 | 0.009 | 4.28E-16 | 60.494 | 1.01E-03 |
| SCZ | rs3770754 | 2 | G | C | 0.368 | -0.053 | 0.009 | 5.35E-09 | 34.679 | 5.79E-04 |
| SCZ | rs6546857 | 2 | G | A | 0.232 | 0.060 | 0.010 | 2.74E-09 | 36.000 | 6.01E-04 |
| SCZ | rs2909457 | 2 | A | G | 0.564 | -0.049 | 0.009 | 1.48E-08 | 29.642 | 4.95E-04 |
| SCZ | rs12489270 | 3 | C | T | 0.375 | 0.058 | 0.009 | 7.47E-11 | 41.531 | 6.93E-04 |
| SCZ | rs6798742 | 3 | G | A | 0.307 | 0.061 | 0.009 | 4.57E-11 | 45.938 | 7.66E-04 |
| SCZ | rs167924 | 3 | G | A | 0.631 | 0.050 | 0.009 | 2.34E-08 | 30.864 | 5.15E-04 |
| SCZ | rs1430894 | 3 | T | C | 0.483 | 0.053 | 0.009 | 6.15E-10 | 34.679 | 5.79E-04 |
| SCZ | rs6549963 | 3 | C | T | 0.416 | -0.048 | 0.009 | 4.31E-08 | 28.444 | 4.75E-04 |
| SCZ | rs2710323 | 3 | C | T | 0.490 | -0.078 | 0.009 | 1.23E-19 | 75.111 | 1.25E-03 |
| SCZ | rs17194490 | 3 | T | G | 0.161 | 0.078 | 0.012 | 1.80E-11 | 42.250 | 7.05E-04 |
| SCZ | rs60135207 | 3 | T | G | 0.415 | -0.050 | 0.009 | 1.53E-08 | 30.864 | 5.15E-04 |
| SCZ | rs7634476 | 3 | G | A | 0.586 | 0.058 | 0.009 | 5.46E-11 | 41.531 | 6.93E-04 |
| SCZ | rs9876421 | 3 | T | C | 0.343 | 0.063 | 0.009 | 9.19E-12 | 49.000 | 8.17E-04 |
| SCZ | rs1604060 | 3 | G | A | 0.885 | 0.077 | 0.014 | 3.24E-08 | 30.250 | 5.05E-04 |
| SCZ | rs308697 | 3 | A | C | 0.436 | -0.050 | 0.009 | 8.83E-09 | 30.864 | 5.15E-04 |
| SCZ | rs7647398 | 3 | T | C | 0.200 | -0.077 | 0.011 | 1.07E-12 | 49.000 | 8.17E-04 |
| SCZ | rs35734242 | 4 | C | T | 0.426 | 0.051 | 0.009 | 1.37E-08 | 32.111 | 5.36E-04 |
| SCZ | rs215412 | 4 | A | G | 0.328 | 0.058 | 0.009 | 2.69E-10 | 41.531 | 6.93E-04 |
| SCZ | rs11941714 | 4 | A | G | 0.336 | -0.052 | 0.009 | 3.07E-08 | 33.383 | 5.57E-04 |
| SCZ | rs1427633 | 4 | C | G | 0.593 | -0.048 | 0.009 | 4.10E-08 | 28.444 | 4.75E-04 |
| SCZ | rs13107325 | 4 | T | C | 0.067 | 0.159 | 0.017 | 2.90E-21 | 87.478 | 1.46E-03 |
| SCZ | rs2333321 | 4 | G | A | 0.792 | -0.071 | 0.011 | 1.25E-11 | 41.661 | 6.95E-04 |
| SCZ | rs10035564 | 5 | G | A | 0.331 | 0.067 | 0.009 | 4.38E-13 | 55.420 | 9.24E-04 |
| SCZ | rs16867571 | 5 | G | A | 0.235 | -0.066 | 0.010 | 2.68E-10 | 43.560 | 7.27E-04 |
| SCZ | rs10117 | 5 | A | G | 0.398 | -0.055 | 0.009 | 4.66E-10 | 37.346 | 6.23E-04 |
| SCZ | rs1901512 | 5 | C | T | 0.699 | -0.058 | 0.009 | 5.72E-10 | 41.531 | 6.93E-04 |
| SCZ | rs187557 | 5 | T | C | 0.848 | -0.067 | 0.012 | 2.03E-08 | 31.174 | 5.20E-04 |
| SCZ | rs72802868 | 5 | T | G | 0.294 | -0.069 | 0.010 | 4.55E-13 | 47.610 | 7.94E-04 |
| SCZ | rs11740474 | 5 | T | A | 0.415 | 0.054 | 0.009 | 1.13E-09 | 36.000 | 6.01E-04 |
| SCZ | rs9687282 | 5 | G | T | 0.334 | 0.053 | 0.009 | 7.33E-09 | 34.679 | 5.79E-04 |
| SCZ | rs12652777 | 5 | C | T | 0.522 | -0.049 | 0.009 | 1.52E-08 | 29.642 | 4.95E-04 |
| SCZ | rs4700418 | 5 | G | C | 0.493 | 0.070 | 0.009 | 5.37E-16 | 60.494 | 1.01E-03 |
| SCZ | rs9454727 | 6 | G | A | 0.273 | -0.054 | 0.010 | 3.35E-08 | 29.160 | 4.87E-04 |
| SCZ | rs2815731 | 6 | A | C | 0.358 | -0.060 | 0.009 | 4.39E-11 | 44.444 | 7.41E-04 |
| SCZ | rs34555420 | 6 | T | G | 0.075 | -0.169 | 0.017 | 1.54E-22 | 98.827 | 1.65E-03 |
| SCZ | rs634940 | 6 | T | G | 0.252 | 0.066 | 0.010 | 1.78E-11 | 43.560 | 7.27E-04 |
| SCZ | rs9461916 | 6 | C | T | 0.594 | 0.053 | 0.009 | 1.64E-09 | 34.679 | 5.79E-04 |
| SCZ | rs217336 | 6 | A | C | 0.434 | -0.050 | 0.009 | 8.05E-09 | 30.864 | 5.15E-04 |
| SCZ | rs13195636 | 6 | C | A | 0.091 | -0.211 | 0.016 | 6.55E-40 | 173.910 | 2.89E-03 |
| SCZ | rs1611236 | 6 | A | G | 0.314 | -0.055 | 0.010 | 8.47E-09 | 30.250 | 5.05E-04 |
| SCZ | rs7798283 | 7 | G | T | 0.133 | -0.074 | 0.013 | 3.49E-08 | 32.402 | 5.41E-04 |
| SCZ | rs728055 | 7 | A | T | 0.358 | -0.067 | 0.009 | 8.85E-14 | 55.420 | 9.24E-04 |
| SCZ | rs1914399 | 7 | G | C | 0.521 | -0.049 | 0.009 | 1.40E-08 | 29.642 | 4.95E-04 |
| SCZ | rs13233308 | 7 | T | C | 0.484 | -0.049 | 0.009 | 1.75E-08 | 29.642 | 4.95E-04 |
| SCZ | rs58120505 | 7 | C | T | 0.418 | -0.090 | 0.009 | 2.24E-24 | 100.000 | 1.67E-03 |
| SCZ | rs6943762 | 7 | C | T | 0.130 | -0.105 | 0.013 | 1.57E-15 | 65.237 | 1.09E-03 |
| SCZ | rs6974218 | 7 | C | A | 0.382 | -0.055 | 0.009 | 6.80E-10 | 37.346 | 6.23E-04 |
| SCZ | rs35426637 | 7 | T | G | 0.324 | -0.062 | 0.009 | 2.15E-11 | 47.457 | 7.92E-04 |
| SCZ | rs79210963 | 7 | C | T | 0.108 | 0.086 | 0.014 | 4.14E-10 | 37.735 | 6.30E-04 |
| SCZ | rs2252074 | 7 | G | T | 0.395 | 0.069 | 0.009 | 6.19E-15 | 58.778 | 9.80E-04 |
| SCZ | rs1593304 | 7 | G | A | 0.801 | 0.064 | 0.011 | 7.45E-09 | 33.851 | 5.65E-04 |
| SCZ | rs73229090 | 8 | A | C | 0.112 | -0.103 | 0.014 | 4.34E-13 | 54.128 | 9.03E-04 |
| SCZ | rs11136325 | 8 | A | G | 0.579 | -0.054 | 0.009 | 3.05E-09 | 36.000 | 6.01E-04 |
| SCZ | rs79445414 | 8 | C | T | 0.040 | 0.123 | 0.022 | 2.80E-08 | 31.258 | 5.22E-04 |
| SCZ | rs6984242 | 8 | A | G | 0.600 | -0.055 | 0.009 | 3.85E-10 | 37.346 | 6.23E-04 |
| SCZ | rs1915019 | 8 | G | A | 0.750 | -0.057 | 0.010 | 6.57E-09 | 32.490 | 5.42E-04 |
| SCZ | rs4129585 | 8 | C | A | 0.562 | -0.075 | 0.009 | 5.11E-18 | 69.444 | 1.16E-03 |
| SCZ | rs4921741 | 8 | G | A | 0.261 | 0.056 | 0.010 | 1.21E-08 | 31.360 | 5.23E-04 |
| SCZ | rs10957321 | 8 | A | G | 0.511 | 0.048 | 0.009 | 3.48E-08 | 28.444 | 4.75E-04 |
| SCZ | rs10103330 | 8 | A | T | 0.194 | 0.067 | 0.011 | 5.07E-10 | 37.099 | 6.19E-04 |
| SCZ | rs10086619 | 8 | G | A | 0.161 | 0.072 | 0.012 | 4.97E-10 | 36.000 | 6.01E-04 |
| SCZ | rs500102 | 9 | C | T | 0.591 | -0.052 | 0.009 | 4.87E-09 | 33.383 | 5.57E-04 |
| SCZ | rs2381411 | 9 | C | T | 0.402 | 0.050 | 0.009 | 1.25E-08 | 30.864 | 5.15E-04 |
| SCZ | rs498591 | 9 | T | A | 0.146 | 0.072 | 0.012 | 2.11E-09 | 36.000 | 6.01E-04 |
| SCZ | rs2078266 | 9 | G | A | 0.829 | -0.070 | 0.013 | 2.94E-08 | 28.994 | 4.84E-04 |
| SCZ | rs505061 | 9 | A | C | 0.488 | 0.053 | 0.009 | 5.80E-10 | 34.679 | 5.79E-04 |
| SCZ | rs3824451 | 9 | C | T | 0.153 | 0.066 | 0.012 | 2.54E-08 | 30.250 | 5.05E-04 |
| SCZ | rs6482437 | 10 | C | A | 0.888 | 0.099 | 0.014 | 3.33E-12 | 50.005 | 8.34E-04 |
| SCZ | rs61857878 | 10 | T | A | 0.254 | -0.060 | 0.010 | 4.44E-09 | 36.000 | 6.01E-04 |
| SCZ | rs17731 | 10 | A | G | 0.365 | 0.052 | 0.009 | 4.37E-09 | 33.383 | 5.57E-04 |
| SCZ | rs12771371 | 10 | A | G | 0.311 | -0.052 | 0.009 | 1.94E-08 | 33.383 | 5.57E-04 |
| SCZ | rs11191580 | 10 | C | T | 0.090 | -0.132 | 0.016 | 1.77E-17 | 68.063 | 1.13E-03 |
| SCZ | rs2514218 | 11 | T | C | 0.344 | -0.070 | 0.009 | 1.35E-14 | 60.494 | 1.01E-03 |
| SCZ | rs72943392 | 11 | C | G | 0.289 | 0.053 | 0.010 | 2.39E-08 | 28.090 | 4.69E-04 |
| SCZ | rs7113199 | 11 | C | A | 0.704 | -0.052 | 0.009 | 2.80E-08 | 33.383 | 5.57E-04 |
| SCZ | rs708228 | 11 | T | C | 0.329 | 0.053 | 0.009 | 6.56E-09 | 34.679 | 5.79E-04 |
| SCZ | rs7112616 | 11 | C | T | 0.498 | -0.052 | 0.009 | 1.52E-09 | 33.383 | 5.57E-04 |
| SCZ | rs3802924 | 11 | C | A | 0.212 | -0.074 | 0.011 | 9.58E-12 | 45.256 | 7.55E-04 |
| SCZ | rs11027839 | 11 | C | A | 0.500 | 0.052 | 0.009 | 2.40E-09 | 33.383 | 5.57E-04 |
| SCZ | rs4636654 | 11 | A | G | 0.402 | -0.048 | 0.009 | 4.89E-08 | 28.444 | 4.75E-04 |
| SCZ | rs12285419 | 11 | A | C | 0.188 | 0.085 | 0.011 | 1.05E-14 | 59.711 | 9.96E-04 |
| SCZ | rs12293670 | 11 | G | A | 0.340 | -0.070 | 0.009 | 1.56E-14 | 60.494 | 1.01E-03 |
| SCZ | rs1860002 | 12 | T | C | 0.543 | -0.084 | 0.009 | 1.04E-21 | 87.111 | 1.45E-03 |
| SCZ | rs10861176 | 12 | A | G | 0.737 | 0.056 | 0.010 | 1.59E-08 | 31.360 | 5.23E-04 |
| SCZ | rs4766428 | 12 | T | C | 0.441 | 0.075 | 0.009 | 3.93E-17 | 69.444 | 1.16E-03 |
| SCZ | rs12303743 | 12 | C | G | 0.094 | 0.087 | 0.015 | 1.59E-09 | 33.640 | 5.61E-04 |
| SCZ | rs1615350 | 12 | T | C | 0.744 | -0.074 | 0.010 | 4.92E-14 | 54.760 | 9.13E-04 |
| SCZ | rs12833624 | 12 | T | C | 0.342 | 0.050 | 0.009 | 2.77E-08 | 30.864 | 5.15E-04 |
| SCZ | rs10876446 | 12 | C | G | 0.312 | 0.054 | 0.009 | 1.03E-08 | 36.000 | 6.01E-04 |
| SCZ | rs61937595 | 12 | T | C | 0.092 | -0.130 | 0.016 | 1.15E-15 | 66.016 | 1.10E-03 |
| SCZ | rs6538539 | 12 | T | G | 0.549 | -0.057 | 0.009 | 4.43E-11 | 40.111 | 6.69E-04 |
| SCZ | rs2455415 | 13 | T | C | 0.413 | 0.049 | 0.009 | 1.69E-08 | 29.642 | 4.95E-04 |
| SCZ | rs12877581 | 13 | C | G | 0.277 | 0.060 | 0.010 | 1.80E-09 | 36.000 | 6.01E-04 |
| SCZ | rs9318627 | 13 | C | A | 0.400 | -0.061 | 0.009 | 4.35E-12 | 45.938 | 7.66E-04 |
| SCZ | rs2332700 | 14 | G | C | 0.753 | -0.075 | 0.010 | 3.88E-14 | 56.250 | 9.38E-04 |
| SCZ | rs10873538 | 14 | G | T | 0.334 | 0.067 | 0.009 | 3.01E-13 | 55.420 | 9.24E-04 |
| SCZ | rs1953205 | 14 | A | T | 0.485 | 0.050 | 0.009 | 2.21E-08 | 30.864 | 5.15E-04 |
| SCZ | rs12883788 | 14 | T | C | 0.458 | 0.061 | 0.009 | 1.86E-12 | 45.938 | 7.66E-04 |
| SCZ | rs2999392 | 14 | T | C | 0.691 | 0.052 | 0.009 | 3.05E-08 | 33.383 | 5.57E-04 |
| SCZ | rs1540840 | 14 | C | G | 0.475 | -0.056 | 0.009 | 2.21E-09 | 38.716 | 6.46E-04 |
| SCZ | rs56205728 | 15 | A | G | 0.287 | 0.063 | 0.010 | 1.01E-10 | 39.690 | 6.62E-04 |
| SCZ | rs4779050 | 15 | G | T | 0.633 | -0.058 | 0.009 | 7.27E-11 | 41.531 | 6.93E-04 |
| SCZ | rs62018952 | 15 | C | T | 0.726 | 0.058 | 0.010 | 1.94E-09 | 33.640 | 5.61E-04 |
| SCZ | rs35351411 | 15 | C | A | 0.544 | 0.064 | 0.009 | 2.21E-13 | 50.568 | 8.43E-04 |
| SCZ | rs2456020 | 15 | T | C | 0.241 | -0.082 | 0.010 | 1.13E-15 | 67.240 | 1.12E-03 |
| SCZ | rs4702 | 15 | A | G | 0.560 | -0.084 | 0.009 | 2.79E-21 | 87.111 | 1.45E-03 |
| SCZ | rs3814883 | 16 | T | C | 0.467 | -0.067 | 0.009 | 1.58E-14 | 55.420 | 9.24E-04 |
| SCZ | rs149165 | 16 | G | T | 0.446 | -0.048 | 0.009 | 3.00E-08 | 28.444 | 4.75E-04 |
| SCZ | rs4575535 | 16 | G | A | 0.704 | 0.056 | 0.010 | 5.77E-09 | 31.360 | 5.23E-04 |
| SCZ | rs8055219 | 16 | A | G | 0.229 | 0.067 | 0.010 | 5.69E-11 | 44.890 | 7.49E-04 |
| SCZ | rs73292401 | 17 | A | T | 0.192 | 0.068 | 0.011 | 5.48E-10 | 38.215 | 6.38E-04 |
| SCZ | rs57433322 | 17 | G | C | 0.122 | -0.083 | 0.014 | 1.99E-09 | 35.148 | 5.86E-04 |
| SCZ | rs2696466 | 17 | G | A | 0.403 | -0.061 | 0.009 | 2.64E-11 | 45.938 | 7.66E-04 |
| SCZ | rs11664298 | 18 | A | G | 0.193 | 0.077 | 0.011 | 8.94E-13 | 49.000 | 8.17E-04 |
| SCZ | rs9304548 | 18 | A | C | 0.748 | -0.057 | 0.010 | 1.59E-08 | 32.490 | 5.42E-04 |
| SCZ | rs4632195 | 18 | T | C | 0.520 | 0.047 | 0.009 | 4.59E-08 | 27.272 | 4.55E-04 |
| SCZ | rs9636107 | 18 | G | A | 0.471 | 0.070 | 0.009 | 5.11E-16 | 60.494 | 1.01E-03 |
| SCZ | rs72986630 | 19 | T | C | 0.068 | 0.112 | 0.018 | 3.59E-10 | 38.716 | 6.46E-04 |
| SCZ | rs7251 | 19 | G | C | 0.328 | -0.064 | 0.009 | 8.29E-12 | 50.568 | 8.43E-04 |
| SCZ | rs1000237 | 19 | A | T | 0.355 | 0.073 | 0.009 | 2.80E-16 | 65.790 | 1.10E-03 |
| SCZ | rs2053079 | 19 | G | A | 0.237 | 0.060 | 0.010 | 3.01E-09 | 36.000 | 6.01E-04 |
| SCZ | rs4812325 | 20 | A | G | 0.616 | 0.072 | 0.009 | 8.96E-16 | 64.000 | 1.07E-03 |
| SCZ | rs11696755 | 20 | C | T | 0.184 | 0.064 | 0.011 | 7.26E-09 | 33.851 | 5.65E-04 |
| SCZ | rs113264400 | 20 | C | T | 0.048 | 0.112 | 0.020 | 2.86E-08 | 31.360 | 5.23E-04 |
| SCZ | rs6001259 | 22 | T | C | 0.016 | 0.192 | 0.035 | 3.70E-08 | 30.093 | 5.02E-04 |
| SCZ | rs132582 | 22 | T | C | 0.545 | -0.051 | 0.009 | 3.26E-09 | 32.111 | 5.36E-04 |
| SCZ | rs5751191 | 22 | C | T | 0.501 | 0.066 | 0.009 | 3.00E-14 | 53.778 | 8.97E-04 |
| SCZ | rs8138941 | 22 | A | G | 0.204 | 0.058 | 0.011 | 4.46E-08 | 27.802 | 4.64E-04 |
| SCZ | rs6010045 | 22 | C | T | 0.698 | 0.055 | 0.010 | 7.44E-09 | 30.250 | 5.05E-04 |
| MDD | rs159963 | 1 | A | C | NA | -0.027 | 0.005 | 3.19E-08 | 29.160 | 1.69E-04 |
| MDD | rs1432639 | 1 | A | C | NA | 0.039 | 0.005 | 4.55E-15 | 60.840 | 3.52E-04 |
| MDD | rs2389016 | 1 | T | C | NA | 0.031 | 0.005 | 1.02E-08 | 38.440 | 2.22E-04 |
| MDD | rs9427672 | 1 | G | A | NA | 0.032 | 0.006 | 3.12E-08 | 28.444 | 1.64E-04 |
| MDD | rs1226412 | 2 | T | C | NA | 0.033 | 0.006 | 2.38E-08 | 30.250 | 1.75E-04 |
| MDD | rs76485002 | 2 | G | A | NA | -0.109 | 0.018 | 1.60E-09 | 36.670 | 2.12E-04 |
| MDD | rs11682175 | 2 | C | T | NA | 0.028 | 0.005 | 4.68E-09 | 31.360 | 1.81E-04 |
| MDD | rs7430565 | 3 | A | G | NA | -0.029 | 0.005 | 2.87E-09 | 33.640 | 1.94E-04 |
| MDD | rs34215985 | 4 | G | C | NA | 0.037 | 0.006 | 3.13E-09 | 38.028 | 2.20E-04 |
| MDD | rs11135349 | 5 | C | A | NA | 0.029 | 0.005 | 1.09E-09 | 33.640 | 1.94E-04 |
| MDD | rs247910 | 5 | G | A | NA | 0.032 | 0.005 | 1.07E-10 | 40.960 | 2.37E-04 |
| MDD | rs1363104 | 5 | G | C | NA | -0.031 | 0.005 | 7.38E-11 | 38.440 | 2.22E-04 |
| MDD | rs9402472 | 6 | A | G | NA | 0.033 | 0.006 | 2.78E-08 | 30.250 | 1.75E-04 |
| MDD | rs6905391 | 6 | A | G | NA | -0.044 | 0.007 | 1.35E-10 | 39.510 | 2.28E-04 |
| MDD | rs10950398 | 7 | A | G | NA | 0.027 | 0.005 | 2.55E-08 | 29.160 | 1.69E-04 |
| MDD | rs12666117 | 7 | A | G | NA | 0.027 | 0.005 | 1.35E-08 | 29.160 | 1.69E-04 |
| MDD | rs1354115 | 9 | A | C | NA | 0.028 | 0.005 | 2.37E-08 | 31.360 | 1.81E-04 |
| MDD | rs10959913 | 9 | G | T | NA | -0.033 | 0.006 | 5.06E-09 | 30.250 | 1.75E-04 |
| MDD | rs7856424 | 9 | T | C | NA | -0.031 | 0.005 | 8.48E-09 | 38.440 | 2.22E-04 |
| MDD | rs61867293 | 10 | T | C | NA | -0.037 | 0.006 | 6.97E-10 | 38.028 | 2.20E-04 |
| MDD | rs1806153 | 11 | T | G | NA | 0.036 | 0.006 | 1.18E-09 | 36.000 | 2.08E-04 |
| MDD | rs4074723 | 12 | C | A | NA | 0.027 | 0.005 | 3.12E-08 | 29.160 | 1.69E-04 |
| MDD | rs12552 | 13 | G | A | NA | -0.043 | 0.005 | 6.07E-19 | 73.960 | 4.27E-04 |
| MDD | rs10149470 | 14 | G | A | NA | 0.029 | 0.005 | 3.05E-09 | 33.640 | 1.94E-04 |
| MDD | rs4904738 | 14 | C | T | NA | 0.029 | 0.005 | 2.57E-09 | 33.640 | 1.94E-04 |
| MDD | rs915057 | 14 | G | A | NA | 0.030 | 0.005 | 7.61E-10 | 36.000 | 2.08E-04 |
| MDD | rs2005864 | 14 | T | C | NA | 0.028 | 0.005 | 6.73E-09 | 31.360 | 1.81E-04 |
| MDD | rs8025231 | 15 | C | A | NA | 0.034 | 0.005 | 2.36E-12 | 46.240 | 2.67E-04 |
| MDD | rs7198928 | 16 | C | T | NA | -0.028 | 0.005 | 1.00E-08 | 31.360 | 1.81E-04 |
| MDD | rs11643192 | 16 | A | C | NA | 0.027 | 0.005 | 3.36E-08 | 29.160 | 1.69E-04 |
| MDD | rs8063603 | 16 | A | G | NA | -0.031 | 0.005 | 6.86E-09 | 38.440 | 2.22E-04 |
| MDD | rs17727765 | 17 | C | T | NA | 0.051 | 0.009 | 8.51E-09 | 32.111 | 1.86E-04 |
| MDD | rs12958048 | 18 | G | A | NA | -0.034 | 0.005 | 3.61E-11 | 46.240 | 2.67E-04 |
| MDD | rs11663393 | 18 | A | G | NA | 0.028 | 0.005 | 1.65E-08 | 31.360 | 1.81E-04 |
| MDD | rs62099069 | 18 | T | A | NA | 0.028 | 0.005 | 1.31E-08 | 31.360 | 1.81E-04 |
| MDD | rs5758265 | 22 | A | G | NA | 0.031 | 0.005 | 7.55E-09 | 38.440 | 2.22E-04 |
| AN | rs9821797 | 3 | T | A | NA | -0.157 | 0.020 | 6.99E-15 | 61.623 | 8.49E-04 |
| AN | rs6589488 | 11 | A | T | NA | 0.127 | 0.020 | 6.31E-11 | 40.323 | 5.56E-04 |
| AN | rs2287348 | 2 | C | T | NA | -0.104 | 0.018 | 5.62E-09 | 33.383 | 4.60E-04 |
| AN | rs2008387 | 10 | G | A | NA | -0.082 | 0.015 | 1.73E-08 | 29.884 | 4.12E-04 |
| AN | rs9874207 | 3 | T | C | NA | -0.081 | 0.015 | 2.05E-08 | 29.160 | 4.02E-04 |
| AN | rs10747478 | 1 | T | G | NA | 0.076 | 0.014 | 3.13E-08 | 29.469 | 4.06E-04 |
| AN | rs370838138 | 5 | G | C | NA | 0.075 | 0.014 | 3.17E-08 | 28.699 | 3.96E-04 |
| AN | rs13100344 | 3 | T | A | NA | 0.075 | 0.014 | 4.21E-08 | 28.699 | 3.96E-04 |
| AN | rs13125932 | 4 | C | T | NA | -0.072 | 0.013 | 5.85E-08 | 30.675 | 4.23E-04 |
| AN | rs750350 | 11 | G | T | NA | -0.111 | 0.021 | 9.42E-08 | 27.939 | 3.85E-04 |
| AN | rs8070063 | 17 | G | T | NA | -0.075 | 0.014 | 9.73E-08 | 28.699 | 3.96E-04 |
| AN | rs1539725 | 1 | T | C | NA | -0.073 | 0.014 | 1.26E-07 | 27.189 | 3.75E-04 |
| AN | rs11615526 | 12 | G | A | NA | 0.090 | 0.017 | 1.52E-07 | 28.028 | 3.86E-04 |
| AN | rs28380 | 22 | G | C | NA | -0.075 | 0.014 | 1.80E-07 | 28.699 | 3.96E-04 |
| AN | rs6789500 | 3 | T | C | NA | 0.091 | 0.018 | 2.55E-07 | 25.559 | 3.52E-04 |
| AN | rs2131959 | 10 | G | C | NA | 0.077 | 0.015 | 3.04E-07 | 26.351 | 3.63E-04 |
| AN | rs6092932 | 20 | G | A | NA | 0.095 | 0.019 | 3.50E-07 | 25.000 | 3.45E-04 |
| AN | rs2821359 | 1 | T | C | NA | -0.082 | 0.016 | 3.76E-07 | 26.266 | 3.62E-04 |
| AN | rs725861 | 10 | A | G | NA | 0.088 | 0.018 | 4.86E-07 | 23.901 | 3.29E-04 |
| AN | rs62176532 | 2 | T | C | NA | 0.086 | 0.017 | 5.40E-07 | 25.592 | 3.53E-04 |
| AN | rs11236814 | 11 | A | T | NA | -0.120 | 0.024 | 5.53E-07 | 25.000 | 3.45E-04 |
| AN | rs12826213 | 12 | C | T | NA | 0.069 | 0.014 | 6.21E-07 | 24.291 | 3.35E-04 |
| AN | rs2188244 | 7 | C | A | NA | -0.068 | 0.014 | 6.44E-07 | 23.592 | 3.25E-04 |
| AN | rs9784437 | 4 | A | G | NA | -0.083 | 0.017 | 8.01E-07 | 23.837 | 3.29E-04 |
| AN | rs9309871 | 3 | C | A | NA | -0.078 | 0.016 | 8.58E-07 | 23.766 | 3.28E-04 |
| AN | rs2884594 | 12 | A | G | NA | -0.087 | 0.018 | 9.37E-07 | 23.361 | 3.22E-04 |
| AN | rs4751 | 1 | G | T | NA | -0.082 | 0.017 | 1.16E-06 | 23.266 | 3.21E-04 |
| AN | rs72635674 | 13 | T | C | NA | -0.080 | 0.017 | 1.47E-06 | 22.145 | 3.05E-04 |
| AN | rs17049722 | 2 | C | T | NA | 0.093 | 0.019 | 1.51E-06 | 23.958 | 3.30E-04 |
| AN | rs7559141 | 2 | T | A | NA | -0.294 | 0.061 | 1.58E-06 | 23.229 | 3.20E-04 |
| AN | rs72734967 | 9 | G | A | NA | -0.113 | 0.024 | 1.66E-06 | 22.168 | 3.06E-04 |
| AN | rs3132604 | 6 | C | T | NA | 0.086 | 0.018 | 1.71E-06 | 22.827 | 3.15E-04 |
| AN | rs112502176 | 14 | G | T | NA | -0.081 | 0.017 | 1.97E-06 | 22.702 | 3.13E-04 |
| AN | rs35643176 | 1 | A | T | NA | -0.125 | 0.026 | 2.09E-06 | 23.114 | 3.19E-04 |
| AN | rs6520157 | 22 | G | A | NA | -0.180 | 0.038 | 2.12E-06 | 22.438 | 3.09E-04 |
| AN | rs189391781 | 9 | A | G | NA | -0.239 | 0.051 | 2.28E-06 | 21.961 | 3.03E-04 |
| AN | rs7797895 | 7 | A | C | NA | 0.078 | 0.017 | 2.33E-06 | 21.052 | 2.90E-04 |
| AN | rs34029884 | 2 | A | C | NA | -0.071 | 0.015 | 2.43E-06 | 22.404 | 3.09E-04 |
| AN | rs61754806 | 5 | G | A | NA | -0.156 | 0.033 | 2.50E-06 | 22.347 | 3.08E-04 |
| AN | rs34306607 | 2 | A | G | NA | -0.065 | 0.014 | 2.53E-06 | 21.556 | 2.97E-04 |
| AN | rs7300592 | 12 | G | A | NA | -0.094 | 0.020 | 2.80E-06 | 22.090 | 3.05E-04 |
| AN | rs570333 | 11 | C | T | NA | -0.065 | 0.014 | 2.82E-06 | 21.556 | 2.97E-04 |
| AN | rs9929084 | 16 | C | G | NA | 0.069 | 0.015 | 2.99E-06 | 21.160 | 2.92E-04 |
| AN | rs12315996 | 12 | C | T | NA | -0.079 | 0.017 | 3.19E-06 | 21.595 | 2.98E-04 |
| AN | rs4145641 | 13 | A | C | NA | 0.075 | 0.016 | 3.38E-06 | 21.973 | 3.03E-04 |
| AN | rs6792954 | 3 | G | A | NA | 0.076 | 0.016 | 3.41E-06 | 22.563 | 3.11E-04 |
| AN | rs13422947 | 2 | G | A | NA | 0.088 | 0.019 | 3.50E-06 | 21.452 | 2.96E-04 |
| AN | rs6773424 | 3 | G | A | NA | -0.091 | 0.020 | 3.70E-06 | 20.703 | 2.85E-04 |
| AN | rs12822914 | 12 | A | G | NA | -0.067 | 0.015 | 4.17E-06 | 19.951 | 2.75E-04 |
| AN | rs10882687 | 10 | G | A | NA | 0.076 | 0.017 | 4.24E-06 | 19.986 | 2.76E-04 |
| AN | rs12345267 | 9 | T | C | NA | 0.063 | 0.014 | 4.28E-06 | 20.250 | 2.79E-04 |
| AN | rs17580614 | 9 | A | G | NA | -0.086 | 0.019 | 4.41E-06 | 20.488 | 2.82E-04 |
| AN | rs3848726 | 20 | G | T | NA | 0.066 | 0.014 | 4.44E-06 | 22.224 | 3.06E-04 |
| AN | rs76453976 | 3 | A | G | NA | -0.164 | 0.036 | 4.53E-06 | 20.753 | 2.86E-04 |
| AN | rs17842856 | 5 | T | C | NA | 0.077 | 0.017 | 4.95E-06 | 20.516 | 2.83E-04 |

**Abbreviation**

EA, effect allele; OA, other allele; EAF, effect allele frequency; SE, standard error; Chr, chromosome; AN, anorexia nervosa; MDD, major depressive disorder; PTSD, posttraumatic stress disorder; SCZ, schizophrenia; PD, panic disorder; SNP, single nucleotide polymorphism.

**Table S3. The results of Mendelian randomization on the association of neuroticism clusters on psychiatric disorders.**

| **Exposure** | **Outcome** | **Method** | **Number of IVs (outliers removed)** | **Beta** | **SE** | **P** | **OR** | **OR LCI** | **OR UCI** |
| --- | --- | --- | --- | --- | --- | --- | --- | --- | --- |
| depressed affect | ADHD | IVW (mre) | 53 | 0.763 | 0.140 | 4.55E-08 | 2.145 | 1.631 | 2.819 |
|  |  | WM | 53 | 0.608 | 0.151 | 5.78E-05 | 1.836 | 1.365 | 2.469 |
|  |  | MR Egger | 53 | 0.233 | 0.694 | 0.739 | 1.262 | 0.324 | 4.919 |
| depressed affect | AN | IVW (mre) | 47 | 0.231 | 0.207 | 0.264 | 1.259 | 0.840 | 1.888 |
|  |  | MR Egger | 47 | 0.438 | 1.006 | 0.666 | 1.549 | 0.216 | 11.131 |
|  |  | WM | 47 | 0.280 | 0.227 | 0.216 | 1.323 | 0.849 | 2.063 |
| depressed affect | ASD | IVW (mre) | 48 | 0.174 | 0.190 | 0.361 | 1.190 | 0.820 | 1.727 |
|  |  | MR Egger | 48 | -0.171 | 0.904 | 0.851 | 0.843 | 0.143 | 4.962 |
|  |  | WM | 48 | -0.101 | 0.225 | 0.653 | 0.904 | 0.582 | 1.404 |
| depressed affect | BIP | IVW (mre) | 50 | 0.506 | 0.155 | 0.001 | 1.659 | 1.223 | 2.250 |
|  |  | WM | 50 | 0.524 | 0.195 | 0.007 | 1.689 | 1.151 | 2.478 |
|  |  | MR Egger | 50 | 0.843 | 0.713 | 0.243 | 2.324 | 0.575 | 9.398 |
| depressed affect | MDD | IVW (mre) | 47 | 1.045 | 0.111 | 3.58E-21 | 2.844 | 2.290 | 3.533 |
|  |  | WM | 47 | 1.123 | 0.130 | 5.94E-18 | 3.074 | 2.382 | 3.966 |
|  |  | MR Egger | 47 | 0.582 | 0.537 | 0.285 | 1.789 | 0.624 | 5.129 |
| depressed affect | OCD | IVW (mre) | 47 | 0.268 | 0.407 | 0.511 | 1.307 | 0.588 | 2.904 |
|  |  | MR Egger | 47 | -2.193 | 1.933 | 0.262 | 0.112 | 0.003 | 4.928 |
|  |  | WM | 47 | 0.100 | 0.547 | 0.856 | 1.105 | 0.378 | 3.228 |
| depressed affect | PD | IVW (mre) | 47 | 0.953 | 0.421 | 0.024 | 2.593 | 1.137 | 5.913 |
|  |  | WM | 47 | 0.667 | 0.548 | 0.224 | 1.948 | 0.665 | 5.707 |
|  |  | MR Egger | 47 | 2.319 | 1.949 | 0.240 | 10.168 | 0.223 | 463.980 |
| depressed affect | PTSD | IVW (mre) | 59 | 1.080 | 0.146 | 1.48E-13 | 2.943 | 2.210 | 3.919 |
|  |  | WM | 59 | 1.049 | 0.202 | 1.94E-07 | 2.856 | 1.924 | 4.240 |
|  |  | MR Egger | 59 | 1.775 | 0.670 | 0.010 | 5.901 | 1.588 | 21.924 |
| depressed affect | TS | IVW (mre) | 47 | 0.178 | 0.371 | 0.631 | 1.195 | 0.578 | 2.473 |
|  |  | WM | 47 | 0.116 | 0.412 | 0.779 | 1.123 | 0.500 | 2.519 |
|  |  | MR Egger | 47 | 1.573 | 1.812 | 0.390 | 4.822 | 0.138 | 167.999 |
| depressed affect | SCZ | IVW (mre) | 41 | 0.479 | 0.152 | 0.002 | 1.614 | 1.199 | 2.173 |
|  |  | WM | 41 | 0.474 | 0.165 | 0.004 | 1.606 | 1.163 | 2.220 |
|  |  | MR Egger | 41 | -0.674 | 0.673 | 0.323 | 0.510 | 0.136 | 1.907 |
| SESA | ADHD | IVW (mre) | 40 | 0.327 | 0.148 | 0.027 | 1.386 | 1.038 | 1.852 |
|  |  | WM | 40 | 0.339 | 0.172 | 0.049 | 1.404 | 1.001 | 1.968 |
|  |  | MR Egger | 40 | 0.645 | 0.779 | 0.413 | 1.906 | 0.414 | 8.771 |
| SESA | AN | IVW (mre) | 39 | 1.019 | 0.179 | 1.20E-08 | 2.772 | 1.952 | 3.936 |
|  |  | WM | 39 | 0.910 | 0.227 | 6.00E-05 | 2.484 | 1.614 | 3.824 |
|  |  | MR Egger | 39 | 2.478 | 0.893 | 0.009 | 11.915 | 2.071 | 68.535 |
| SESA | ASD | IVW (mre) | 38 | 0.237 | 0.185 | 0.200 | 1.268 | 0.882 | 1.822 |
|  |  | MR Egger | 38 | 1.238 | 1.045 | 0.244 | 3.450 | 0.445 | 26.728 |
|  |  | WM | 38 | 0.205 | 0.233 | 0.379 | 1.227 | 0.777 | 1.938 |
| SESA | BIP | IVW (mre) | 38 | 0.381 | 0.202 | 0.059 | 1.464 | 0.986 | 2.174 |
|  |  | WM | 38 | 0.346 | 0.238 | 0.145 | 1.414 | 0.888 | 2.252 |
|  |  | MR Egger | 38 | 0.544 | 1.158 | 0.641 | 1.723 | 0.178 | 16.683 |
| SESA | MDD | IVW (mre) | 38 | 0.818 | 0.115 | 9.49E-13 | 2.267 | 1.811 | 2.838 |
|  |  | WM | 38 | 0.844 | 0.142 | 2.99E-09 | 2.326 | 1.760 | 3.074 |
|  |  | MR Egger | 38 | 1.736 | 0.639 | 0.010 | 5.675 | 1.623 | 19.839 |
| SESA | OCD | IVW (mre) | 38 | 0.582 | 0.508 | 0.252 | 1.789 | 0.661 | 4.842 |
|  |  | MR Egger | 38 | 0.653 | 2.946 | 0.826 | 1.922 | 0.006 | 618.569 |
|  |  | WM | 38 | -0.144 | 0.590 | 0.807 | 0.866 | 0.272 | 2.753 |
| SESA | PD | IVW (mre) | 38 | 0.624 | 0.451 | 0.166 | 1.867 | 0.771 | 4.519 |
|  |  | WM | 38 | 0.920 | 0.584 | 0.115 | 2.510 | 0.800 | 7.880 |
|  |  | MR Egger | 38 | 2.506 | 2.513 | 0.325 | 12.251 | 0.089 | 1686.659 |
| SESA | PTSD | IVW (mre) | 42 | 0.991 | 0.164 | 1.43E-09 | 2.693 | 1.954 | 3.711 |
|  |  | WM | 42 | 1.025 | 0.222 | 3.72E-06 | 2.787 | 1.805 | 4.302 |
|  |  | MR Egger | 42 | 2.288 | 0.847 | 0.010 | 9.852 | 1.872 | 51.837 |
| SESA | SCZ | IVW (mre) | 30 | 0.934 | 0.125 | 6.72E-14 | 2.545 | 1.993 | 3.249 |
|  |  | WM | 30 | 0.807 | 0.165 | 9.86E-07 | 2.242 | 1.623 | 3.098 |
|  |  | MR Egger | 30 | 1.202 | 0.710 | 0.102 | 3.326 | 0.827 | 13.381 |
| SESA | TS | IVW (mre) | 38 | 0.547 | 0.346 | 0.114 | 1.728 | 0.876 | 3.407 |
|  |  | WM | 38 | -0.291 | 0.436 | 0.505 | 0.748 | 0.318 | 1.757 |
|  |  | MR Egger | 38 | -1.496 | 1.978 | 0.454 | 0.224 | 0.005 | 10.815 |
| worry | ADHD | IVW (mre) | 52 | 0.040 | 0.107 | 0.707 | 1.041 | 0.844 | 1.284 |
|  |  | MR Egger | 52 | -0.468 | 0.583 | 0.425 | 0.626 | 0.200 | 1.961 |
|  |  | WM | 52 | 0.094 | 0.129 | 0.466 | 1.098 | 0.854 | 1.413 |
| worry | AN | IVW (mre) | 50 | -0.422 | 0.177 | 0.017 | 0.656 | 0.464 | 0.927 |
|  |  | MR Egger | 50 | 0.224 | 0.886 | 0.801 | 1.252 | 0.220 | 7.108 |
|  |  | WM | 50 | -0.211 | 0.197 | 0.285 | 0.810 | 0.550 | 1.192 |
| worry | ASD | IVW (mre) | 52 | 0.153 | 0.177 | 0.388 | 1.165 | 0.823 | 1.649 |
|  |  | MR Egger | 52 | 0.343 | 0.895 | 0.703 | 1.410 | 0.244 | 8.149 |
|  |  | WM | 52 | 0.130 | 0.204 | 0.523 | 1.139 | 0.764 | 1.698 |
| worry | BIP | IVW (mre) | 49 | 0.527 | 0.186 | 0.005 | 1.694 | 1.176 | 2.439 |
|  |  | WM | 49 | 0.546 | 0.209 | 0.009 | 1.726 | 1.147 | 2.597 |
|  |  | MR Egger | 49 | -0.639 | 0.923 | 0.493 | 0.528 | 0.086 | 3.225 |
| worry | MDD | IVW (mre) | 51 | 0.949 | 0.085 | 4.44E-29 | 2.584 | 2.188 | 3.052 |
|  |  | WM | 51 | 0.932 | 0.117 | 1.26E-15 | 2.539 | 2.021 | 3.191 |
|  |  | MR Egger | 51 | 0.766 | 0.428 | 0.080 | 2.151 | 0.929 | 4.979 |
| worry | OCD | IVW (mre) | 51 | 1.107 | 0.405 | 0.006 | 3.026 | 1.369 | 6.690 |
|  |  | MR Egger | 51 | 1.975 | 2.083 | 0.348 | 7.206 | 0.121 | 427.641 |
|  |  | WM | 51 | 0.761 | 0.510 | 0.136 | 2.140 | 0.788 | 5.812 |
| worry | PD | IVW (mre) | 51 | 1.532 | 0.421 | 2.76E-04 | 4.626 | 2.026 | 10.561 |
|  |  | WM | 51 | 1.388 | 0.513 | 0.007 | 4.005 | 1.466 | 10.939 |
|  |  | MR Egger | 51 | 4.949 | 2.127 | 0.024 | 141.035 | 2.181 | 9118.460 |
| worry | PTSD | IVW (mre) | 58 | 0.593 | 0.144 | 3.61E-05 | 1.810 | 1.366 | 2.398 |
|  |  | WM | 58 | 0.759 | 0.192 | 7.71E-05 | 2.137 | 1.466 | 3.114 |
|  |  | MR Egger | 58 | 0.582 | 0.712 | 0.417 | 1.790 | 0.443 | 7.232 |
| worry | SCZ | IVW (mre) | 41 | 0.594 | 0.146 | 4.77E-05 | 1.811 | 1.360 | 2.412 |
|  |  | WM | 41 | 0.504 | 0.154 | 0.001 | 1.655 | 1.224 | 2.237 |
|  |  | MR Egger | 41 | 0.809 | 0.666 | 0.231 | 2.246 | 0.609 | 8.278 |
| worry | TS | IVW (mre) | 50 | 0.837 | 0.313 | 0.008 | 2.310 | 1.250 | 4.268 |
|  |  | WM | 50 | 0.074 | 0.396 | 0.852 | 1.077 | 0.495 | 2.342 |
|  |  | MR Egger | 50 | 0.608 | 1.585 | 0.703 | 1.836 | 0.082 | 41.068 |

**Abbreviation**

SE, standard error; ADHD, attention-deficit/hyperactivity disorder; AN, anorexia nervosa; ASD, autism spectrum disorder; BIP, bipolar disorder; MDD, major depressive disorder; OCD, obsessive compulsory disorder; PTSD, posttraumatic stress disorder; TS, Tourette syndrome; SCZ, schizophrenia; PD, panic disorder; OR, odds ratio; IVW (mre), the inverse variance weighted (multiplicative random effects); WM, weighted median; CI, confidence interval; IVs, instrumental variables.

**Table S4. The results of Mendelian randomization on the association among psychiatric disorders.**

| **Exposure** | **Outcome** | **Method** | **Number of IVs**  **(outliers removed)** | **Beta** | **SE** | ***P*** | **OR** | **OR LCI** | **OR UCI** |
| --- | --- | --- | --- | --- | --- | --- | --- | --- | --- |
| PD | PTSD | IVW (mre) | 11 | 0.043 | 0.013 | 9.20E-04 | 1.043 | 1.018 | 1.070 |
|  |  | MR Egger | 11 | 0.012 | 0.063 | 0.854 | 1.012 | 0.894 | 1.145 |
|  |  | WM | 11 | 0.049 | 0.032 | 0.125 | 1.050 | 0.986 | 1.119 |
| MDD | PTSD | IVW (mre) | 33 | 0.392 | 0.104 | 1.61E-04 | 1.480 | 1.207 | 1.813 |
|  |  | MR Egger | 33 | 0.457 | 0.535 | 0.399 | 1.579 | 0.554 | 4.505 |
|  |  | WM | 33 | 0.452 | 0.131 | 5.70E-04 | 1.572 | 1.215 | 2.034 |
| AN | SCZ | IVW (mre) | 42 | 0.119 | 0.028 | 2.59E-05 | 1.127 | 1.066 | 1.191 |
|  |  | WM | 42 | 0.090 | 0.033 | 6.87E-03 | 1.094 | 1.031 | 1.161 |
|  |  | MR Egger | 42 | 0.073 | 0.142 | 0.611 | 1.076 | 0.814 | 1.421 |
| AN | PTSD | IVW (mre) | 44 | 0.100 | 0.036 | 5.57E-03 | 1.106 | 1.030 | 1.187 |
|  |  | WM | 44 | 0.128 | 0.180 | 0.481 | 1.105 | 1.004 | 1.216 |
|  |  | MR Egger | 44 | 0.100 | 0.049 | 0.040 | 1.137 | 0.798 | 1.619 |
| SCZ | AN | IVW (mre) | 146 | 0.118 | 0.028 | 2.25E-05 | 1.121 | 1.060 | 1.187 |
|  |  | WM | 146 | 0.013 | 0.116 | 0.907 | 1.147 | 1.077 | 1.223 |
|  |  | MR Egger | 146 | 0.146 | 0.031 | 2.64E-06 | 1.002 | 0.800 | 1.254 |
| SCZ | PTSD | IVW (mre) | 148 | 0.143 | 0.023 | 9.14E-10 | 1.153 | 1.102 | 1.207 |
|  |  | WM | 148 | 0.132 | 0.032 | 3.39E-05 | 1.142 | 1.072 | 1.215 |
|  |  | MR Egger | 148 | 0.320 | 0.092 | 6.34E-04 | 1.377 | 1.151 | 1.648 |

**Abbreviation**

SE, standard error; AN, anorexia nervosa; MDD, major depressive disorder; PTSD, posttraumatic stress disorder; SCZ, schizophrenia; PD, panic disorder; OR, odds ratio; IVW (mre), the inverse variance weighted (multiplicative random effects); WM, weighted median; CI, confidence interval; IVs, instrumental variables.

**Table S5. The results of multivariable Mendelian randomization.**

| **Exposure** | **Outcome** | **nSNP** | **Beta** | **SE** | **P** | **OR (95%CI)** |
| --- | --- | --- | --- | --- | --- | --- |
| SESA | PTSD | 45 | 0.956 | 0.258 | 2.11E-04 | 2.601 (2.096, 3.107) |
| worry | PTSD | 34 | 0.525 | 0.327 | 0.109 | 1.690 (1.050, 2.331) |
| depressed affect | PTSD | 47 | -0.102 | 0.258 | 0.694 | 0.903 (0.397, 1.409) |

**Abbreviation**

SESA, sensitivity to environmental stress and adversity; PTSD, posttraumatic stress disorder; SNP, SNP, single nucleotide polymorphism; SE, standard error**.**

**Table S6. The results of mediation Mendelian randomization.**

|  |  |  | EffectA（exposure→mediator) | | | EffectB (mediator→outcome) | | | EffectC (exposure→outcome) | | | Effect A * Effect B / Effect C |  |
| --- | --- | --- | --- | --- | --- | --- | --- | --- | --- | --- | --- | --- | --- |
| Exposure | Outcome | Mediator | Beta | SE | P | Beta | SE | P | Beta | SE | P | Proportion mediated | |
| depressed affect | PTSD | PD | 0.953 | 0.421 | 0.024 | 0.043 | 0.013 | 9.2E-04 | 1.080 | 0.146 | 1.48E-13 | 3.76% | |
| SESA | PTSD | AN | 1.019 | 0.179 | 1.20E-08 | 0.100 | 0.036 | 0.006 | 0.991 | 0.164 | 1.43E-09 | 10.33% | |

**Abbreviation**

SESA, sensitivity to environmental stress and adversity; SE, standard error; PTSD, posttraumatic stress disorder; PD, panic disorder; AN, anorexia nervosa.

**Table S7. Tests of heterogeneity for neuroticism clusters as exposures.**

| **Exposure** | **Outcome** | **Method** | **Q** | **Q_df** | **Q_pval** |
| --- | --- | --- | --- | --- | --- |
| depressed affect | ADHD | MR Egger | 123.468 | 51 | 6.03E-08 |
|  |  | IVW | 124.941 | 52 | 6.10E-08 |
| depressed affect | AN | MR Egger | 77.651 | 44 | 0.001 |
|  |  | IVW | 77.681 | 45 | 0.002 |
| depressed affect | ASD | MR Egger | 85.992 | 46 | 3.20E-04 |
|  |  | IVW | 86.276 | 47 | 4.18E-04 |
| depressed affect | BIP | MR Egger | 67.983 | 48 | 0.030 |
|  |  | IVW | 68.316 | 49 | 0.035 |
| depressed affect | MDD | MR Egger | 83.789 | 45 | 3.97E-04 |
|  |  | IVW | 85.237 | 46 | 3.87E-04 |
| depressed affect | OCD | MR Egger | 61.832 | 45 | 0.048 |
|  |  | IVW | 64.162 | 46 | 0.039 |
| depressed affect | PD | MR Egger | 58.998 | 45 | 0.079 |
|  |  | IVW | 59.674 | 46 | 0.085 |
| depressed affect | PTSD | MR Egger | 67.490 | 57 | 0.161 |
|  |  | IVW | 68.831 | 58 | 0.156 |
| depressed affect | TS | MR Egger | 88.214 | 45 | 1.26E-04 |
|  |  | IVW | 89.427 | 46 | 1.31E-04 |
| depressed affect | SCZ | MR Egger | 94.073 | 39 | 1.90E-06 |
|  |  | IVW | 101.505 | 40 | 2.97E-07 |
| SESA | ADHD | MR Egger | 85.934 | 38 | 1.43E-05 |
|  |  | IVW | 86.326 | 39 | 1.96E-05 |
| SESA | AN | MR Egger | 53.303 | 37 | 0.040 |
|  |  | IVW | 57.301 | 38 | 0.023 |
| SESA | ASD | MR Egger | 52.672 | 36 | 0.036 |
|  |  | IVW | 54.060 | 37 | 0.035 |
| SESA | BIP | MR Egger | 68.049 | 36 | 0.001 |
|  |  | IVW | 68.088 | 37 | 0.001 |
| SESA | MDD | MR Egger | 59.114 | 36 | 0.009 |
|  |  | IVW | 62.615 | 37 | 0.005 |
| SESA | OCD | MR Egger | 68.393 | 36 | 0.001 |
|  |  | IVW | 68.394 | 37 | 0.001 |
| SESA | PD | MR Egger | 46.719 | 36 | 0.109 |
|  |  | IVW | 47.471 | 37 | 0.116 |
| SESA | PTSD | MR Egger | 44.119 | 40 | 0.302 |
|  |  | IVW | 46.801 | 41 | 0.247 |
| SESA | SCZ | MR Egger | 38.273 | 28 | 0.093 |
|  |  | IVW | 38.473 | 29 | 0.112 |
| SESA | TS | MR Egger | 52.090 | 36 | 0.040 |
|  |  | IVW | 53.682 | 37 | 0.037 |
| worry | ADHD | MR Egger | 74.388 | 50 | 0.014 |
|  |  | IVW | 75.562 | 51 | 0.014 |
| worry | AN | MR Egger | 89.111 | 48 | 2.88E-04 |
|  |  | IVW | 90.140 | 49 | 3.12E-04 |
| worry | ASD | MR Egger | 95.453 | 50 | 1.14E-04 |
|  |  | IVW | 95.543 | 51 | 1.58E-04 |
| worry | BIP | MR Egger | 93.257 | 47 | 6.86E-05 |
|  |  | IVW | 96.551 | 48 | 4.12E-05 |
| worry | MDD | MR Egger | 61.436 | 49 | 0.109 |
|  |  | IVW | 61.675 | 50 | 0.124 |
| worry | OCD | MR Egger | 78.846 | 49 | 0.004 |
|  |  | IVW | 79.136 | 50 | 0.005 |
| worry | PD | MR Egger | 69.918 | 49 | 0.026 |
|  |  | IVW | 73.746 | 50 | 0.016 |
| worry | PTSD | MR Egger | 68.465 | 56 | 0.123 |
|  |  | IVW | 68.466 | 57 | 0.142 |
| worry | SCZ | MR Egger | 99.138 | 39 | 3.86E-07 |
|  |  | IVW | 99.417 | 40 | 5.76E-07 |
| worry | TS | MR Egger | 76.396 | 48 | 0.006 |
|  |  | IVW | 76.430 | 49 | 0.007 |

**Abbreviation**

ADHD, attention-deficit/hyperactivity disorder; AN, anorexia nervosa; ASD, autism spectrum disorder; BIP, bipolar disorder; MDD, major depressive disorder; OCD, obsessive compulsory disorder; PTSD, posttraumatic stress disorder; TS, Tourette syndrome; SCZ, schizophrenia; PD, panic disorder; IVW, the inverse variance weighted.

**Table S8. Tests of heterogeneity for psychiatric disorders as exposures.**

| **Exposure** | **Outcome** | **Method** | **Q** | **Q_df** | **Q_pval** |
| --- | --- | --- | --- | --- | --- |
| PD | PTSD | MR Egger | 2.226 | 9 | 0.987 |
|  |  | IVW | 2.508 | 10 | 0.991 |
| MDD | PTSD | MR Egger | 44.540 | 31 | 0.055 |
|  |  | IVW | 44.562 | 32 | 0.069 |
| AN | SCZ | MR Egger | 87.381 | 40 | 2.21E-05 |
|  |  | IVW | 87.626 | 41 | 3.12E-05 |
| AN | PTSD | MR Egger | 52.470 | 42 | 0.129 |
|  |  | IVW | 52.501 | 43 | 0.152 |
| SCZ | AN | MR Egger | 266.080 | 144 | 2.62E-09 |
|  |  | IVW | 269.451 | 145 | 1.62E-09 |
| SCZ | PTSD | MR Egger | 176.180 | 146 | 0.045 |
|  |  | IVW | 181.004 | 147 | 0.030 |

**Abbreviation**

AN, anorexia nervosa; MDD, major depressive disorder; PTSD, posttraumatic stress disorder; SCZ, schizophrenia; PD, panic disorder; IVW, the inverse variance weighted.

**Table S9. Tests of pleiotropic effects for psychiatric disorders as exposures.**

| **Exposure** | **Outcome** | **MR-PRESSO** | | | | **Global test** | | | **Pleiotropy** | | |
| --- | --- | --- | --- | --- | --- | --- | --- | --- | --- | --- | --- |
|  |  | **Beta** | **Main.MR.results.Sd** | **Main.MR.results.T.stat** | **P** | **RSSobs** | **P** | **outliers removed** | **Intercept(MR-Egger)** | **SE** | **P** |
| depressed affect | ADHD | 0.760 | 0.137 | 5.540 | 9.65E-07 | 129.820 | <1e-04 | 0 | 0.009 | 0.011 | 0.439 |
| depressed affect | AN | 0.195 | 0.161 | 1.211 | 0.231 | 96.503 | 0.001 | 0 | -0.002 | 0.014 | 0.896 |
| depressed affect | ASD | 0.160 | 0.167 | 0.958 | 0.342 | 103.271 | 6.00E-04 | 1 | 0.006 | 0.014 | 0.699 |
| depressed affect | BIP | 0.447 | 0.161 | 2.776 | 0.008 | 82.924 | 0.006 | 1 | -0.006 | 0.012 | 0.630 |
| depressed affect | MDD | 0.976 | 0.099 | 9.902 | 5.38E-14 | 105.767 | 0.001 | 0 | 0.008 | 0.009 | 0.383 |
| depressed affect | OCD | 0.300 | 0.341 | 0.879 | 0.383 | 70.769 | 0.144 | 0 | 0.040 | 0.031 | 0.200 |
| depressed affect | PD | 0.684 | 0.382 | 1.789 | 0.079 | 77.519 | 0.062 | 0 | -0.022 | 0.031 | 0.476 |
| depressed affect | PTSD | 1.065 | 0.145 | 7.357 | 6.72E-10 | 71.929 | 0.205 | 0 | -0.012 | 0.011 | 0.292 |
| depressed affect | TS | 0.392 | 0.336 | 1.167 | 0.248 | 115.028 | <1e-04 | 1 | -0.023 | 0.029 | 0.436 |
| depressed affect | SCZ | 0.476 | 0.148 | 3.209 | 0.003 | 106.783 | <1e-04 | 12 | 0.019 | 0.011 | 0.087 |
| SESA | ADHD | 0.355 | 0.151 | 2.354 | 0.023 | 105.120 | <1e-04 | 1 | -0.005 | 0.013 | 0.679 |
| SESA | AN | 1.062 | 0.166 | 6.390 | 1.09E-07 | 63.135 | 0.051 | 0 | 0.025 | 0.015 | 0.104 |
| SESA | ASD | 0.181 | 0.171 | 1.060 | 0.295 | 59.066 | 0.058 | 0 | -0.016 | 0.017 | 0.337 |
| SESA | BIP | 0.674 | 0.133 | 5.051 | 1.07E-05 | 150.329 | <1e-04 | 3 | -0.003 | 0.019 | 0.887 |
| SESA | MDD | 0.799 | 0.106 | 7.524 | 3.03E-09 | 68.911 | 0.016 | 1 | -0.015 | 0.010 | 0.153 |
| SESA | OCD | 0.656 | 0.474 | 1.385 | 0.173 | 76.714 | 0.002 | 1 | -0.001 | 0.048 | 0.980 |
| SESA | PD | 0.593 | 0.424 | 1.401 | 0.169 | 54.063 | 0.132 | 0 | -0.031 | 0.041 | 0.452 |
| SESA | PTSD | 0.968 | 0.161 | 6.010 | 3.84E-07 | 49.814 | 0.297 | 0 | -0.022 | 0.014 | 0.127 |
| SESA | SCZ | 0.920 | 0.121 | 7.620 | 1.69E-08 | 41.244 | 0.184 | 12 | -0.004 | 0.012 | 0.705 |
| SESA | TS | 0.435 | 0.325 | 1.338 | 0.188 | 61.054 | 0.044 | 1 | 0.034 | 0.032 | 0.301 |
| worry | ADHD | 0.040 | 0.105 | 0.383 | 0.704 | 78.285 | 0.017 | 3 | 0.009 | 0.010 | 0.379 |
| worry | AN | -0.528 | 0.171 | -3.085 | 0.003 | 109.955 | <1e-04 | 0 | -0.012 | 0.016 | 0.460 |
| worry | ASD | 0.127 | 0.160 | 0.794 | 0.430 | 100.413 | 6.00E-04 | 1 | -0.003 | 0.016 | 0.829 |
| worry | BIP | 0.691 | 0.126 | 5.491 | 1.15E-06 | 113.948 | <1e-04 | 5 | 0.021 | 0.016 | 0.204 |
| worry | MDD | 0.972 | 0.078 | 12.504 | 5.63E-18 | 69.161 | 0.313 | 0 | 0.003 | 0.008 | 0.664 |
| worry | OCD | 1.143 | 0.365 | 3.127 | 0.003 | 86.557 | 0.014 | 1 | -0.015 | 0.036 | 0.673 |
| worry | PD | 1.276 | 0.420 | 3.041 | 0.004 | 98.026 | 0.002 | 1 | -0.061 | 0.037 | 0.108 |
| worry | PTSD | 0.586 | 0.142 | 4.139 | 1.14E-04 | 71.105 | 0.169 | 0 | 1.94E-04 | 0.013 | 0.988 |
| worry | SCZ | 0.594 | 0.146 | 4.067 | 2.17E-04 | 364.697 | <1e-04 | 10 | -0.004 | 0.012 | 0.742 |
| worry | TS | 0.650 | 0.287 | 2.262 | 0.028 | 86.348 | 0.012 | 1 | 0.004 | 0.028 | 0.883 |

**Abbreviation:** ADHD, attention-deficit/hyperactivity disorder; AN, anorexia nervosa; ASD, autism spectrum disorder; BIP, bipolar disorder; MDD, major depressive disorder; OCD, obsessive compulsory disorder; PTSD, posttraumatic stress disorder; TS, Tourette syndrome; SCZ, schizophrenia; PD, panic disorder; SE. standard error; MR PRESSO, MR pleiotropy residual sum and outlier.

**Table S10. Tests of pleiotropic effects for psychiatric disorders as exposures.**

| **Exposure** | **Outcome** | **MR-PRESSO** | | | | **Global test** | | | **Pleiotropy** | | | |
| --- | --- | --- | --- | --- | --- | --- | --- | --- | --- | --- | --- | --- |
|  |  | **Beta** | **Main.MR.**  **results.Sd** | **Main.MR.**  **results.T.stat** | ***P*** | **RSSobs** | ***P*** | **outliers removed** | **Intercept**  **(MR-Egge)** | | **SE** | ***P*** |
| PD | PTSD | 0.043 | 0.013 | 3.314 | 0.008 | 3.145 | 0.990 | 0 | 0.013 | | 0.024 | 0.609 |
| MDD | PTSD | 0.404 | 0.103 | 3.915 | 1.62E-04 | 55.770 | 0.033 | 0 | -0.002 | 0.017 | | 0.902 |
| AN | SCZ | 0.127 | 0.025 | 5.104 | 5.18E-06 | 107.584 | <1e-04 | 1 | -0.004 | | 0.012 | 0.739 |
| AN | PTSD | 0.074 | 0.031 | 2.367 | 0.022 | 62.119 | 0.220 | 0 | 0.002 | | 0.015 | 0.876 |
| SCZ | AN | 0.118 | 0.024 | 4.961 | 1.64E-06 | 316.980 | <1e-04 | 2 | -0.010 | | 0.007 | 0.179 |
| SCZ | PTSD | 0.135 | 0.023 | 5.893 | 2.28E-08 | 198.437 | 0.017 | 0 | -0.012 | | 0.006 | 0.047 |

**Abbreviation:** AN, anorexia nervosa; MDD, major depressive disorder; PTSD, posttraumatic stress disorder; SCZ, schizophrenia; PD, panic disorder; SE. standard error; MR PRESSO, MR pleiotropy residual sum and outlier.

**4.Supplementary Figures 1-6**

**Figure S1. Leave-one-out plot of significant results from SESA on psychiatric disorders. a. SESA on AN; b.SESA on SCZ; c. SESA on PTSD;**

1. **B.**

**
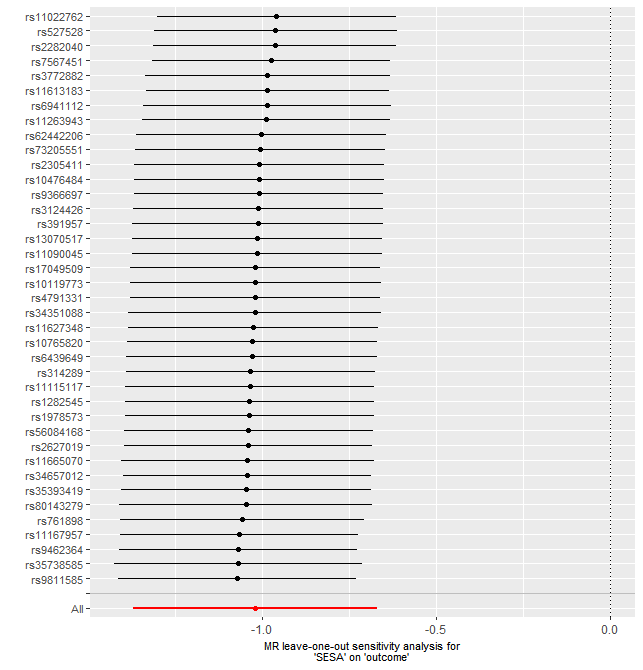

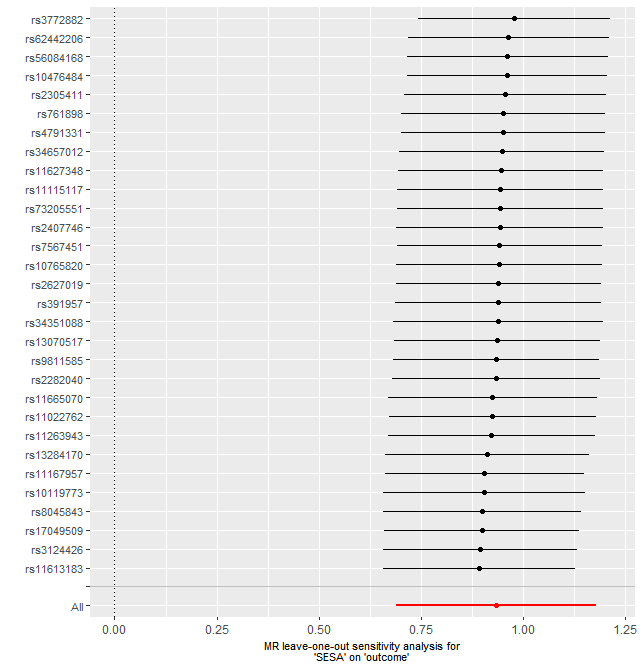
**

**C.**


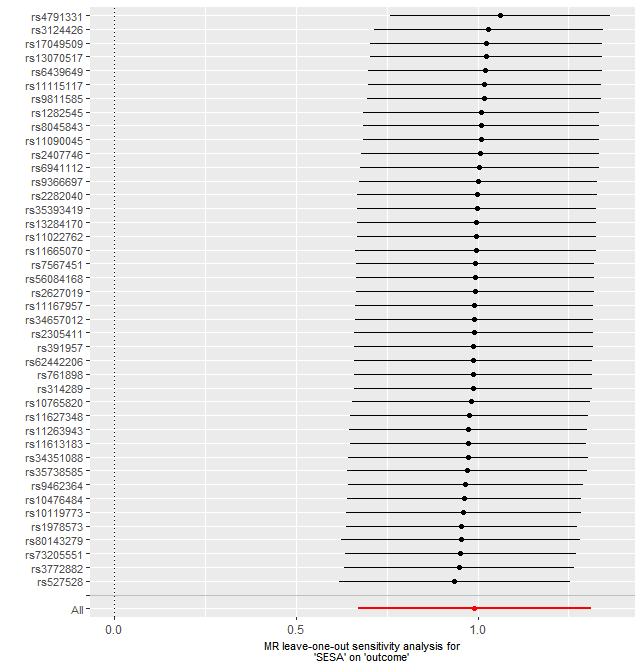


**Abbreviation:** SESA, sensitivity to environmental stress and adversity; AN, anorexia nervosa; SCZ, schizophrenia; PTSD, posttraumatic stress disorder;

**Figure S2. Leave-one-out plot of significant results from depressed affect on psychiatric disorders. a. depressed affect on PD; b.depressed affect on PTSD;**

1. **B.**


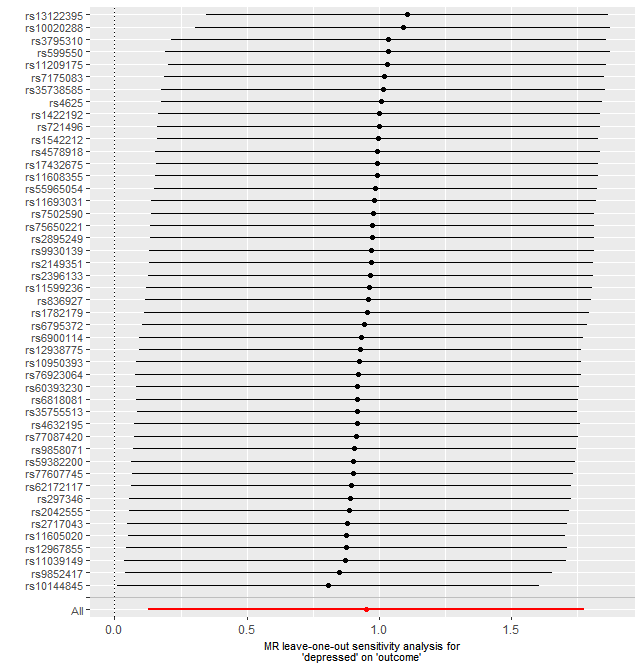

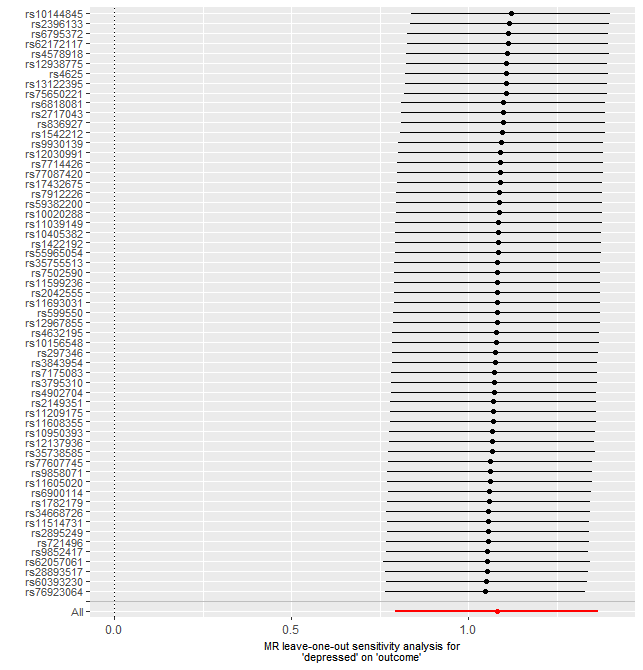


**Abbreviation:** PTSD, posttraumatic stress disorder; PD, panic disorder;

**Figure S3. Leave-one-out plot of significant results from worry on psychiatric disorders. a. worry on PTSD; b.worry on MDD;**

1. **B.**


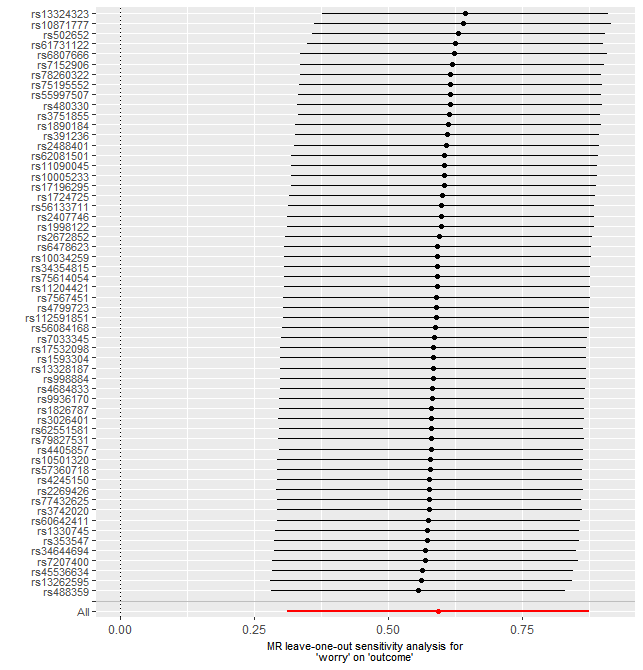

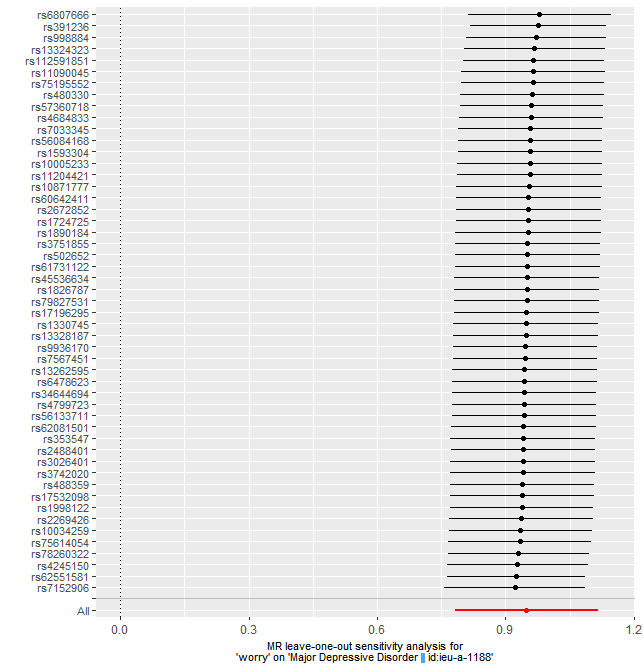


**Abbreviation:** PTSD, posttraumatic stress disorder; MDD, major depressive disorder;

**Figure S4. Funnel plot of significant results from SESA on psychiatric disorders. a. SESA on AN; b.SESA on SCZ; c. SESA on PTSD;**

1. **B.**

**
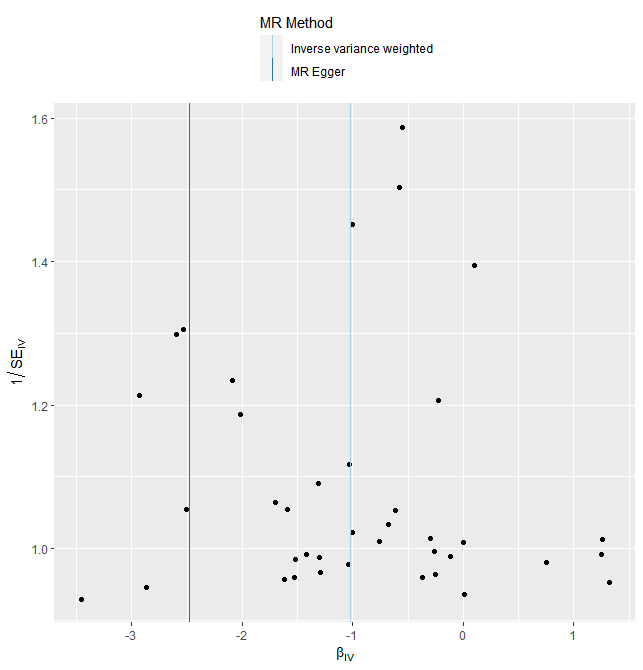

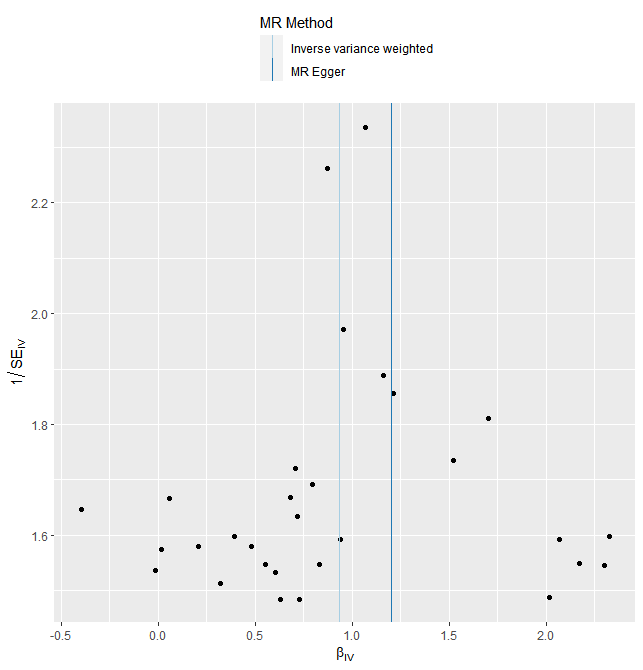
**

**C.**

**
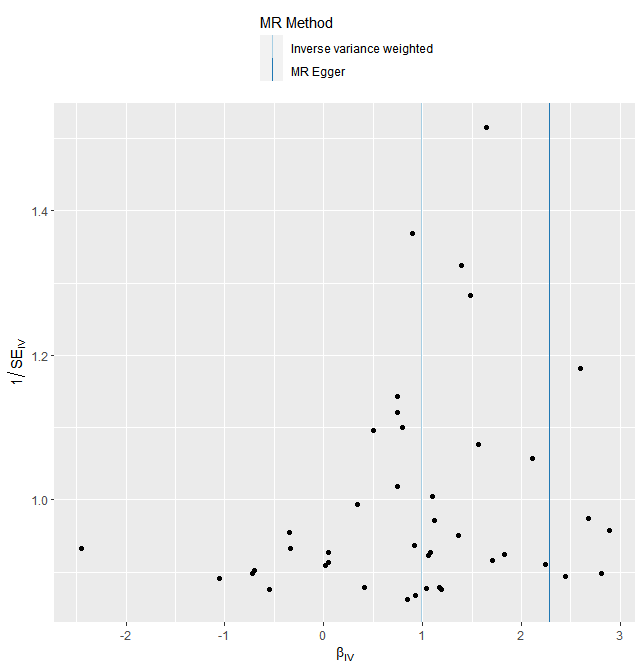
**

**Abbreviation:** SESA, sensitivity to environmental stress and adversity; AN, anorexia nervosa; SCZ, schizophrenia; PTSD, posttraumatic stress disorder;

**Figure S5. Funnel plot of significant results from depressed affect on psychiatric disorders. a. depressed affect on PD; b.depressed affect on PTSD;**

**
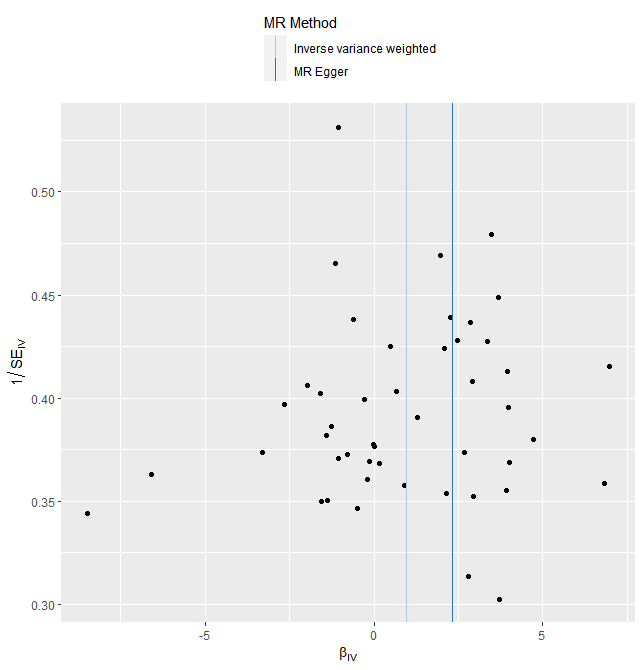

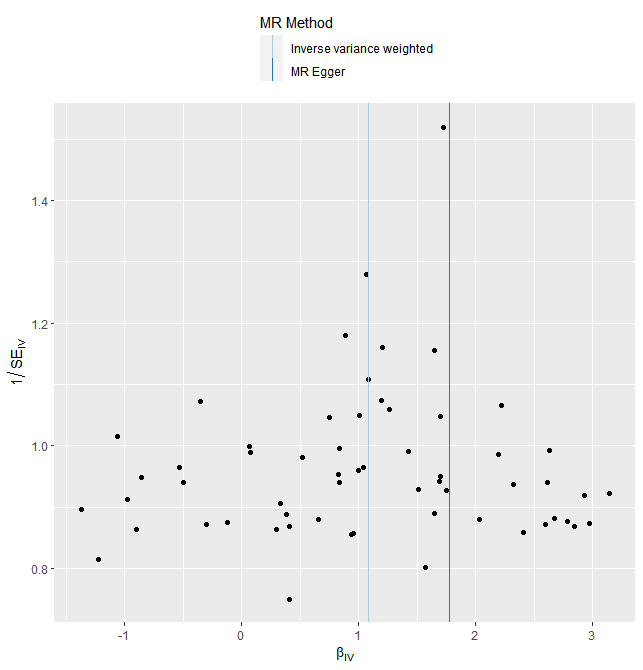
**

**Abbreviation:** PTSD, posttraumatic stress disorder; PD, panic disorder;

**Figure S6. Funnel plot of significant results from worry on psychiatric disorders. a. worry on PTSD; b.worry on MDD;**

1. **B.**

**
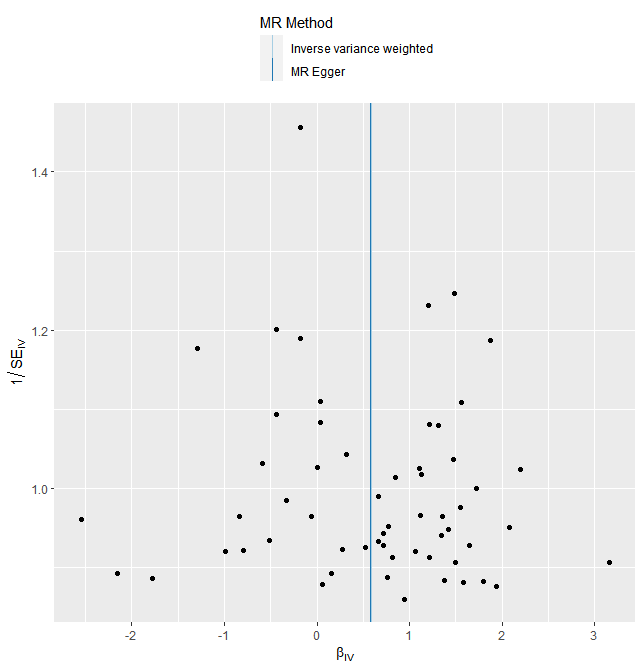

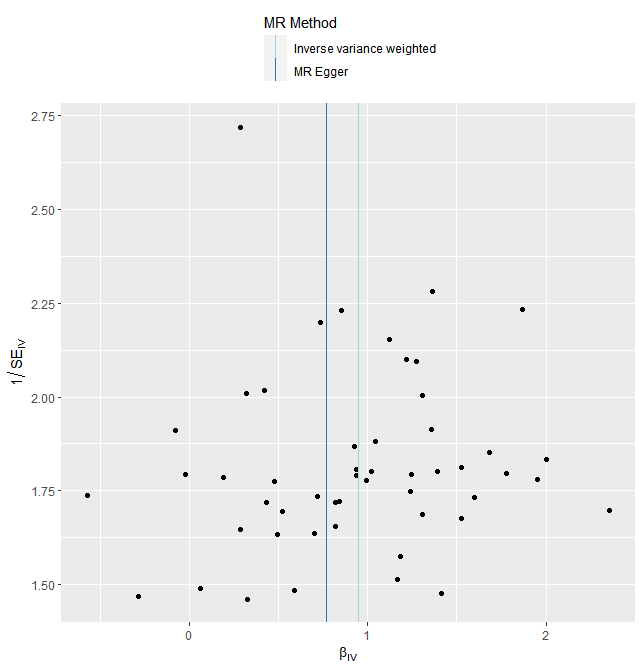
**

**Abbreviation:** PTSD, posttraumatic stress disorder; MDD, major depressive disorder;

**5.R code**

aa=read. table("neuroticism. txt", header = TRUE, sep = "\t")

exposure_dat=subset(aa, aa$P<5e-08)

exposure_dat=clump_data(exposure_dat, clump_kb= 10000, clump_r2 = 0.001,clump_p1 = 1,clump_p2 = 1)

dd=read. table ("psychiatric disorder .txt", header = TRUE, sep = "\t")

outcome_dat <- format_data(dd, type = "outcome", snp_col = "SNP", beta_col = "Beta",

se_col = "SE", effect_allele_col = "A1",other_allele_col = "A2",

pval_col = "P", chr_col="CHR")

mydata<- harmonise_data(exposure_dat = exposure_dat, outcome_dat = outcome_dat)

res <- mr(mydata,method_list =c("mr_ivw_mre","mr_egger_regression","mr_weighted_median"))

mr_presso=mr_presso(BetaOutcome ="beta.outcome", BetaExposure = "beta.exposure", SdOutcome ="se.outcome", SdExposure = "se.exposure", OUTLIERtest = TRUE, DISTORTIONtest = TRUE, data = mydata, NbDistribution = 10000,

SignifThreshold = 0.05)

OR <-generate_odds_ratios(res)

RE <-mr(mydata,method_list=c('mr_ivw_mre'))

FE <-mr(mydata,method_list=c('mr_ivw_fe'))

het <- mr_heterogeneity(mydata)

pleio <- mr_pleiotropy_test(mydata)

mr_leaveoneout_plot(single)

mr_scatter_plot(res,mydata)

res_single <- mr_singlesnp(mydata)

mr_forest_plot(res_single)

mr_funnel_plot(res_single)

**Reference**

Demontis, D., Walters, G.B., Athanasiadis, G., Walters, R., Therrien, K., Nielsen, T.T., Farajzadeh, L., Voloudakis, G., Bendl, J., Zeng, B., Zhang, W., Grove, J., Als, T.D., Duan, J., Satterstrom, F.K., Bybjerg-Grauholm, J., Bækved-Hansen, M., Gudmundsson, O.O., Magnusson, S.H., Baldursson, G., Davidsdottir, K., Haraldsdottir, G.S., Agerbo, E., Hoffman, G.E., Dalsgaard, S., Martin, J., Ribasés, M., Boomsma, D.I., Soler Artigas, M., Roth Mota, N., Howrigan, D., Medland, S.E., Zayats, T., Rajagopal, V.M., ADHD Working Group of the Psychiatric Genomics Consortium, iPSYCH-Broad Consortium, Nordentoft, M., Mors, O., Hougaard, D.M., Mortensen, P.B., Daly, M.J., Faraone, S.V., Stefansson, H., Roussos, P., Franke, B., Werge, T., Neale, B.M., Stefansson, K., Børglum, A.D., 2023. Genome-wide analyses of ADHD identify 27 risk loci, refine the genetic architecture and implicate several cognitive domains. Nat Genet 55, 198–208. https://doi.org/10.1038/s41588-022-01285-8

Eysenck, S.B.G., Eysenck, H.J., Barrett, P., 1985. A revised version of the psychoticism scale. Personality and Individual Differences 6, 21–29. https://doi.org/10.1016/0191-8869(85)90026-1

Fayyad, J., Sampson, N.A., Hwang, I., Adamowski, T., Aguilar-Gaxiola, S., Al-Hamzawi, A., Andrade, L.H.S.G., Borges, G., de Girolamo, G., Florescu, S., Gureje, O., Haro, J.M., Hu, C., Karam, E.G., Lee, S., Navarro-Mateu, F., O’Neill, S., Pennell, B.-E., Piazza, M., Posada-Villa, J., Ten Have, M., Torres, Y., Xavier, M., Zaslavsky, A.M., Kessler, R.C., WHO World Mental Health Survey Collaborators, 2017. The descriptive epidemiology of DSM-IV Adult ADHD in the World Health Organization World Mental Health Surveys. Atten Defic Hyperact Disord 9, 47–65. https://doi.org/10.1007/s12402-016-0208-3

Forstner, A.J., Awasthi, S., Wolf, C., Maron, E., Erhardt, A., Czamara, D., Eriksson, E., Lavebratt, C., Allgulander, C., Friedrich, N., Becker, J., Hecker, J., Rambau, S., Conrad, R., Geiser, F., McMahon, F.J., Moebus, S., Hess, T., Buerfent, B.C., Hoffmann, P., Herms, S., Heilmann-Heimbach, S., Kockum, I., Olsson, T., Alfredsson, L., Weber, H., Alpers, G.W., Arolt, V., Fehm, L., Fydrich, T., Gerlach, A.L., Hamm, A., Kircher, T., Pané-Farré, C.A., Pauli, P., Rief, W., Ströhle, A., Plag, J., Lang, T., Wittchen, H.-U., Mattheisen, M., Meier, S., Metspalu, A., Domschke, K., Reif, A., Hovatta, I., Lindefors, N., Andersson, E., Schalling, M., Mbarek, H., Milaneschi, Y., de Geus, E.J.C., Boomsma, D.I., Penninx, B.W.J.H., Thorgeirsson, T.E., Steinberg, S., Stefansson, K., Stefansson, H., Müller-Myhsok, B., Hansen, T.F., Børglum, A.D., Werge, T., Mortensen, P.B., Nordentoft, M., Hougaard, D.M., Hultman, C.M., Sullivan, P.F., Nöthen, M.M., Woldbye, D.P.D., Mors, O., Binder, E.B., Rück, C., Ripke, S., Deckert, J., Schumacher, J., 2021. Genome-wide association study of panic disorder reveals genetic overlap with neuroticism and depression. Mol Psychiatry 26, 4179–4190. https://doi.org/10.1038/s41380-019-0590-2

Franke, B., Michelini, G., Asherson, P., Banaschewski, T., Bilbow, A., Buitelaar, J.K., Cormand, B., Faraone, S.V., Ginsberg, Y., Haavik, J., Kuntsi, J., Larsson, H., Lesch, K.-P., Ramos-Quiroga, J.A., Réthelyi, J.M., Ribases, M., Reif, A., 2018. Live fast, die young? A review on the developmental trajectories of ADHD across the lifespan. Eur Neuropsychopharmacol 28, 1059–1088. https://doi.org/10.1016/j.euroneuro.2018.08.001

Grove, J., Ripke, S., Als, T.D., Mattheisen, M., Walters, R.K., Won, H., Pallesen, J., Agerbo, E., Andreassen, O.A., Anney, R., Awashti, S., Belliveau, R., Bettella, F., Buxbaum, J.D., Bybjerg-Grauholm, J., Bækvad-Hansen, M., Cerrato, F., Chambert, K., Christensen, J.H., Churchhouse, C., Dellenvall, K., Demontis, D., De Rubeis, S., Devlin, B., Djurovic, S., Dumont, A.L., Goldstein, J.I., Hansen, C.S., Hauberg, M.E., Hollegaard, M.V., Hope, S., Howrigan, D.P., Huang, H., Hultman, C.M., Klei, L., Maller, J., Martin, J., Martin, A.R., Moran, J.L., Nyegaard, M., Nærland, T., Palmer, D.S., Palotie, A., Pedersen, C.B., Pedersen, M.G., dPoterba, T., Poulsen, J.B., Pourcain, B.S., Qvist, P., Rehnström, K., Reichenberg, A., Reichert, J., Robinson, E.B., Roeder, K., Roussos, P., Saemundsen, E., Sandin, S., Satterstrom, F.K., Davey Smith, G., Stefansson, H., Steinberg, S., Stevens, C.R., Sullivan, P.F., Turley, P., Walters, G.B., Xu, X., Autism Spectrum Disorder Working Group of the Psychiatric Genomics Consortium, BUPGEN, Major Depressive Disorder Working Group of the Psychiatric Genomics Consortium, 23andMe Research Team, Stefansson, K., Geschwind, D.H., Nordentoft, M., Hougaard, D.M., Werge, T., Mors, O., Mortensen, P.B., Neale, B.M., Daly, M.J., Børglum, A.D., 2019. Identification of common genetic risk variants for autism spectrum disorder. Nat Genet 51, 431–444. https://doi.org/10.1038/s41588-019-0344-8

International Obsessive Compulsive Disorder Foundation Genetics Collaborative (IOCDF-GC) and OCD Collaborative Genetics Association Studies (OCGAS), 2018. Revealing the complex genetic architecture of obsessive-compulsive disorder using meta-analysis. Mol Psychiatry 23, 1181–1188. https://doi.org/10.1038/mp.2017.154

Larsson, H., Chang, Z., D’Onofrio, B.M., Lichtenstein, P., 2014. The heritability of clinically diagnosed attention deficit hyperactivity disorder across the lifespan. Psychol Med 44, 2223–2229. https://doi.org/10.1017/S0033291713002493

Mullins, N., Forstner, A.J., O’Connell, K.S., Coombes, B., Coleman, J.R.I., Qiao, Z., Als, T.D., Bigdeli, T.B., Børte, S., Bryois, J., Charney, A.W., Drange, O.K., Gandal, M.J., Hagenaars, S.P., Ikeda, M., Kamitaki, N., Kim, M., Krebs, K., Panagiotaropoulou, G., Schilder, B.M., Sloofman, L.G., Steinberg, S., Trubetskoy, V., Winsvold, B.S., Won, H.-H., Abramova, L., Adorjan, K., Agerbo, E., Al Eissa, M., Albani, D., Alliey-Rodriguez, N., Anjorin, A., Antilla, V., Antoniou, A., Awasthi, S., Baek, J.H., Bækvad-Hansen, M., Bass, N., Bauer, M., Beins, E.C., Bergen, S.E., Birner, A., Bøcker Pedersen, C., Bøen, E., Boks, M.P., Bosch, R., Brum, M., Brumpton, B.M., Brunkhorst-Kanaan, N., Budde, M., Bybjerg-Grauholm, J., Byerley, W., Cairns, M., Casas, M., Cervantes, P., Clarke, T.-K., Cruceanu, C., Cuellar-Barboza, A., Cunningham, J., Curtis, D., Czerski, P.M., Dale, A.M., Dalkner, N., David, F.S., Degenhardt, F., Djurovic, S., Dobbyn, A.L., Douzenis, A., Elvsåshagen, T., Escott-Price, V., Ferrier, I.N., Fiorentino, A., Foroud, T.M., Forty, L., Frank, J., Frei, O., Freimer, N.B., Frisén, L., Gade, K., Garnham, J., Gelernter, J., Giørtz Pedersen, M., Gizer, I.R., Gordon, S.D., Gordon-Smith, K., Greenwood, T.A., Grove, J., Guzman-Parra, J., Ha, K., Haraldsson, M., Hautzinger, M., Heilbronner, U., Hellgren, D., Herms, S., Hoffmann, P., Holmans, P.A., Huckins, L., Jamain, S., Johnson, J.S., Kalman, J.L., Kamatani, Y., Kennedy, J.L., Kittel-Schneider, S., Knowles, J.A., Kogevinas, M., Koromina, M., Kranz, T.M., Kranzler, H.R., Kubo, M., Kupka, R., Kushner, S.A., Lavebratt, C., Lawrence, J., Leber, M., Lee, H.-J., Lee, P.H., Levy, S.E., Lewis, C., Liao, C., Lucae, S., Lundberg, M., MacIntyre, D.J., Magnusson, S.H., Maier, W., Maihofer, A., Malaspina, D., Maratou, E., Martinsson, L., Mattheisen, M., McCarroll, S.A., McGregor, N.W., McGuffin, P., McKay, J.D., Medeiros, H., Medland, S.E., Millischer, V., Montgomery, G.W., Moran, J.L., Morris, D.W., Mühleisen, T.W., O’Brien, N., O’Donovan, C., Olde Loohuis, L.M., Oruc, L., Papiol, S., Pardiñas, A.F., Perry, A., Pfennig, A., Porichi, E., Potash, J.B., Quested, D., Raj, T., Rapaport, M.H., DePaulo, J.R., Regeer, E.J., Rice, J.P., Rivas, F., Rivera, M., Roth, J., Roussos, P., Ruderfer, D.M., Sánchez-Mora, C., Schulte, E.C., Senner, F., Sharp, S., Shilling, P.D., Sigurdsson, E., Sirignano, L., Slaney, C., Smeland, O.B., Smith, D.J., Sobell, J.L., Søholm Hansen, C., Soler Artigas, M., Spijker, A.T., Stein, D.J., Strauss, J.S., Świątkowska, B., Terao, C., Thorgeirsson, T.E., Toma, C., Tooney, P., Tsermpini, E.-E., Vawter, M.P., Vedder, H., Walters, J.T.R., Witt, S.H., Xi, S., Xu, W., Yang, J.M.K., Young, A.H., Young, H., Zandi, P.P., Zhou, H., Zillich, L., HUNT All-In Psychiatry, Adolfsson, R., Agartz, I., Alda, M., Alfredsson, L., Babadjanova, G., Backlund, L., Baune, B.T., Bellivier, F., Bengesser, S., Berrettini, W.H., Blackwood, D.H.R., Boehnke, M., Børglum, A.D., Breen, G., Carr, V.J., Catts, S., Corvin, A., Craddock, N., Dannlowski, U., Dikeos, D., Esko, T., Etain, B., Ferentinos, P., Frye, M., Fullerton, J.M., Gawlik, M., Gershon, E.S., Goes, F.S., Green, M.J., Grigoroiu-Serbanescu, M., Hauser, J., Henskens, F., Hillert, J., Hong, K.S., Hougaard, D.M., Hultman, C.M., Hveem, K., Iwata, N., Jablensky, A.V., Jones, I., Jones, L.A., Kahn, R.S., Kelsoe, J.R., Kirov, G., Landén, M., Leboyer, M., Lewis, C.M., Li, Q.S., Lissowska, J., Lochner, C., Loughland, C., Martin, N.G., Mathews, C.A., Mayoral, F., McElroy, S.L., McIntosh, A.M., McMahon, F.J., Melle, I., Michie, P., Milani, L., Mitchell, P.B., Morken, G., Mors, O., Mortensen, P.B., Mowry, B., Müller-Myhsok, B., Myers, R.M., Neale, B.M., Nievergelt, C.M., Nordentoft, M., Nöthen, M.M., O’Donovan, M.C., Oedegaard, K.J., Olsson, T., Owen, M.J., Paciga, S.A., Pantelis, C., Pato, C., Pato, M.T., Patrinos, G.P., Perlis, R.H., Posthuma, D., Ramos-Quiroga, J.A., Reif, A., Reininghaus, E.Z., Ribasés, M., Rietschel, M., Ripke, S., Rouleau, G.A., Saito, T., Schall, U., Schalling, M., Schofield, P.R., Schulze, T.G., Scott, L.J., Scott, R.J., Serretti, A., Shannon Weickert, C., Smoller, J.W., Stefansson, H., Stefansson, K., Stordal, E., Streit, F., Sullivan, P.F., Turecki, G., Vaaler, A.E., Vieta, E., Vincent, J.B., Waldman, I.D., Weickert, T.W., Werge, T., Wray, N.R., Zwart, J.-A., Biernacka, J.M., Nurnberger, J.I., Cichon, S., Edenberg, H.J., Stahl, E.A., McQuillin, A., Di Florio, A., Ophoff, R.A., Andreassen, O.A., 2021. Genome-wide association study of more than 40,000 bipolar disorder cases provides new insights into the underlying biology. Nat Genet 53, 817–829. https://doi.org/10.1038/s41588-021-00857-4

Nagel, M., Jansen, P.R., Stringer, S., Watanabe, K., de Leeuw, C.A., Bryois, J., Savage, J.E., Hammerschlag, A.R., Skene, N.G., Muñoz-Manchado, A.B., 23andMe Research Team, White, T., Tiemeier, H., Linnarsson, S., Hjerling-Leffler, J., Polderman, T.J.C., Sullivan, P.F., van der Sluis, S., Posthuma, D., 2018. Meta-analysis of genome-wide association studies for neuroticism in 449,484 individuals identifies novel genetic loci and pathways. Nat Genet 50, 920–927. https://doi.org/10.1038/s41588-018-0151-7

Nagel, M., Speed, D., van der Sluis, S., Østergaard, S.D., 2020. Genome-wide association study of the sensitivity to environmental stress and adversity neuroticism cluster. Acta Psychiatr Scand 141, 476–478. https://doi.org/10.1111/acps.13155

Nievergelt, C.M., Maihofer, A.X., Klengel, T., Atkinson, E.G., Chen, C.-Y., Choi, K.W., Coleman, J.R.I., Dalvie, S., Duncan, L.E., Gelernter, J., Levey, D.F., Logue, M.W., Polimanti, R., Provost, A.C., Ratanatharathorn, A., Stein, M.B., Torres, K., Aiello, A.E., Almli, L.M., Amstadter, A.B., Andersen, S.B., Andreassen, O.A., Arbisi, P.A., Ashley-Koch, A.E., Austin, S.B., Avdibegovic, E., Babić, D., Bækvad-Hansen, M., Baker, D.G., Beckham, J.C., Bierut, L.J., Bisson, J.I., Boks, M.P., Bolger, E.A., Børglum, A.D., Bradley, B., Brashear, M., Breen, G., Bryant, R.A., Bustamante, A.C., Bybjerg-Grauholm, J., Calabrese, J.R., Caldas-de-Almeida, J.M., Dale, A.M., Daly, M.J., Daskalakis, N.P., Deckert, J., Delahanty, D.L., Dennis, M.F., Disner, S.G., Domschke, K., Dzubur-Kulenovic, A., Erbes, C.R., Evans, A., Farrer, L.A., Feeny, N.C., Flory, J.D., Forbes, D., Franz, C.E., Galea, S., Garrett, M.E., Gelaye, B., Geuze, E., Gillespie, C., Uka, A.G., Gordon, S.D., Guffanti, G., Hammamieh, R., Harnal, S., Hauser, M.A., Heath, A.C., Hemmings, S.M.J., Hougaard, D.M., Jakovljevic, M., Jett, M., Johnson, E.O., Jones, I., Jovanovic, T., Qin, X.-J., Junglen, A.G., Karstoft, K.-I., Kaufman, M.L., Kessler, R.C., Khan, A., Kimbrel, N.A., King, A.P., Koen, N., Kranzler, H.R., Kremen, W.S., Lawford, B.R., Lebois, L.A.M., Lewis, C.E., Linnstaedt, S.D., Lori, A., Lugonja, B., Luykx, J.J., Lyons, M.J., Maples-Keller, J., Marmar, C., Martin, A.R., Martin, N.G., Maurer, D., Mavissakalian, M.R., McFarlane, A., McGlinchey, R.E., McLaughlin, K.A., McLean, S.A., McLeay, S., Mehta, D., Milberg, W.P., Miller, M.W., Morey, R.A., Morris, C.P., Mors, O., Mortensen, P.B., Neale, B.M., Nelson, E.C., Nordentoft, M., Norman, S.B., O’Donnell, M., Orcutt, H.K., Panizzon, M.S., Peters, E.S., Peterson, A.L., Peverill, M., Pietrzak, R.H., Polusny, M.A., Rice, J.P., Ripke, S., Risbrough, V.B., Roberts, A.L., Rothbaum, A.O., Rothbaum, B.O., Roy-Byrne, P., Ruggiero, K., Rung, A., Rutten, B.P.F., Saccone, N.L., Sanchez, S.E., Schijven, D., Seedat, S., Seligowski, A.V., Seng, J.S., Sheerin, C.M., Silove, D., Smith, A.K., Smoller, J.W., Sponheim, S.R., Stein, D.J., Stevens, J.S., Sumner, J.A., Teicher, M.H., Thompson, W.K., Trapido, E., Uddin, M., Ursano, R.J., van den Heuvel, L.L., Van Hooff, M., Vermetten, E., Vinkers, C.H., Voisey, J., Wang, Y., Wang, Z., Werge, T., Williams, M.A., Williamson, D.E., Winternitz, S., Wolf, C., Wolf, E.J., Wolff, J.D., Yehuda, R., Young, R.M., Young, K.A., Zhao, H., Zoellner, L.A., Liberzon, I., Ressler, K.J., Haas, M., Koenen, K.C., 2019. International meta-analysis of PTSD genome-wide association studies identifies sex- and ancestry-specific genetic risk loci. Nat Commun 10, 4558. https://doi.org/10.1038/s41467-019-12576-w

Polanczyk, G., de Lima, M.S., Horta, B.L., Biederman, J., Rohde, L.A., 2007. The worldwide prevalence of ADHD: a systematic review and metaregression analysis. Am J Psychiatry 164, 942–948. https://doi.org/10.1176/ajp.2007.164.6.942

Speed, D., Hemani, G., Speed, M.S., Major Depressive Disorder Working Group of the Psychiatric Genomics Consortium, Børglum, A.D., Østergaard, S.D., 2019. Investigating the causal relationship between neuroticism and depression via Mendelian randomization. Acta Psychiatr Scand 139, 395–397. https://doi.org/10.1111/acps.13009

Trubetskoy, V., Pardiñas, A.F., Qi, T., Panagiotaropoulou, G., Awasthi, S., Bigdeli, T.B., Bryois, J., Chen, C.-Y., Dennison, C.A., Hall, L.S., Lam, M., Watanabe, K., Frei, O., Ge, T., Harwood, J.C., Koopmans, F., Magnusson, S., Richards, A.L., Sidorenko, J., Wu, Y., Zeng, J., Grove, J., Kim, M., Li, Z., Voloudakis, G., Zhang, W., Adams, M., Agartz, I., Atkinson, E.G., Agerbo, E., Al Eissa, M., Albus, M., Alexander, M., Alizadeh, B.Z., Alptekin, K., Als, T.D., Amin, F., Arolt, V., Arrojo, M., Athanasiu, L., Azevedo, M.H., Bacanu, S.A., Bass, N.J., Begemann, M., Belliveau, R.A., Bene, J., Benyamin, B., Bergen, S.E., Blasi, G., Bobes, J., Bonassi, S., Braun, A., Bressan, R.A., Bromet, E.J., Bruggeman, R., Buckley, P.F., Buckner, R.L., Bybjerg-Grauholm, J., Cahn, W., Cairns, M.J., Calkins, M.E., Carr, V.J., Castle, D., Catts, S.V., Chambert, K.D., Chan, R.C.K., Chaumette, B., Cheng, W., Cheung, E.F.C., Chong, S.A., Cohen, D., Consoli, A., Cordeiro, Q., Costas, J., Curtis, C., Davidson, M., Davis, K.L., de Haan, L., Degenhardt, F., DeLisi, L.E., Demontis, D., Dickerson, F., Dikeos, D., Dinan, T., Djurovic, S., Duan, J., Ducci, G., Dudbridge, F., Eriksson, J.G., Fañanás, L., Faraone, S.V., Fiorentino, A., Forstner, A., Frank, J., Freimer, N.B., Fromer, M., Frustaci, A., Gadelha, A., Genovese, G., Gershon, E.S., Giannitelli, M., Giegling, I., Giusti-Rodríguez, P., Godard, S., Goldstein, J.I., González Peñas, J., González-Pinto, A., Gopal, S., Gratten, J., Green, M.F., Greenwood, T.A., Guillin, O., Gülöksüz, S., Gur, R.E., Gur, R.C., Gutiérrez, B., Hahn, E., Hakonarson, H., Haroutunian, V., Hartmann, A.M., Harvey, C., Hayward, C., Henskens, F.A., Herms, S., Hoffmann, P., Howrigan, D.P., Ikeda, M., Iyegbe, C., Joa, I., Julià, A., Kähler, A.K., Kam-Thong, T., Kamatani, Y., Karachanak-Yankova, S., Kebir, O., Keller, M.C., Kelly, B.J., Khrunin, A., Kim, S.-W., Klovins, J., Kondratiev, N., Konte, B., Kraft, J., Kubo, M., Kučinskas, V., Kučinskiene, Z.A., Kusumawardhani, A., Kuzelova-Ptackova, H., Landi, S., Lazzeroni, L.C., Lee, P.H., Legge, S.E., Lehrer, D.S., Lencer, R., Lerer, B., Li, M., Lieberman, J., Light, G.A., Limborska, S., Liu, C.-M., Lönnqvist, J., Loughland, C.M., Lubinski, J., Luykx, J.J., Lynham, A., Macek, M., Mackinnon, A., Magnusson, P.K.E., Maher, B.S., Maier, W., Malaspina, D., Mallet, J., Marder, S.R., Marsal, S., Martin, A.R., Martorell, L., Mattheisen, M., McCarley, R.W., McDonald, C., McGrath, J.J., Medeiros, H., Meier, S., Melegh, B., Melle, I., Mesholam-Gately, R.I., Metspalu, A., Michie, P.T., Milani, L., Milanova, V., Mitjans, M., Molden, E., Molina, E., Molto, M.D., Mondelli, V., Moreno, C., Morley, C.P., Muntané, G., Murphy, K.C., Myin-Germeys, I., Nenadić, I., Nestadt, G., Nikitina-Zake, L., Noto, C., Nuechterlein, K.H., O’Brien, N.L., O’Neill, F.A., Oh, S.-Y., Olincy, A., Ota, V.K., Pantelis, C., Papadimitriou, G.N., Parellada, M., Paunio, T., Pellegrino, R., Periyasamy, S., Perkins, D.O., Pfuhlmann, B., Pietiläinen, O., Pimm, J., Porteous, D., Powell, J., Quattrone, D., Quested, D., Radant, A.D., Rampino, A., Rapaport, M.H., Rautanen, A., Reichenberg, A., Roe, C., Roffman, J.L., Roth, J., Rothermundt, M., Rutten, B.P.F., Saker-Delye, S., Salomaa, V., Sanjuan, J., Santoro, M.L., Savitz, A., Schall, U., Scott, R.J., Seidman, L.J., Sharp, S.I., Shi, J., Siever, L.J., Sigurdsson, E., Sim, K., Skarabis, N., Slominsky, P., So, H.-C., Sobell, J.L., Söderman, E., Stain, H.J., Steen, N.E., Steixner-Kumar, A.A., Stögmann, E., Stone, W.S., Straub, R.E., Streit, F., Strengman, E., Stroup, T.S., Subramaniam, M., Sugar, C.A., Suvisaari, J., Svrakic, D.M., Swerdlow, N.R., Szatkiewicz, J.P., Ta, T.M.T., Takahashi, A., Terao, C., Thibaut, F., Toncheva, D., Tooney, P.A., Torretta, S., Tosato, S., Tura, G.B., Turetsky, B.I., Üçok, A., Vaaler, A., van Amelsvoort, T., van Winkel, R., Veijola, J., Waddington, J., Walter, H., Waterreus, A., Webb, B.T., Weiser, M., Williams, N.M., Witt, S.H., Wormley, B.K., Wu, J.Q., Xu, Z., Yolken, R., Zai, C.C., Zhou, W., Zhu, F., Zimprich, F., Atbaşoğlu, E.C., Ayub, M., Benner, C., Bertolino, A., Black, D.W., Bray, N.J., Breen, G., Buccola, N.G., Byerley, W.F., Chen, W.J., Cloninger, C.R., Crespo-Facorro, B., Donohoe, G., Freedman, R., Galletly, C., Gandal, M.J., Gennarelli, M., Hougaard, D.M., Hwu, H.-G., Jablensky, A.V., McCarroll, S.A., Moran, J.L., Mors, O., Mortensen, P.B., Müller-Myhsok, B., Neil, A.L., Nordentoft, M., Pato, M.T., Petryshen, T.L., Pirinen, M., Pulver, A.E., Schulze, T.G., Silverman, J.M., Smoller, J.W., Stahl, E.A., Tsuang, D.W., Vilella, E., Wang, S.-H., Xu, S., Indonesia Schizophrenia Consortium, PsychENCODE, Psychosis Endophenotypes International Consortium, SynGO Consortium, Adolfsson, R., Arango, C., Baune, B.T., Belangero, S.I., Børglum, A.D., Braff, D., Bramon, E., Buxbaum, J.D., Campion, D., Cervilla, J.A., Cichon, S., Collier, D.A., Corvin, A., Curtis, D., Forti, M.D., Domenici, E., Ehrenreich, H., Escott-Price, V., Esko, T., Fanous, A.H., Gareeva, A., Gawlik, M., Gejman, P.V., Gill, M., Glatt, S.J., Golimbet, V., Hong, K.S., Hultman, C.M., Hyman, S.E., Iwata, N., Jönsson, E.G., Kahn, R.S., Kennedy, J.L., Khusnutdinova, E., Kirov, G., Knowles, J.A., Krebs, M.-O., Laurent-Levinson, C., Lee, J., Lencz, T., Levinson, D.F., Li, Q.S., Liu, J., Malhotra, A.K., Malhotra, D., McIntosh, A., McQuillin, A., Menezes, P.R., Morgan, V.A., Morris, D.W., Mowry, B.J., Murray, R.M., Nimgaonkar, V., Nöthen, M.M., Ophoff, R.A., Paciga, S.A., Palotie, A., Pato, C.N., Qin, S., Rietschel, M., Riley, B.P., Rivera, M., Rujescu, D., Saka, M.C., Sanders, A.R., Schwab, S.G., Serretti, A., Sham, P.C., Shi, Y., St Clair, D., Stefánsson, H., Stefansson, K., Tsuang, M.T., van Os, J., Vawter, M.P., Weinberger, D.R., Werge, T., Wildenauer, D.B., Yu, X., Yue, W., Holmans, P.A., Pocklington, A.J., Roussos, P., Vassos, E., Verhage, M., Visscher, P.M., Yang, J., Posthuma, D., Andreassen, O.A., Kendler, K.S., Owen, M.J., Wray, N.R., Daly, M.J., Huang, H., Neale, B.M., Sullivan, P.F., Ripke, S., Walters, J.T.R., O’Donovan, M.C., Schizophrenia Working Group of the Psychiatric Genomics Consortium, 2022. Mapping genomic loci implicates genes and synaptic biology in schizophrenia. Nature 604, 502–508. https://doi.org/10.1038/s41586-022-04434-5

Watson, H.J., Yilmaz, Z., Thornton, L.M., Hübel, C., Coleman, J.R.I., Gaspar, H.A., Bryois, J., Hinney, A., Leppä, V.M., Mattheisen, M., Medland, S.E., Ripke, S., Yao, S., Giusti-Rodríguez, P., Anorexia Nervosa Genetics Initiative, Hanscombe, K.B., Purves, K.L., Eating Disorders Working Group of the Psychiatric Genomics Consortium, Adan, R.A.H., Alfredsson, L., Ando, T., Andreassen, O.A., Baker, J.H., Berrettini, W.H., Boehm, I., Boni, C., Perica, V.B., Buehren, K., Burghardt, R., Cassina, M., Cichon, S., Clementi, M., Cone, R.D., Courtet, P., Crow, S., Crowley, J.J., Danner, U.N., Davis, O.S.P., de Zwaan, M., Dedoussis, G., Degortes, D., DeSocio, J.E., Dick, D.M., Dikeos, D., Dina, C., Dmitrzak-Weglarz, M., Docampo, E., Duncan, L.E., Egberts, K., Ehrlich, S., Escaramís, G., Esko, T., Estivill, X., Farmer, A., Favaro, A., Fernández-Aranda, F., Fichter, M.M., Fischer, K., Föcker, M., Foretova, L., Forstner, A.J., Forzan, M., Franklin, C.S., Gallinger, S., Giegling, I., Giuranna, J., Gonidakis, F., Gorwood, P., Mayora, M.G., Guillaume, S., Guo, Y., Hakonarson, H., Hatzikotoulas, K., Hauser, J., Hebebrand, J., Helder, S.G., Herms, S., Herpertz-Dahlmann, B., Herzog, W., Huckins, L.M., Hudson, J.I., Imgart, H., Inoko, H., Janout, V., Jiménez-Murcia, S., Julià, A., Kalsi, G., Kaminská, D., Kaprio, J., Karhunen, L., Karwautz, A., Kas, M.J.H., Kennedy, J.L., Keski-Rahkonen, A., Kiezebrink, K., Kim, Y.-R., Klareskog, L., Klump, K.L., Knudsen, G.P.S., La Via, M.C., Le Hellard, S., Levitan, R.D., Li, D., Lilenfeld, L., Lin, B.D., Lissowska, J., Luykx, J., Magistretti, P.J., Maj, M., Mannik, K., Marsal, S., Marshall, C.R., Mattingsdal, M., McDevitt, S., McGuffin, P., Metspalu, A., Meulenbelt, I., Micali, N., Mitchell, K., Monteleone, A.M., Monteleone, P., Munn-Chernoff, M.A., Nacmias, B., Navratilova, M., Ntalla, I., O’Toole, J.K., Ophoff, R.A., Padyukov, L., Palotie, A., Pantel, J., Papezova, H., Pinto, D., Rabionet, R., Raevuori, A., Ramoz, N., Reichborn-Kjennerud, T., Ricca, V., Ripatti, S., Ritschel, F., Roberts, M., Rotondo, A., Rujescu, D., Rybakowski, F., Santonastaso, P., Scherag, A., Scherer, S.W., Schmidt, U., Schork, N.J., Schosser, A., Seitz, J., Slachtova, L., Slagboom, P.E., Slof-Op ’t Landt, M.C.T., Slopien, A., Sorbi, S., Świątkowska, B., Szatkiewicz, J.P., Tachmazidou, I., Tenconi, E., Tortorella, A., Tozzi, F., Treasure, J., Tsitsika, A., Tyszkiewicz-Nwafor, M., Tziouvas, K., van Elburg, A.A., van Furth, E.F., Wagner, G., Walton, E., Widen, E., Zeggini, E., Zerwas, S., Zipfel, S., Bergen, A.W., Boden, J.M., Brandt, H., Crawford, S., Halmi, K.A., Horwood, L.J., Johnson, C., Kaplan, A.S., Kaye, W.H., Mitchell, J.E., Olsen, C.M., Pearson, J.F., Pedersen, N.L., Strober, M., Werge, T., Whiteman, D.C., Woodside, D.B., Stuber, G.D., Gordon, S., Grove, J., Henders, A.K., Juréus, A., Kirk, K.M., Larsen, J.T., Parker, R., Petersen, L., Jordan, J., Kennedy, M., Montgomery, G.W., Wade, T.D., Birgegård, A., Lichtenstein, P., Norring, C., Landén, M., Martin, N.G., Mortensen, P.B., Sullivan, P.F., Breen, G., Bulik, C.M., 2019. Genome-wide association study identifies eight risk loci and implicates metabo-psychiatric origins for anorexia nervosa. Nat Genet 51, 1207–1214. https://doi.org/10.1038/s41588-019-0439-2

Wray, N.R., Ripke, S., Mattheisen, M., Trzaskowski, M., Byrne, E.M., Abdellaoui, A., Adams, M.J., Agerbo, E., Air, T.M., Andlauer, T.M.F., Bacanu, S.-A., Bækvad-Hansen, M., Beekman, A.F.T., Bigdeli, T.B., Binder, E.B., Blackwood, D.R.H., Bryois, J., Buttenschøn, H.N., Bybjerg-Grauholm, J., Cai, N., Castelao, E., Christensen, J.H., Clarke, T.-K., Coleman, J.I.R., Colodro-Conde, L., Couvy-Duchesne, B., Craddock, N., Crawford, G.E., Crowley, C.A., Dashti, H.S., Davies, G., Deary, I.J., Degenhardt, F., Derks, E.M., Direk, N., Dolan, C.V., Dunn, E.C., Eley, T.C., Eriksson, N., Escott-Price, V., Kiadeh, F.H.F., Finucane, H.K., Forstner, A.J., Frank, J., Gaspar, H.A., Gill, M., Giusti-Rodríguez, P., Goes, F.S., Gordon, S.D., Grove, J., Hall, L.S., Hannon, E., Hansen, C.S., Hansen, T.F., Herms, S., Hickie, I.B., Hoffmann, P., Homuth, G., Horn, C., Hottenga, J.-J., Hougaard, D.M., Hu, M., Hyde, C.L., Ising, M., Jansen, R., Jin, F., Jorgenson, E., Knowles, J.A., Kohane, I.S., Kraft, J., Kretzschmar, W.W., Krogh, J., Kutalik, Z., Lane, J.M., Li, Yihan, Li, Yun, Lind, P.A., Liu, X., Lu, L., MacIntyre, D.J., MacKinnon, D.F., Maier, R.M., Maier, W., Marchini, J., Mbarek, H., McGrath, P., McGuffin, P., Medland, S.E., Mehta, D., Middeldorp, C.M., Mihailov, E., Milaneschi, Y., Milani, L., Mill, J., Mondimore, F.M., Montgomery, G.W., Mostafavi, S., Mullins, N., Nauck, M., Ng, B., Nivard, M.G., Nyholt, D.R., O’Reilly, P.F., Oskarsson, H., Owen, M.J., Painter, J.N., Pedersen, C.B., Pedersen, M.G., Peterson, R.E., Pettersson, E., Peyrot, W.J., Pistis, G., Posthuma, D., Purcell, S.M., Quiroz, J.A., Qvist, P., Rice, J.P., Riley, B.P., Rivera, M., Saeed Mirza, S., Saxena, R., Schoevers, R., Schulte, E.C., Shen, L., Shi, J., Shyn, S.I., Sigurdsson, E., Sinnamon, G.B.C., Smit, J.H., Smith, D.J., Stefansson, H., Steinberg, S., Stockmeier, C.A., Streit, F., Strohmaier, J., Tansey, K.E., Teismann, H., Teumer, A., Thompson, W., Thomson, P.A., Thorgeirsson, T.E., Tian, C., Traylor, M., Treutlein, J., Trubetskoy, V., Uitterlinden, A.G., Umbricht, D., Van der Auwera, S., van Hemert, A.M., Viktorin, A., Visscher, P.M., Wang, Y., Webb, B.T., Weinsheimer, S.M., Wellmann, J., Willemsen, G., Witt, S.H., Wu, Y., Xi, H.S., Yang, J., Zhang, F., eQTLGen, 23andMe, Arolt, V., Baune, B.T., Berger, K., Boomsma, D.I., Cichon, S., Dannlowski, U., de Geus, E.C.J., DePaulo, J.R., Domenici, E., Domschke, K., Esko, T., Grabe, H.J., Hamilton, S.P., Hayward, C., Heath, A.C., Hinds, D.A., Kendler, K.S., Kloiber, S., Lewis, G., Li, Q.S., Lucae, S., Madden, P.F.A., Magnusson, P.K., Martin, N.G., McIntosh, A.M., Metspalu, A., Mors, O., Mortensen, P.B., Müller-Myhsok, B., Nordentoft, M., Nöthen, M.M., O’Donovan, M.C., Paciga, S.A., Pedersen, N.L., Penninx, B.W.J.H., Perlis, R.H., Porteous, D.J., Potash, J.B., Preisig, M., Rietschel, M., Schaefer, C., Schulze, T.G., Smoller, J.W., Stefansson, K., Tiemeier, H., Uher, R., Völzke, H., Weissman, M.M., Werge, T., Winslow, A.R., Lewis, C.M., Levinson, D.F., Breen, G., Børglum, A.D., Sullivan, P.F., Major Depressive Disorder Working Group of the Psychiatric Genomics Consortium, 2018. Genome-wide association analyses identify 44 risk variants and refine the genetic architecture of major depression. Nat Genet 50, 668–681. https://doi.org/10.1038/s41588-018-0090-3

Yu, D., Sul, J.H., Tsetsos, F., Nawaz, M.S., Huang, A.Y., Zelaya, I., Illmann, C., Osiecki, L., Darrow, S.M., Hirschtritt, M.E., Greenberg, E., Muller-Vahl, K.R., Stuhrmann, M., Dion, Y., Rouleau, G., Aschauer, H., Stamenkovic, M., Schlögelhofer, M., Sandor, P., Barr, C.L., Grados, M., Singer, H.S., Nöthen, M.M., Hebebrand, J., Hinney, A., King, R.A., Fernandez, T.V., Barta, C., Tarnok, Z., Nagy, P., Depienne, C., Worbe, Y., Hartmann, A., Budman, C.L., Rizzo, R., Lyon, G.J., McMahon, W.M., Batterson, J.R., Cath, D.C., Malaty, I.A., Okun, M.S., Berlin, C., Woods, D.W., Lee, P.C., Jankovic, J., Robertson, M.M., Gilbert, D.L., Brown, L.W., Coffey, B.J., Dietrich, A., Hoekstra, P.J., Kuperman, S., Zinner, S.H., Luðvigsson, P., Sæmundsen, E., Thorarensen, Ó., Atzmon, G., Barzilai, N., Wagner, M., Moessner, R., Ophoff, R., Pato, C.N., Pato, M.T., Knowles, J.A., Roffman, J.L., Smoller, J.W., Buckner, R.L., Willsey, A.J., Tischfield, J.A., Heiman, G.A., Stefansson, H., Stefansson, K., Posthuma, D., Cox, N.J., Pauls, D.L., Freimer, N.B., Neale, B.M., Davis, L.K., Paschou, P., Coppola, G., Mathews, C.A., Scharf, J.M., Tourette Association of America International Consortium for Genetics, the Gilles de la Tourette GWAS Replication Initiative, the Tourette International Collaborative Genetics Study, and the Psychiatric Genomics Consortium Tourette Syndrome Working Group, 2019. Interrogating the Genetic Determinants of Tourette’s Syndrome and Other Tic Disorders Through Genome-Wide Association Studies. Am J Psychiatry 176, 217–227. https://doi.org/10.1176/appi.ajp.2018.18070857
